# Guiding the Design of Multifunctional Covalent Organic Frameworks: High-Throughput Screening of Thermal and Mechanical Properties

**DOI:** 10.1021/acs.chemmater.5c02310

**Published:** 2025-10-21

**Authors:** Sandip Thakur, Ashutosh Giri

**Affiliations:** Department of Mechanical, Industrial, and Systems Engineering, 4260 University of Rhode Island, Kingston, Rhode Island 02881, United States

## Abstract

Covalent organic frameworks (COFs) are crystalline, porous polymers with exceptional structural tunability and low density, making them ideal candidates for diverse applications, including gas storage, catalysis, electronics, and thermal management. However, their widespread use is often hindered by limited thermal and mechanical stabilities—properties that are not well understood across the vast COF chemical space. In this work, we perform a comprehensive high-throughput screening of over 38,000 2D and 3D COFs, comprising more than 1,000 unique organic linkers, to explore their mechanical stiffness and thermal conductivity through large-scale atomistic simulations. Our results reveal that COFs span an extraordinarily wide property space, with thermal conductivities ranging from ∼0.02 W m^–1^ K^–1^ to ∼50 W m^–1^ K^–1^ and bulk moduli from less than 0.1 to 100 GPa. Surprisingly, we discover that high thermal conductivity can arise not only in stiff frameworks but also in mechanically flexible COFs through directional alignment and anisotropy. Flexible COFs with carbon–carbon or carbon–nitrogen linkages, moderate-to-high densities, and low or intermediate void fractions exhibit ultrahigh thermal conductivities with phonon mean free paths that can extend up to several hundred nanometers when polymeric chains are well-aligned along the transport direction. These findings overturn conventional assumptions linking stiffness to thermal transport and demonstrate that structure–property relationships in COFs are highly tunable via chemical composition and topology. By establishing predictive design rules for achieving multifunctionality in porous polymers, this study provides a valuable roadmap for developing next-generation COFs with tailored thermal and mechanical performance.

## Graphical abstract

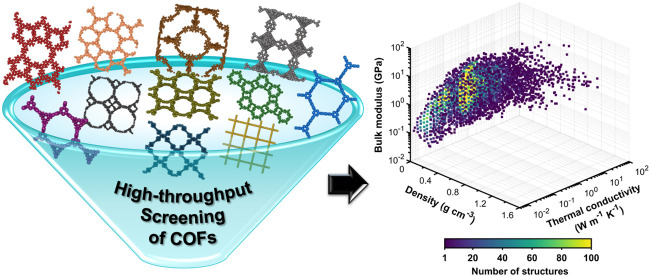

## Introduction

Covalent organic frameworks (COFs) represent a rapidly growing class of crystalline, porous polymers composed of light elements such as hydrogen, boron, carbon, nitrogen, and oxygen, interconnected by strong covalent bonds.^1–7^ Their structural versatility—stemming from a wide variety of molecular building blocks and linkage types—enables precise customization for specific applications, including gas storage,^4,5,8–12^ catalysis,^13–18^ electronics,^19–24^ and thermal management.^25–33^ This exceptional modularity, combined with their inherently high surface areas and nanoporous architectures, positions COFs as ideal materials for such uses.^2,30,34–39^ Nevertheless, a significant challenge to their widespread implementation lies in their mechanical and thermal fragility—an issue intrinsically tied to the very porosity that makes them so appealing. For example, in gas storage applications, chemical reactions and mechanical stresses during gas loading can lead to their degradation and loss of crystallinity.^35,40–43^ Additionally, the heat released during adsorption of guest molecules at high loading densities can elevate internal temperatures to several hundred Kelvin, highlighting the need for COFs with improved thermal conductivity and mechanical stability.^44,45^

To date, much of the insight into the mechanical behavior of porous frameworks has come from studies of metal–organic frameworks (MOFs), which share structural similarities with COFs. In MOFs, extensive computational and experimental investigations have elucidated how topology, metal–ligand coordination, and pore geometry influence mechanical properties and structural integrity under stress.^46–59^ In contrast, the mechanical characterization of COFs, both experimentally and computationally, remains comparatively under-explored. For 2D COFs, recent studies have demonstrated that mechanical properties are strongly influenced by linkage chemistry, topology,^60,61^ interlayer stacking,^62^ and structural defects.^63,64^ For 3D COFs, only a handful of studies have reported bulk modulus values for representative structures, typically in the range of 5 to 20 GPa, comparable to or slightly lower than those of MOFs with similar porosities.^65^ Furthermore, prior investigations have shown that swiveling motion of the functional groups in 2D COFs,^30^ and framework interpenetration in 3D COFs,^66^ can significantly enhance the mechanical properties.

While these foundational studies have advanced our understanding of the mechanical properties of COFs, they have primarily focused on a limited set of structures. Due to the vast structural and chemical diversity of COFs, spanning thousands of unique building blocks, topologies, and pore architectures, the broader mechanical property space remains largely uncharted. In particular, large-scale quantification of structure–property relationships for mechanical behavior is still lacking, posing a significant hurdle to the rational design of COFs with tailored mechanical strength and stability.

Beyond mechanical robustness, the ability of COFs to efficiently manage heat is critical to their functionality in a wide range of applications. Similar to the mechanical properties, much of the early insight into thermal transport in porous crystalline materials has come from studies on MOFs and zeolites. These materials typically exhibit glass-like thermal conductivities in the range of ∼ 0.3 to 1 W m^–1^ K^–1^,^27,28,44,45,67–86^ largely due to strong phonon scattering caused by large atomic mass mismatches between heavy metal nodes and lightweight organic linkers.^70,87^ In contrast, COFs, composed entirely of light elements interconnected through extended covalent linkages, present a promising alternative for engineering materials with both high and low thermal conductivities as we discuss in more detail below.

A recent experimental work by Evans et al. provided the first quantitative measurement of anisotropic thermal transport in boronate ester-linked 2D COFs, revealing that thermal conductivity along the bonded in-plane direction can be up to four times higher than in the cross-plane direction with the van der Waals interactions.^36^ This finding highlighted the critical role of bonding geometry and pore connectivity in enhancing thermal transport in COFs. Building on this, numerous subsequent works have employed molecular dynamics (MD) simulations to explore how structural parameters influence phonon transport, despite limitations related to empirical force fields. These studies have shown that thermal conductivity in COFs can span a broad range, driven by factors such as density, pore architecture, interpenetration, and linker flexibility.^29,30,66,88–97^ For example, gas infiltration into the pores of 2D COFs has been shown to either enhance or suppress thermal conductivity depending on pore size and gas-framework interactions, by modifying the intrinsic vibrational scattering mechanisms.^29,92^ Similarly, increased density, achieved through design or interpenetration, has been identified as a reliable strategy to improve heat transport.^30,94,95,97^ Notably, our recent work revealed that interpenetration of the COF-300 framework leads to a drastic increase in thermal conductivity—by up to 6-fold for a 3-fold interpenetrated structure, attributed to supramolecular interactions that reduce phonon scattering and enhance lattice stiffness.^66^

While the studies mentioned above have contributed valuable insights into the mechanistic understanding of thermal and mechanical properties of COFs, they have primarily examined a limited number of structures. As a result, it remains challenging to generalize these findings across the broader landscape of COFs. Considering the vast combinatorial design space of COFs, relying on a trial-and-error—or “Edisonian”—approach to develop COFs with specific thermal and mechanical properties is both time-consuming and resource-intensive. To bridge this gap, a systematic, high-throughput approach is needed to evaluate thermal and mechanical properties across thousands of 2D and 3D COF structures. In our previous work,^97^ we took a step in this direction by screening thermal conductivities for over 10,000 3D COFs from the Mercado database^98^ and revealed valuable insights into their structure–property relationships, but remained limited in scope to a single database and dimensionality.

To this end, the present work significantly broadens the scope of thermal and mechanical property evaluation in porous materials by conducting a comprehensive high-throughput screening of over 38,000 COFs, spanning both 2D and 3D architectures. These structures, sourced from the Mercado and ReDD-COFFEE databases, were systematically analyzed using MD simulations and automated workflows for property extraction. Our goal is to uncover robust structure–property relationships that can inform the rational design of COFs with tunable thermal and mechanical performance. This large-scale, data-driven approach offers a unified framework for optimizing multifunctional behavior in porous organic materials. Notably, we demonstrate that high thermal conductivity is not necessarily linked to mechanical stiffness—challenging the conventional view that stiffer materials, such as diamond, inherently exhibit better thermal transport. In COFs, mechanical strength, quantified by the bulk modulus, is strongly influenced by the flexibility of the linkers, whereas thermal conductivity is primarily governed by the degree of chain alignment along the direction of heat flow.

We identify 49 distinct 3D COFs with thermal conductivities exceeding 5 W m^–1^ K^–1^, and find that their thermal conductivities generally scale more favorably with density compared to 3D metal–organic frameworks (MOFs). In contrast, the bulk modulus spans a broader range in COFs, reflecting their greater flexibility at a given density. Nonetheless, some 3D COFs can match or even exceed the stiffness of MOFs, with bulk modulus values reaching up to 100 GPa. Importantly, we find that high thermal conductivity can be achieved in both mechanically stiff and flexible COFs. In flexible COFs, this behavior is enabled by key structural features such as pronounced anisotropy, well-aligned polymeric chains, and largest pore diameters (LPDs) below 3.5 nm or within an optimal range of 0.4 to 3 nm. Furthermore, specific functional motifs—such as triazine, boroxine, benzobisoxazole, and carbon–carbon linkages—are consistently associated with enhanced thermal transport both for 2D and 3D COFs. Finally, spectral analysis reveals that COFs with high thermal conductivity exhibit phonon mean free paths exceeding 500 nm, a remarkable result for polymeric materials, which typically display phonon mean free paths under 10 nm. These findings underscore the potential of COFs as a highly versatile platform for engineering lightweight materials with independently tunable mechanical and thermal properties.

## Results and Discussion

### Thermal and Mechanical Properties of 3D COFs

Figure 1a shows the average thermal conductivity (κ_avg_) values calculated for 26,700 three-dimensional COFs, compiled from the Mercado^98^ and ReDDCOFFEE^117^ databases. Note, κ_avg_ represents the average of thermal conductivities along all three principal directions (*x*-, *y*-, and *z*-directions) for 3D COFs. Remarkably, 95% of these structures exhibit κ_avg_ values below 1 W m^–1^ K^–1^, while 49 structures show values exceeding 5 W m^–1^ K^–1^ (Figure 1b). Interestingly, when compared to findings from a separate high-throughput screening of MOFs by Islamov et al.,^70^ COFs tend to exhibit higher thermal conductivities at similar mass densities, even though their maximum achievable densities are comparatively lower. This difference has been previously attributed to the pronounced mass contrast between heavy metal nodes and light organic linkers in MOFs, whereas COFs are typically composed of lighter elements such as carbon, silicon, boron, and nitrogen.^97^

**Figure 1:**
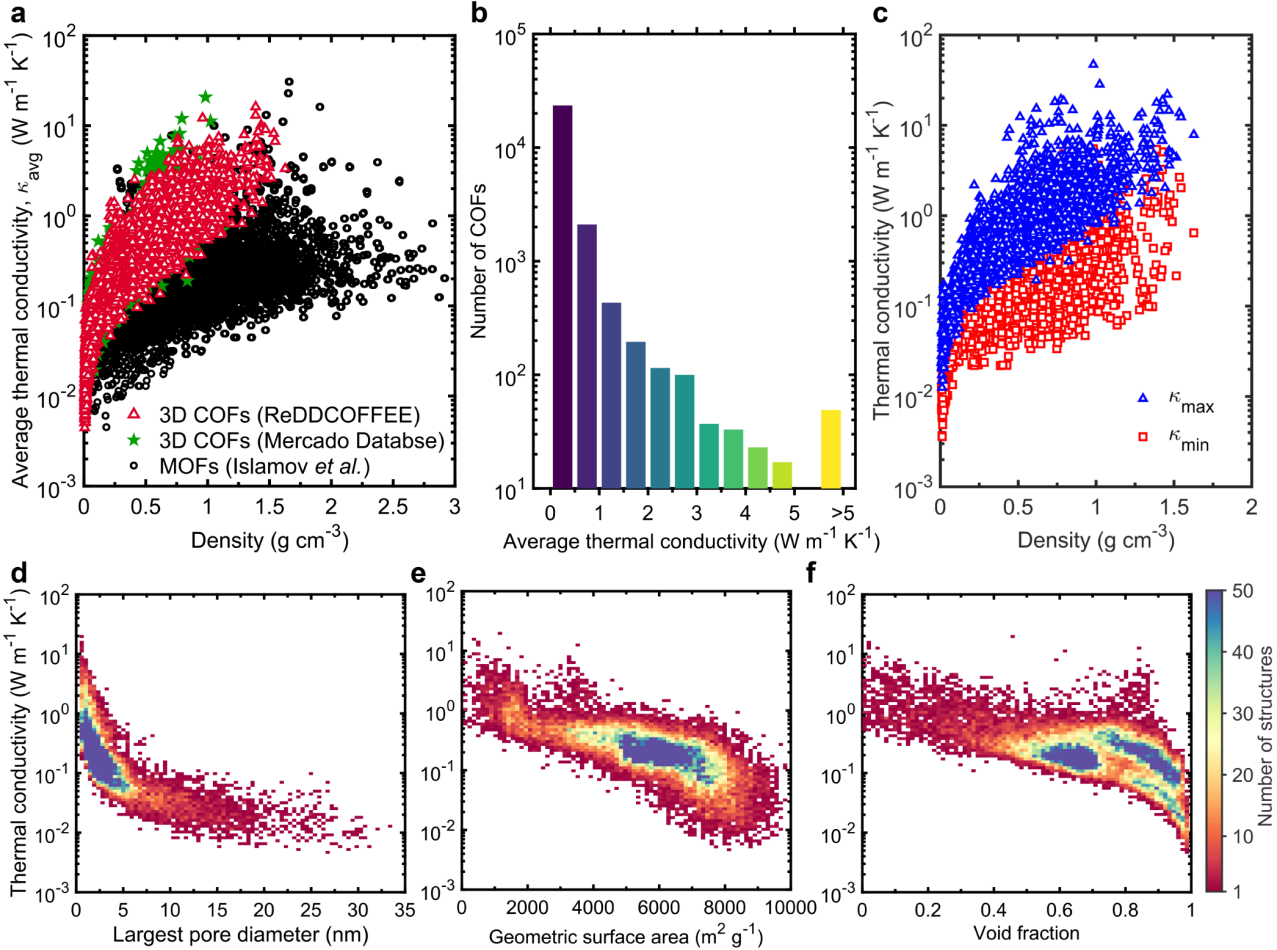
(a) Comparison of average thermal conductivity values of 10,750 (Mercado database) and 15,950 (ReDDCOFFEE database) 3D COFs with the average thermal conductivity values of 3D MOFs from Islamov et al.^70^ as a function of their density. Throughout the density range COFs exhibit higher thermal conductivity values compared to MOFs. (b) The distribution of average thermal conductivity values for the total 26,700 3D COFs combining both the Mercado and ReDDCOFFEE databases, with a bin size of 0.5 W m^–1^ K^–1^. Notably, we observe that 95% of the structures have average thermal conductivity values below 1 W m^–1^ K^–1^, while 49 structures exhibit κ_avg_ greater than 5 W m^–1^ K^–1^. (c) Maximum and minimum thermal conductivities as a function of density for 5,646 anisotropic COFs with thermal conductivity anisotropy ratio of ≥ 2. Average thermal conductivity of 3D COFs from both the Mercado and ReDDCOFFEE databases as a function of (d) largest pore diameter, (e) geometric surface area, and (f) void fraction, color-mapped according to number of structures discretized into 75 × 75 bins.

While Figure 1a shows average thermal conductivities, it is important to note that COFs often display anisotropic thermal transport, particularly those with higher thermal conductivities. If we instead consider the maximum directional thermal conductivity, we find that 136 COFs surpass 5 W m^–1^ K^–1^. The anisotropy ratio in some cases reaches ∼50, and the identities of these COFs are listed in Table S2. Such materials are especially promising for applications requiring directional heat management—for instance, enabling efficient heat removal in one direction while suppressing heat flow in others. This is particularly beneficial in microelectronic thermal management scenarios, where vertical dissipation from heat-intensive layers (e.g., processor cores) is essential, while lateral heat spreading must be minimized.^99^

We investigate the influence of various internal structural features—and their optimized combinations—on the thermal conductivity of our 3D COFs. To this end, we analyze the variation of average thermal conductivity with respect to the largest pore diameter (LPD), void fraction, and geometric surface area (GSA), as shown in Figure 1d–f. As expected and consistent with previous studies,^70,97^ larger LPD values correspond to lower thermal conductivities. Specifically, COFs with relatively high thermal conductivities tend to have LPDs smaller than 3.5 nm (Figure 1d), which broadens the scope of high thermal conductivity candidates compared to earlier results from the Mercado database, where only LPDs under 1.5 nm were associated with enhanced conductivity.^97^ Similarly, COFs with GSAs below 4000 m^2^ g^–1^ comprise the majority of structures with elevated thermal conductivities. In line with previous findings from smaller data sets in our previous work,^97^ COFs with void fractions between 0.6 and 0.9 generally exhibit higher thermal conductivities. However, we also observe that COFs with void fractions below 0.2 can achieve comparably high thermal conductivities, expanding the design space.

In parallel with thermal conductivity, we find that the bulk modulus of COFs also scales with mass density as shown in Figure 2a. However, bulk modulus values exhibit greater variability at a given density compared to thermal conductivity (Figure 1a). When compared with MOFs, this broader distribution could also stem from the smaller number of MOFs sampled in previous studies.^46^ Interestingly, we identify eight COFs with bulk modulus values exceeding 50 GPa—comparable to ceramics and silicate glasses (see Table S1 for the details of their physical attributes). This is notable given that COFs, being polymeric in nature, typically exhibit bulk moduli below 10 GPa, which remains true for the majority of the data set.

**Figure 2:**
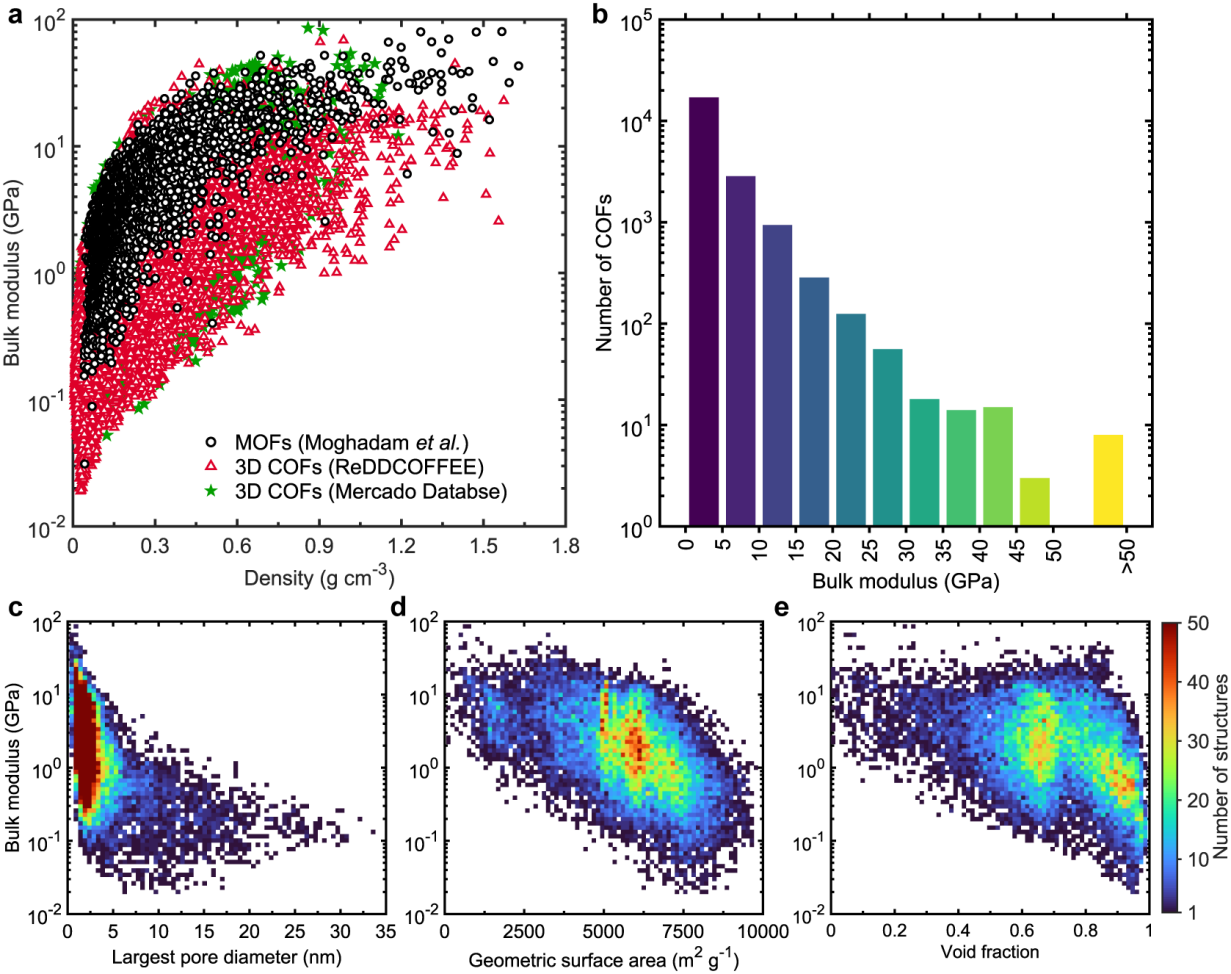
(a) Comparison of bulk modulus values of 7,645 (Mercado database) and 13,780 (ReDDCOFFEE database) 3D COFs with the bulk modulus values of 3D MOFs from Moghadam et al.^46^ as a function of their density. Overall, MOFs exhibit higher bulk modulus values compared to COFs. (b) The distribution of bulk modulus values of 21,425 3D COFs combining both the ReDDCOFFEE and Mercado databases, with a bin size of 5 GPa. Notably, we observe that 93% of the structures have bulk modulus values below 10 GPa, while 8 structures (combining both the database) exhibit bulk modulus greater than 50 GPa. Bulk modulus of 3D COFs from both the Mercado and ReDDCOFFEE databases as a function of (c) largest pore diameter, (d) geometric surface area, and (e) void fraction, color-mapped according to number of structures discretized into 75 × 75 bins.

We also observe that COFs with largest pore diameters below 3.5 nm tend to exhibit higher bulk moduli. However, unlike thermal conductivity, a small LPD does not guarantee high stiffness—many COFs with largest pore diameters below 3.5 nm still show very low bulk modulus values. Additionally, unlike thermal conductivity, parameters such as GSA and void fraction are poor predictors of mechanical stiffness, as evidenced by the wide variability in bulk modulus across these parameters (Figure 2c–e). For instance, at a void fractions of ∼ 0.6, and GSAs of ∼ 6000 m^2^ g^–1^, the bulk modulus can vary by more than 2 orders of magnitude. This suggests that, unlike thermal conductivity, achieving high mechanical stiffness in 3D COFs does not require tightly constrained values for density, void fraction, or surface area. As we discuss later, a different structural parameter emerges as a critical determinant of mechanical stability.

To explore the overlap between mechanical and thermal performance, we plot bulk modulus as a function of thermal conductivity for all 3D COFs (Figure 3a–d). As expected, high thermal conductivity tends to coincide with high bulk modulus. However, the broad spread in the data shows that a high bulk modulus is not essential for achieving high thermal conductivity. For example, COFs with bulk modulus above 10 GPa span a wide thermal conductivity range of ∼ 0.2 to 20 W m^–1^ K^–1^. This reveals distinct regions within the structure–property landscape that merit further investigation. More specifically, we separate the data set into six regions with different combinations of thermal and mechanical properties as shown in Figure 3c. Regions 1 and 2, for example, consist of COFs with both ultralow thermal conductivities and extremely low bulk moduli below 0.3 GPa (Figure 3c), suggesting fundamentally different structural characteristics associated with very large pores in these regions.

**Figure 3:**
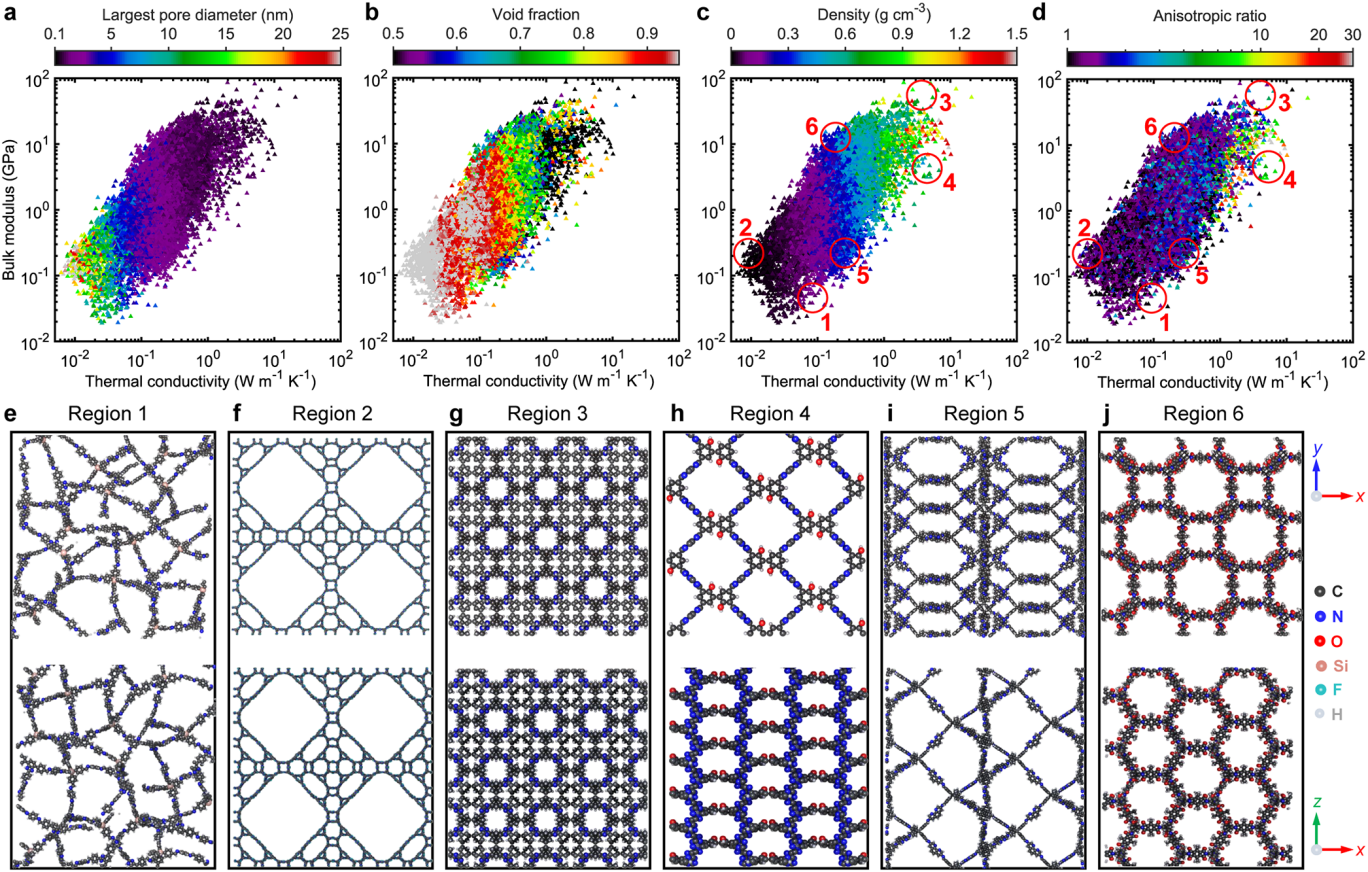
Bulk modulus as a function of average thermal conductivity values, color-mapped by (a) Largest pore diameter (LPD), (b) void fraction, (c) density, and (d) anisotropic ratio. Schematic illustrations of 3D COF structures from different regions shown in Figure 3c. (e) Region 1: low bulk modulus and low κ_avg_. (f) Region 2: high LPD, low bulk modulus and low κ_avg_. (g) Region 3: high bulk modulus and high κ_avg_. (h) Region 4: reduced bulk modulus, similar κ_avg_, and density as Region 3. (i) Region 5: low bulk modulus and low κ_avg_ with intermediate density. (j) Region 6: high bulk modulus, similar κ_avg_, and density as Region 5.

In Region 1, most COFs feature nonperiodic and misaligned pores (see Figure 3e), yet their void fractions are lower than those in Region 2, which has well-ordered pore structures (Figure 3f) and relatively higher bulk moduli. This indicates that having well-aligned pores is not essential for achieving moderate thermal conductivity in the low-conductivity regime. Regions 3 and 4 display the highest thermal conductivities, attributed to their high densities and well-aligned polymeric chains, with bulk moduli spanning a wide range from around 2 to 85 GPa. Although the COFs in both regions share similar structural characteristics in terms of LPD, GSA, void fraction, and density (Figures 3a–c and S5), Region 3 exhibits higher bulk moduli. The key distinction lies in the flexibility of the polymer chains—those in Region 4 are more flexible, while Region 3 consists of stiffer chains.

A very clear relationship emerges when considering the anisotropic ratio of thermal conductivity of the COFs (Figure 3d). The anisotropic ratio in thermal transport in the structures is translated to the anisotropy in their morphologies (due to, for example, different pore sizes and angles between polymer chains along the different directions). As shown in Figures 3d,g,h, S6 and S7, for the high thermal conductivity regions (3 and 4), the flexible COFs in region 4 demonstrate strong anisotropy, whereas the COFs in the high thermal conductivity high bulk moduli region are isotropic in nature. Therefore, one important design criteria emerges from these observations: strong anisotropy can facilitate flexibility in the COFs while providing high heat conduction channels in a particular direction.

The flexibility of these linkers is further highlighted through stress localization analyses, where Von Mises stress distributions (Figure 4) under 10% strain reveal significant stress concentration at the joints for flexible COFs, especially in Region 5. In contrast, the stiffer linkers of Region 3 show minimal stress localization. COFs in Regions 3 and 6 benefit from better mechanical integrity due to shorter, more rigid linkers and stronger bonding environments, compared to the more flexible COFs in Regions 4 and 5 where the linkers can more readily change the angle between the polymeric chains.

**Figure 4:**
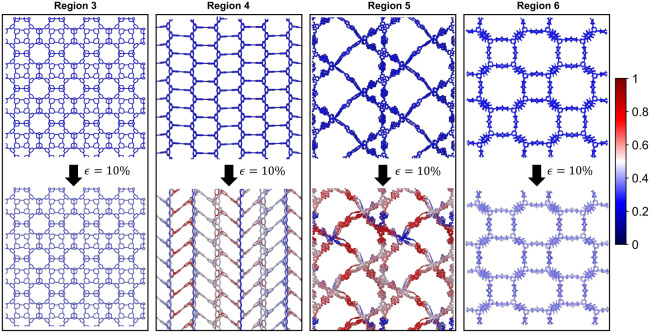
Schematic illustrations of representative 3D COF structures from different regions highlighted in Figure 3g–j, showing volumetric strain under hydrostatic compression. Structures from Regions 3 and 6, which exhibit high bulk modulus, display minimal stress localization (indicated by blue shading). In contrast, structures from Regions 4 and 5, with similar densities but lower bulk moduli, exhibit significant stress localization (indicated by red shading). Additionally, these low-modulus structures undergo pronounced angular deformation of linkers, indicating enhanced flexibility and reduced stiffness. Conversely, high-modulus structures from Regions 3 and 6 retain rigid linker geometries, contributing to their mechanical robustness.

For applications that require both high thermal conductivity and mechanical compliance—such as wearable electronics—Region 4 COFs with flexible and well-aligned chains are most suitable. Notably, recent studies suggest that such flexible COFs can exhibit negative Poisson’s ratios.^91^ As such, combined with the ability to dynamically respond to external stimuli (e.g., strain or guest molecule infiltration), these unique mechanical and thermal properties open up possibilities for use in thermally responsive applications across sectors such as tissue engineering, biomedical devices, aerospace, and defense.^100–103^

To deepen our understanding of the relationship between structure and thermal transport, we performed spectral analyses that captures the acoustic phonon dispersions, frequency-dependent phonon mean free paths and lifetimes by evaluating the dynamical structure factors (details provided in the Methods section). In essence, the dynamical structure factor represents the self-correlation of mass current fluctuations in a thermally equilibrated system—here, at room temperature. These calculations reveal that phonons in the low thermal conductivity regions (Regions 2, 5, and 6) exhibit strong anharmonicity, as indicated by their broad spectral line-widths (see Figure 5a–e). This observation is further supported by our inverse participation ratio (IPR) analysis from lattice dynamics calculations, which shows high IPR values for vibrational modes in these regions (Figure S4), signifying a predominance of localized, nonpropagating vibrations.

**Figure 5:**
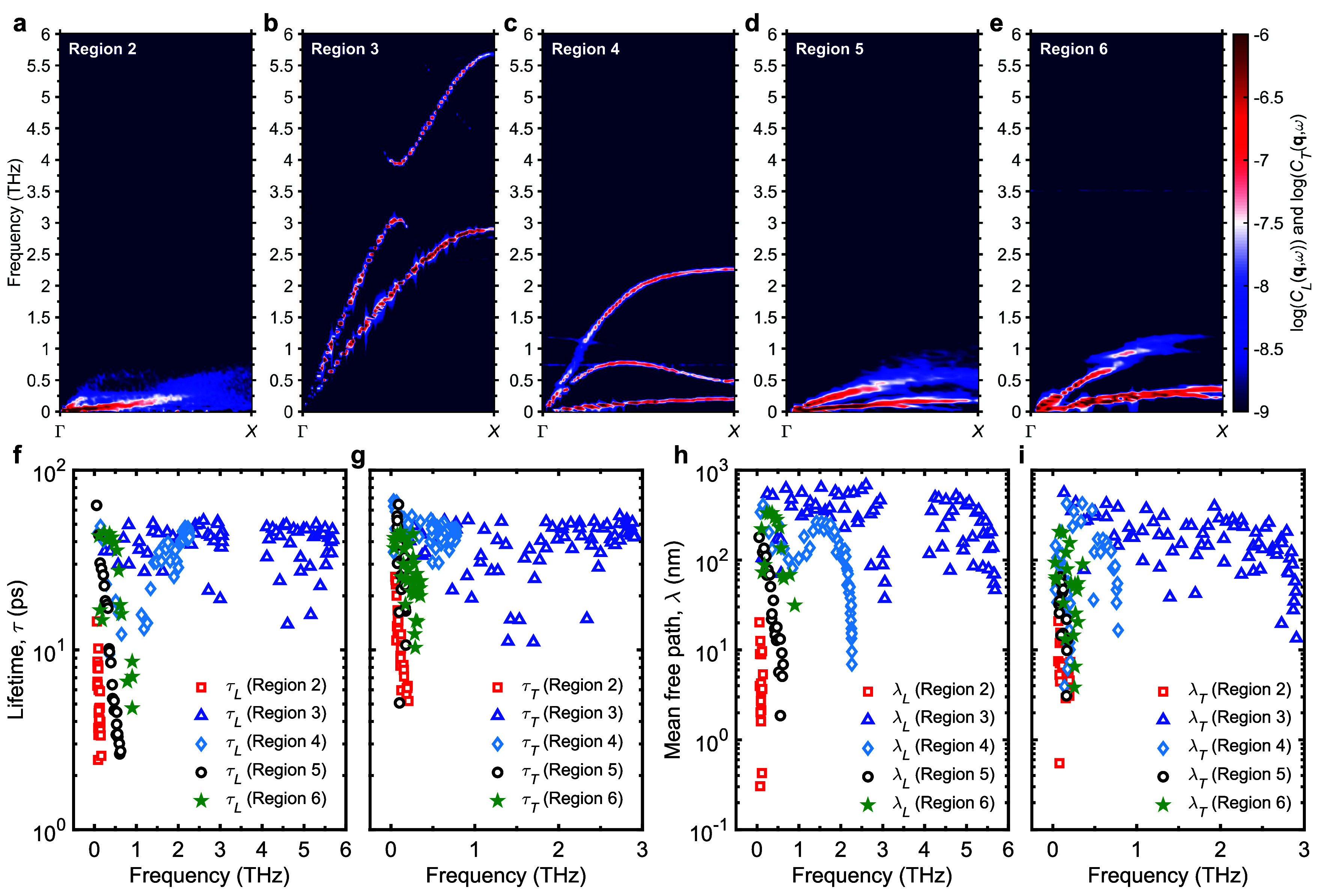
Anharmonic phonon dispersion relations of the longitudinal and transverse current along the Γ-*X* direction for representative 3D COF structures from (a) Region 2, (b) Region 3, (c) Region 4, (d) Region 5, and (e) Region 6. Comparison of (f) longitudinal and (g) transverse acoustic phonon lifetimes of representative 3D COF structures from different regions highlighted in Figure 3f–j. Comparison of (h) longitudinal and (i) transverse acoustic phonon mean free paths of representative 3D COF structures from different regions highlighted in Figure 3f–j.

In these cases, thermal transport is governed primarily by diffusive, nonpropagating vibrational modes known as diffusons,^104–106^ where heat is carried via a random-walk-like motion of localized vibrations, limiting their mean free paths to near atomic dimensions.^107,108^ In contrast, COFs in high thermal conductivity regions (Regions 3 and 4) show sharply defined longitudinal and transverse phonon modes with narrow line-widths, indicative of propagating nature of the phonons (Figure 5b,c). Interestingly, Region 3 COFs support longitudinal phonon modes with frequencies reaching up to 6 THz and high group velocities, exceeding those in Region 4, where acoustic modes only extend to around 2.25 THz and have lower group velocities. These results align with bulk modulus trends, where Region 4 exhibits lower values due to reduced sound speeds, reflecting the influence of more flexible linkers on the phonon group velocities and mechanical stiffness.

Using a damped harmonic oscillator model to fit Lorentzian peaks from the dynamic structure factor data (as detailed in the Methods section), we extract the phonon lifetimes and mean free paths for the 3D COFs. As shown in Figure 5f–i, COFs in Regions 3 and 4 exhibit phonon lifetimes on the order of several tens of picoseconds and mean free paths extending into the hundreds of nanometers. These values are comparable to those observed in high thermal conductivity, fully dense inorganic materials such as GaAs,^109^ and significantly exceed the typical performance of conventional polymers. Ordinarily, phonon mean free paths in polymers are limited due to strong scattering from structural disorder, chain entanglement, and weak interchain forces. Even in semicrystalline polymers, mean free paths are typically confined to less than 100 nm.^110^ Remarkably, the COFs in Regions 3 and 4 achieve mean free paths on par with those found in highly stretched polymer nanofibers, where phonons can travel distances on the order of ∼ 100–1000 nm.^111^

Interestingly, based on their longer phonon lifetimes and higher group velocities of acoustic modes, COFs in Region 3 would be expected to exhibit higher thermal conductivities than those in Region 4. However, given the fact that both regions display similar thermal conductivities, it can be inferred that in Region 4, optical phonons—whose vibrational spectrum extends above 50 THz (see Figure S2), far beyond the acoustic phonon range—make a substantial contribution to heat transport (see Figure S3). Therefore, although the group velocities of acoustic modes are lower than that of Region 3, the significant participation of high-frequency optical modes enhances thermal conductivity in Region 4 COFs beyond what would be expected from acoustic modes alone.

Lastly, we examine how the presence of different atomic species within the functional groups of 3D COFs affects their thermal and mechanical behavior. As illustrated in Figures 6a,b, the incorporation of heavier atoms such as sulfur and silicon tends to lower both thermal conductivity and bulk modulus. In contrast, COFs composed solely of carbon or a combination of carbon and nitrogen exhibit the highest thermal conductivities.

**Figure 6:**
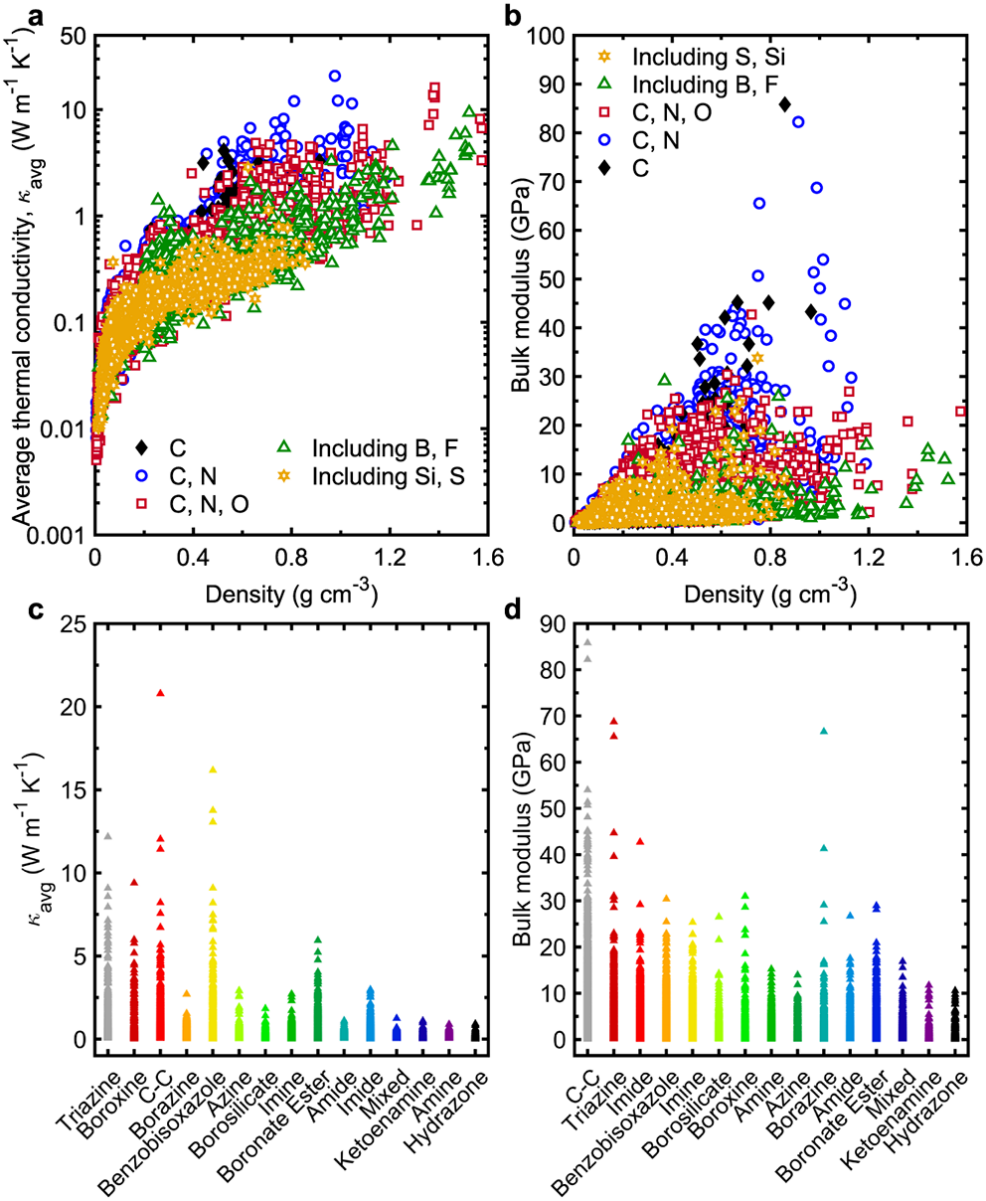
(a) Average thermal conductivity (κ_avg_) and (b) bulk modulus of 3D COFs categorized based on their atom types from both the ReDDCOFFEE and Mercado databases. (c) Distribution of average thermal conductivity (κ_avg_) of 3D COFs categorized based on their bond types from both the ReDDCOFFEE and Mercado databases. (d) Distribution of bulk modulus of 3D COFs categorized based on their bond types from both the ReDDCOFFEE and Mercado databases. Note, bond types are distributed based on their highest to lowest mean values of κ_avg_.

It is also worth mentioning that, while our analysis of 839 COF topologies does not reveal a clear advantage of any single topology in enhancing thermal or mechanical performance, we have classified the COFs by topology and identified the top 20 structures that yield the greatest bulk moduli and thermal conductivities (Figures S8–S10). However, when classifying by functional groups, we observe that COFs containing triazine, boroxine, benzobisoxabole, and carbon–carbon linkages consistently rank among the top performers (Figure 6c,d).

### Thermal and Mechanical Properties of 2D COFs

We now shift our attention to 2D COFs, which have attracted significant interest over the years due to their relatively easier synthesis and crystallization, as well as their high surface areas and accessible one-dimensional pores; these frameworks consist of planar layers stacked through π - π interactions, forming laminar pore structures. For our study, however, we limit our calculations to monolayer configurations, as the UFF-based potential used in our high-throughput screening does not accurately represent the nonbonded π - π interactions and London dispersion forces present in stacked layers.^25,36,40,112^ Nonetheless, examining monolayers offers valuable insight into their in-plane thermal and mechanical properties—which are of primary interest—since the π - π interactions inherently limit both the thermal and mechanical properties of COFs in that weakly bonded direction.^30^

We examined more than 11,000 distinct 2D COF structures, encompassing 104 unique topologies and 698 different linker types. As shown in Figure 7a, while average in-plane thermal conductivity (κ_in‑plane,avg_) generally increases with density, the trend is less pronounced compared to 3D COFs (Figure 1a). On average, 2D COFs exhibit lower thermal conductivities than their 3D analogs. We identified 653 structures with κ_in‑plane,avg_) greater than 1 W m^–1^ K^–1^, including over 23 with values exceeding 2.5 W m^–1^ K^–1^ (Figure 7b and Table S5 for the details of their physical attributes). Note, κ_in‑plane,avg_ represents the average of thermal conductivities along only the bonded in-plane directions for 2D COFs. Notably, the highest thermal conductivities in 2D COFs are concentrated within a density range of 0.6 to 0.9 g cm^–3^, unlike 3D COFs, where higher conductivities typically correlate with densities above 1 g cm^–3^. While previous studies on specific 2D COF with similar topologies have highlighted density as a key factor influencing thermal conductivity,^25,29,30^ our high-throughput screening pinpoints the optimal density window of 0.6 to 0.9 g cm^–3^ for enhancing the in-plane thermal transport in 2D COFs. It is also interesting to note that 2D COFs can possess ultralow thermal conductivities at even moderate to high mass densities. As pointed out below, increasing phonon scattering rates for topologies with multiple pore sizes and misaligned linker chains all contribute to the reduced thermal conductivities.

**Figure 7:**
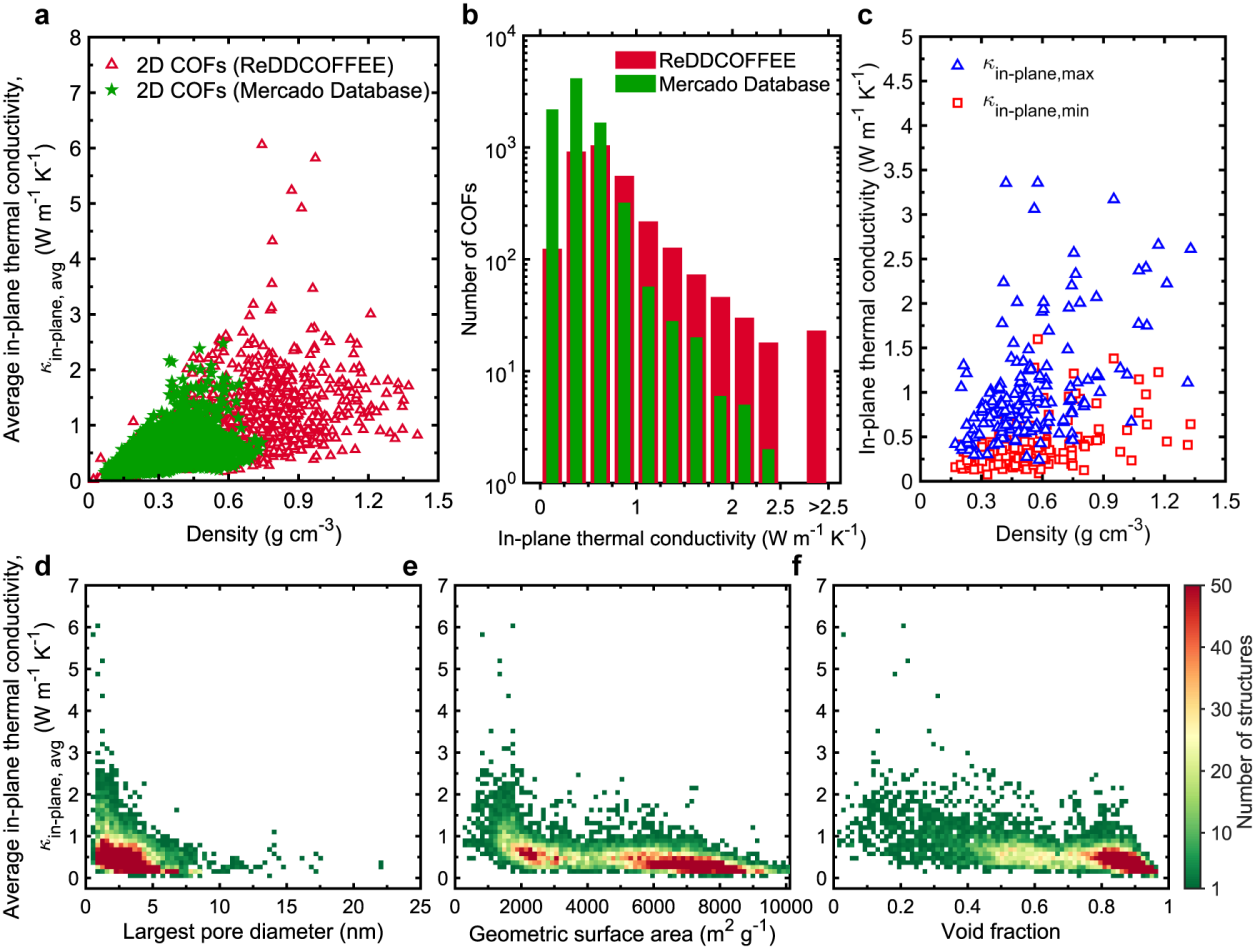
(a) Average in-plane thermal conductivity (κ_in‑plane,avg_) values of 8,455 (Mercado database) and 3,190 (ReDDCOFFEE database) 2D COFs as a function of their density. Overall, 2D COFs from Mercado database exhibit low densities and lower thermal conductivity values compared to 2D COFs from ReDDCOFFEE database. (b) The distribution of κ_in‑plane,avg_ values for 2D COFs from the Mercado and ReDDCOFFEE databases, with a bin size of 0.25 W m^–1^ K^–1^. Notably, we observe that 23 2D COFs from ReDDCOFFEE database only have κ_in‑plane,avg_ values greater than 2.5 W m^–1^ K^–1^. (c) Maximum and minimum in-plane thermal conductivities as a function of density for 163 anisotropic 2D COFs with thermal conductivity anisotropy ratio of ≥ 2. κ_in‑plane,avg_ values of 2D COFs from both the Mercado and ReDDCOFFEE databases as a function of (d) largest pore diameter, (e) geometric surface area, and (f) void fraction, color-mapped according to number of structures discretized into 75 × 75 bins.

These thermal conductivities surpass recent experimental measurements along the bonded direction in 2D COFs,^113^ which may be due to poor stacking order and lower quality of thin-film samples. In contrast, our simulated 2D COF monolayers are idealized and defect-free, leading to higher intrinsic thermal conductivities. In fact, all COF structures investigated in this work are modeled as ideal crystals, without accounting for defects, stacking faults, or other synthesis-induced disorders that could reduce thermal conductivity and mechanical stiffness compared to fully crystalline, defect-free frameworks. In this context, a recent MD study on the HKUST-1 MOF demonstrated that thermal conductivity can be significantly reduced by missing linker defects.^69^ Therefore, the role of defects and structural disorder in governing the thermal properties of COFs warrants further investigation.

Additionally, while 3D COFs display significant anisotropy—with anisotropy ratios reaching up to 50—only a small subset of 2D COFs (Figure 7c) exhibit in-plane anisotropy ratios greater than 2 (163 structures out of 11,645 total). It is worth noting that layered 2D COFs, where sheets are held together by weak van der Waals and π-π interactions, have been reported to exhibit anisotropy ratios as high as ∼4, considering the poor heat conduction along the nonbonded direction.^36^

Notably, 2D COFs with LPDs smaller than 2.5 nm can achieve high thermal conductivities exceeding 2 W m^–1^ K^–1^ (Figure 7d). However, the correlation between thermal conductivity and structural descriptors such as GSA, LPD, and void fraction is less pronounced than in 3D COFs (Figure 7d–f). These findings indicate that heat conduction mechanisms in 2D COFs may differ fundamentally from those in 3D frameworks.

A similar characteristic is also observed for mechanical properties. As shown in Figure 8, the bulk modulus of 2D COFs shows no clear correlation with structural parameters. Surprisingly, the highest bulk moduli are found in structures with densities between 0.3 to 0.7 g cm^–3^—a deviation from the expected behavior where denser frameworks typically exhibit higher stiffness, as seen in 3D COFs. Within this narrow density range, only 10 of the 11,645 2D COFs show a bulk modulus exceeding 50 N m^–1^ (Figure 8b and Table S4 for the details of their physical attributes). Again, no strong dependency on structural descriptors is observed (Figures 8c–e), except that higher bulk moduli tend to occur in COFs with LPDs less than 2.5 nm—consistent with trends seen for thermal conductivity.

**Figure 8:**
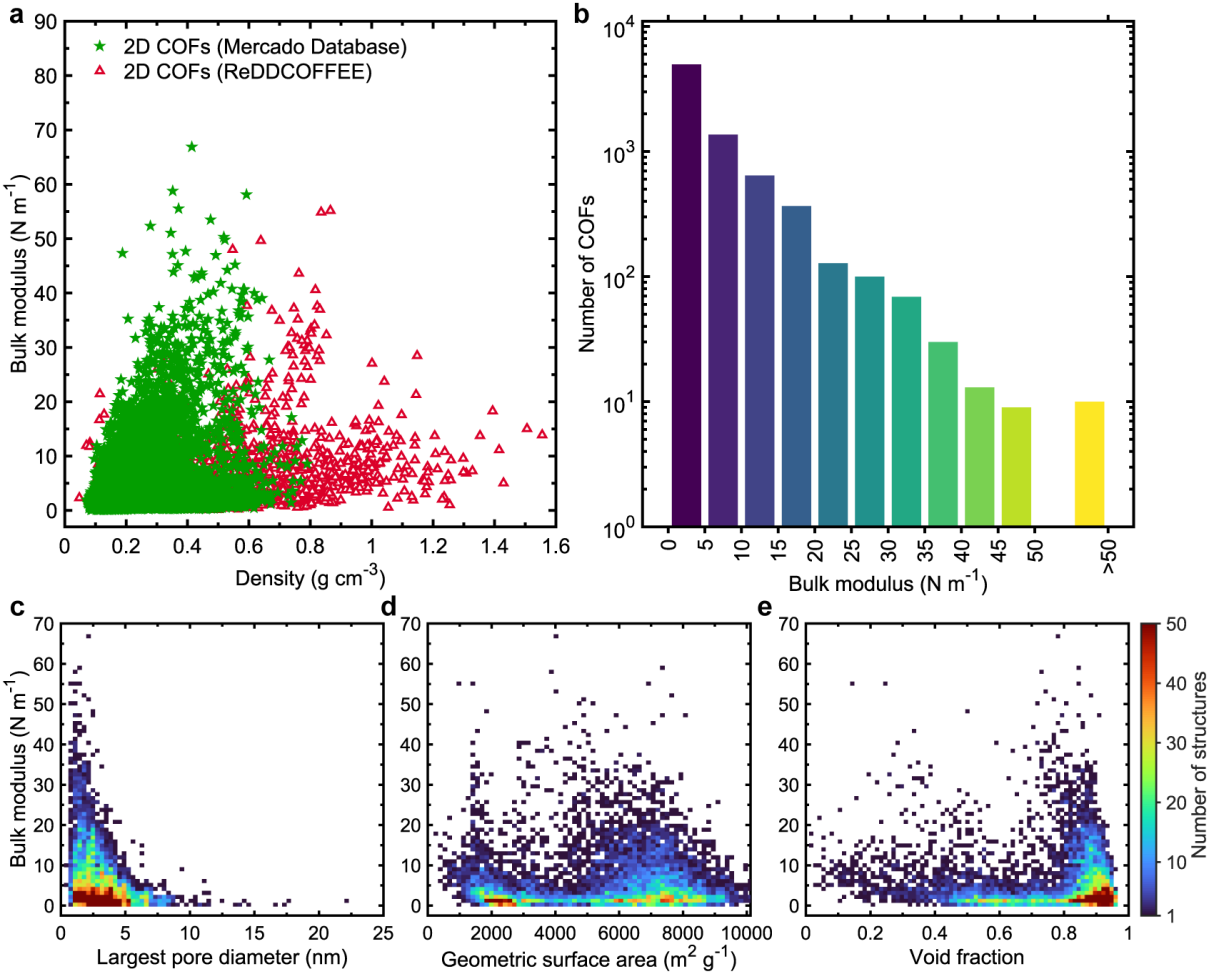
(a) Bulk modulus values of 5,395 (Mercado database) and 2,303 (ReDDCOFFEE database) 2D COFs as a function of their density. Overall, MOFs exhibit higher bulk modulus values compared to COFs. (b) The distribution of bulk modulus values of 2D COFs combining both the ReDDCOFFEE and Mercado databases, with a bin size of 5 N m^–1^. Notably, we observe that 82% of the structures have bulk modulus values below 10 N m^–1^, while 10 structures (combining both the database) exhibit bulk modulus greater than 50 N m^–1^. Bulk modulus of 2D COFs from both the Mercado and ReDDCOFFEE databases as a function of (c) largest pore diameter, (d) geometric surface area, and (e) void fraction, color-mapped according to number of structures discretized into 75 × 75 bins.

To uncover shared structural features that contribute to both high thermal conductivity and bulk modulus in 2D COFs, we plot bulk modulus against thermal conductivity in Figure 9a–d. Interestingly, unlike the trends observed in 3D COFs, there is no strong correlation between these two properties. In fact, the highest bulk moduli are found in COFs with thermal conductivities around 1–2 W m^–1^ K^–1^.

**Figure 9:**
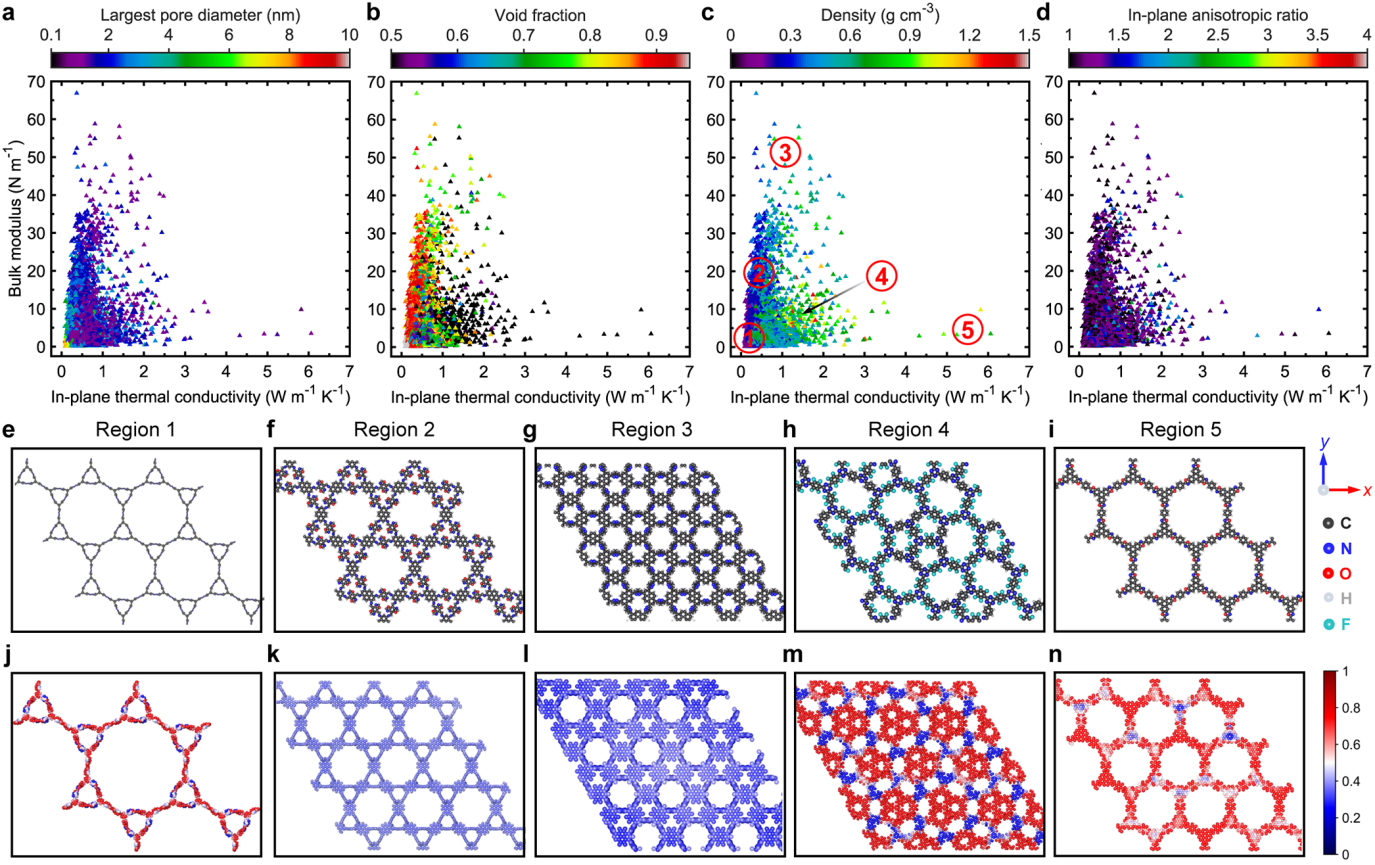
Bulk modulus as a function of average in-plane thermal conductivity (κ_in‑plane,avg_) for 2D COFs, color-mapped by (a) Largest pore diameter (LPD), (b) void fraction, (c) density, and (d) in-plane anisotropic ratio. Representative schematic illustrations of 2D COF structures from various regions highlighted in Figure 9c. (e) Region 1: low bulk modulus and low κ_in‑plane,avg_. (f) Region 2: increased bulk modulus and low κ_in‑plane,avg_. (g) Region 3: high bulk modulus and high κ_in‑plane,avg_. (h) Region 4: low κ_in‑plane,avg_ with moderate bulk modulus. (i) Region 5: low bulk modulus and high κ_in‑plane,avg_ with density comparable to Region 4. (j-n) Volumetric strain maps under hydrostatic compression corresponding to each region, relative to the relaxed structures. Pronounced stress localization (indicated by red shading) is observed in Regions 1, 4, and 5, which are associated with lower bulk modulus values.

Figure 9c highlights several regions in the 2D COF property space that merit closer examination. Regions 1 and 2 exhibit both low thermal conductivity and bulk modulus, which can be attributed to their high void fractions and low overall densities. Region 3 includes COFs with relatively high bulk moduli and moderate thermal conductivities. Here, heat transport is limited by phonon scattering from pores of varying pore sizes (see Figure 9e–g for representative examples). The high stiffness of Region 3 COFs is due to the presence of bulky functional groups, which also prevent stress localization even at 10% strain (Figure 9l).

In contrast, Region 4 COFs—despite having similar densities and thermal conductivities to Region 3—exhibit lower bulk moduli. This is due to their smaller functional groups, which lead to greater stress localization under deformation (Figure 9m). Region 5 COFs are particularly noteworthy: they show the highest thermal conductivities among all 2D COFs, yet have very low bulk moduli. This behavior arises from highly aligned polymer chains—particularly those incorporating HHTP linkers—which enhance heat conduction along the polymer backbone.

Further insight is provided by dynamic structure factor calculations for 2D COFs in each of the five regions (Figure 10a–e). Regions 1 and 2, with ultralow thermal conductivities, lack well-defined high group velocity acoustic modes. In contrast, Region 5 COFs—the top performers in thermal transport—display pronounced longitudinal and transverse acoustic branches with high group velocities, even exceeding those in Region 3, which have the highest bulk moduli. This finding is somewhat counterintuitive, as higher bulk modulus typically correlates with faster sound propagation. However, this trend does not hold for 2D COFs in our data set, since the linker flexibility plays a pivotal role in controlling the mechanical stiffness. Also, 2D COFs from Region 5 exhibit some acoustic phonon modes with mean free paths and lifetimes (Figure 10f–i) that are comparable to those of high-performance 3D COFs, further explaining their superior thermal conductivity among the COFs from other regions for the 2D COFs.

**Figure 10:**
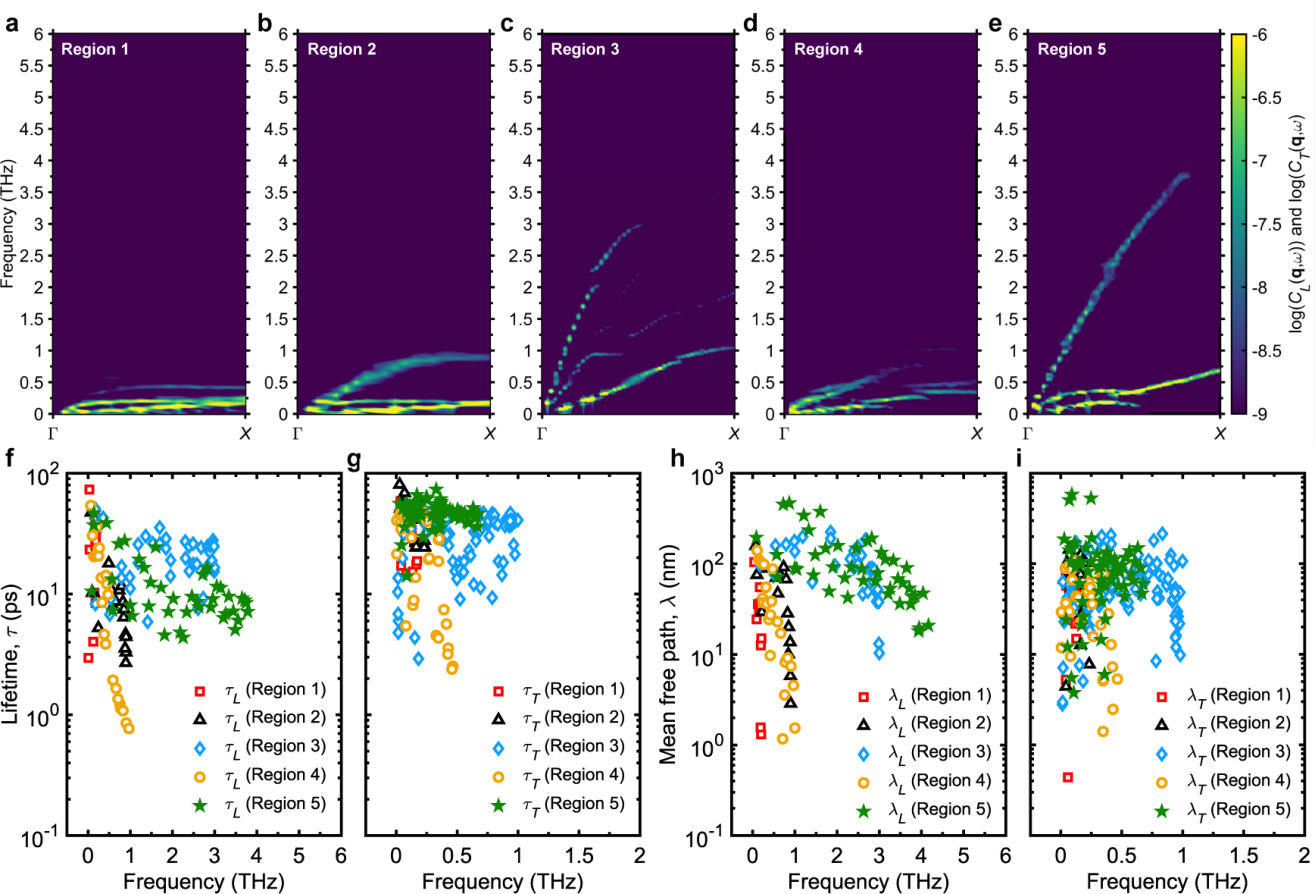
Anharmonic phonon dispersion relations of the longitudinal and transverse current along the Γ-*X* direction for representative 2D COF structures from (a) Region 1, (b) Region 2, (c) Region 3, (d) Region 4, and (e) Region 5. Comparison of (f) longitudinal and (g) transverse acoustic phonon lifetimes of representative 2D COF structures from different regions highlighted in Figure 9e–i. Comparison of (h) longitudinal and (i) transverse acoustic phonon mean free paths of representative 2D COF structures from different regions highlighted in Figure 9e–i.

In contrast to 3D COFs—where no single topology consistently yields superior thermal and mechanical performance—we identify four specific 2D COF topologies (**kgm**, **hcb**, **sql**, and **bex**) that stand out for their multifunctionality (Figure 11a,b). These frameworks exhibit both bulk moduli exceeding 20 N m^–1^ and thermal conductivities above 1 W m^–1^ K^–1^, marking them as promising candidates for applications requiring mechanical robustness and efficient heat transport (see representative structures in Figures S30 and Table S6 for the details of their physical attributes). When evaluating performance by functional group, high-performing 2D COFs mirror trends seen in 3D structures: linkages incorporating triazine, boroxine, benzobisoxazole, and carbon–carbon bonds consistently enable enhanced properties (Figures 11c,d). This reinforces the idea that certain chemical motifs confer advantageous behavior across dimensions.

**Figure 11:**
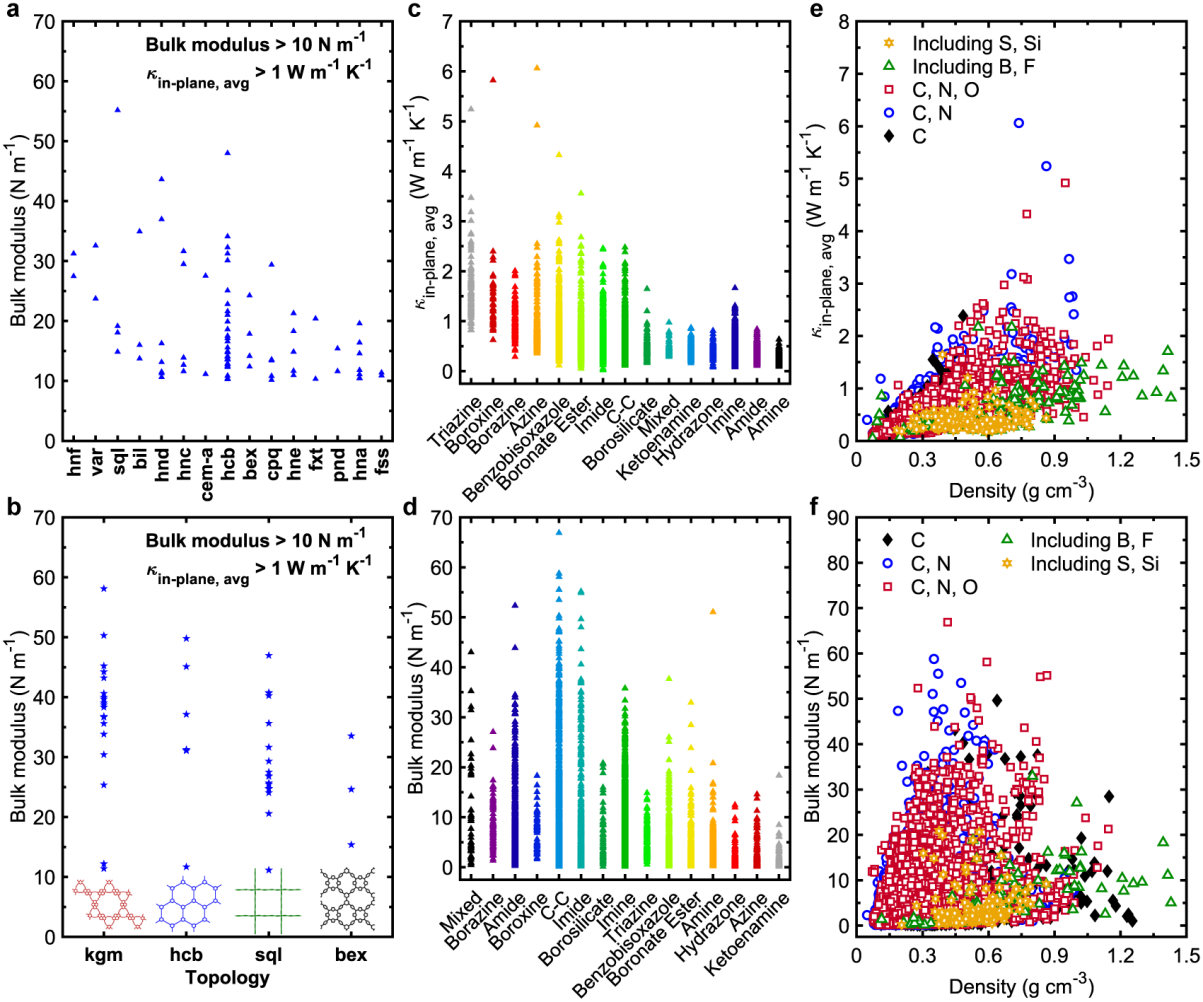
Distribution of the bulk modulus (BM) of 2D COFs for the top topologies with BM > 10 N m^–1^ and κ_in‑plane,avg_ > 1 W m^–1^ K^–1^ from the (a) ReDDCOFFEE and (b) Mercado databases. (c) Distribution of average thermal conductivity (κ_avg_) of 2D COFs categorized based on their bond types from both the ReDDCOFFEE and Mercado databases. (d) Distribution of bulk modulus of 2D COFs categorized based on their bond types from both the ReDDCOFFEE and Mercado databases. Note, bond types are distributed based on their highest to lowest mean values of κ_avg_. (e) Average in-plane thermal conductivity (κ_in‑plane,avg_) and (f) bulk modulus of 2D COFs categorized based on their atom types from both the ReDDCOFFEE and Mercado databases.

We have also identified several topologies exhibiting thermal conductivities below 0.4 W m^–1^ K^–1^, yet maintaining moderate to high mass densities (≳ 0.5 g cm^–3^). Examples include **fsz-a**, **ply**, **fss**, **fxt**, **tts-a**, **tth-a**, and **htb-a**, where pores are largely misaligned and span three distinct sizes—features that collectively enhance phonon scattering and promote stronger localization of vibrational modes (see Figures S25 and S28). Notably, in addition to pore misalignment, the relative orientation of the polymer chains with respect to the *xy*-plane (containing the COF monolayers) plays a crucial role. For instance, the **kgm** topology with flat linkers aligned along the heat transport direction exhibits high thermal conductivity (Figure S29a), whereas a **kgm** structure of the same density but with linkers misaligned relative to the *xy*-plane displays ultralow thermal conductivity (Figure S29b).

Additionally, as in 3D COFs, incorporating heavier elements such as sulfur and silicon tends to suppress thermal conductivity in the 2D systems as well (Figures 11e,f). However, a key distinction emerges: while structural attributes like density, LPD, and void fraction are primary performance drivers in 3D COFs, they appear to play a less dominant role in 2D analogs. In 2D COFs, the specific topology and chemical composition, particularly the selected linkage groups, play a decisive role in governing thermal and mechanical properties. For thermal conductivity in particular, the orientation of these linkages relative to the *xy*-plane emerges as a key determining factor. These findings underscore the importance of targeted chemical and topological design in 2D COFs to achieve high-performance multifunctional materials.

## Conclusion

Structure–property relationships in 3D COFs reveal that thermal conductivity and bulk modulus are governed by distinct yet overlapping structural features. High thermal conductivity can be achieved in both rigid and flexible COFs via anisotropic phonon transport, while mechanical stiffness correlates more strongly with linker rigidity and atomic connectivity. Design principles are established for tuning COFs toward multifunctional applications.

Our comprehensive analysis reveals that while the bulk modulus of 3D COFs loosely correlates with mass density, it exhibits far greater variability compared to thermal conductivity, highlighting the more complex and nuanced structural origins of mechanical stiffness. Although most COFs remain mechanically soft, a surprising subset achieves bulk moduli exceeding 50 GPa—comparable to ceramics—underscoring the potential for mechanical robustness in these polymeric frameworks. Structural parameters such as pore size, surface area, and void fraction, which are strongly predictive of thermal conductivity, prove to be poor indicators of stiffness. Instead, factors like polymer chain alignment, linker rigidity, and bonding strength emerge as critical determinants.

By mapping bulk modulus and thermal conductivity together, we identify distinct structure–property regions that elucidate the trade-offs and synergies between thermal and mechanical performance. In particular, COFs with stiff, well-aligned linkers achieve high values in both properties, while flexible COFs can still support substantial thermal transport through anisotropic phonon propagation and optical mode contributions. Spectral analyses confirm that high-performing COFs exhibit long-lived phonon modes (with lifetimes of several tens of picoseconds) and long mean free paths (extending to several hundred nanometers), rivaling those in some fully dense high thermal conductivity inorganic semiconductors such as GaAs, GaN, SiC, Mg_2_Si, and Mg_2_Sn.^114–116^

Finally, our results show that chemical composition and linker functionality significantly influence mechanical and thermal behavior, with light-atom linkages (e.g., C, N) and rigid motifs (e.g., triazine, boroxine) consistently associated with superior performance regardless of the dimensionality. These insights not only deepen the fundamental understanding of structure–property relationships in COFs but also offer practical design rules for tailoring these materials for multifunctional applications requiring both superior thermal conductivity and mechanical resilience.

## Methods

For the high-throughput screening study, we utilized COF structures obtained from the Materials Cloud platform, comprising two sources: the Mercado database, which contains 8,641 2D and 61,199 3D COFs,^98^ and the ReDDCOFFEE database, which includes 5,856 2D and 262,822 3D COFs.^117^ From these databases, we curated a diverse subset of 21,000 3D COFs from the ReDDCOFFEE database and 12,000 3D COFs from the Mercado database, ensuring diversity in topology, linker chemistry, and bond types. For the 2D COFs, due to their relatively smaller numbers, we included all available structures from both databases, resulting in a total of 14,497 2D COFs.

Thermal conductivity and bulk modulus calculations are carried out for each structure, as detailed in the subsequent sections. Out of the total 33,000 selected 3D COFs and 14,497 2D COFs, we successfully obtained converged thermal conductivity values for 26,700 3D and 11,645 2D structures. Similarly, bulk modulus values are successfully calculated for 21,425 3D and 7,698 2D COFs.

For MD simulations, we employed the Large-scale Atomic Molecular Massively Parallel Simulator (LAMMPS) package.^118^ Interatomic interactions are described using the Universal Force Field (UFF), adapted specifically for porous organic materials.^119,120^ This force field accounts for bonded interactions, including bond, angle, dihedral, and torsional terms, while nonbonded interactions are computed with a cutoff distance of 12.5 Å. Electrostatic interactions are not included in our calculations due to the high computational cost.

### High-Throughput Thermal Conductivity Calculations

To evaluate thermal conductivity across a large set of 2D and 3D COFs, we performed equilibrium molecular dynamics (EMD) simulations using the Green–Kubo (GK) formalism. Each structure is initially equilibrated in the canonical (NVT) ensemble for 1.5 ns using a time step of 0.5 fs, during which the number of particles, temperature, and simulation volume are held constant. This is followed by an additional 1 ns equilibration under the microcanonical (NVE) ensemble, conserving the total energy of the system. Periodic boundary conditions are applied in all spatial directions throughout the simulations.

The GK method is then used to calculate thermal conductivity by integrating the heat current autocorrelation function (HCACF). The direction-resolved thermal conductivity, κ_α_, is defined as

1 $$\kappa_{\alpha }=\frac{1}{k_{B}VT^{2}}\int_{0}^{\infty }\left\langle J_{\alpha }\left(t\right)\cdot J_{\alpha }\left(0\right)\right\rangle dt$$

where *k*_B_ is the Boltzmann constant, *T* is the system temperature, *V* is the simulation cell volume, and *J*_α_(*t*) is the α^th^ component of the heat current. The heat current vector is computed as^121^

2 $$J=\frac{1}{V}\left(\underset{i}{\sum }v_{i}\epsilon_{i}+\underset{i}{\sum }S_{i}\cdot v_{i}\right)$$

where *v*_*i*_, ϵ_*i*_, and S_*i*_ denote the velocity, per-atom energy, and per-atom stress tensor of atom *i*, respectively. The heat current is sampled every 7 fs over the production run of 4.5 ns in the NVE ensemble.

To ensure accuracy in HCACF integration, we employed variable correlation times (ranging from 5 to 75 ps) depending on the density of the COFs. Low-density structures generally required shorter correlation windows, whereas high-density systems necessitated longer time intervals for proper convergence. We also verified size convergence by performing additional simulations on selected COFs at different densities with varying domain sizes, confirming that dimensions exceeding 50 Å in each direction are sufficient to eliminate size-effects.^122^

It should be noted that this study does not account for electronic contributions to thermal conductivity, since COFs are generally wide-bandgap materials where heat transport is primarily governed by lattice vibrations rather than charge carriers.^123,124^ Nonetheless, recent reports indicate that certain 2D COFs can exhibit notable electronic conductivities,^125–128^ which are not considered here.

For a subset of structures, persistent oscillations or nondecaying instantaneous thermal conductivity values made it difficult to extract converged results. To address this, we implemented an adaptive algorithm to identify plateau regions by analyzing the slope, mean, and standard deviation of instantaneous thermal conductivity over rolling time windows.^97^ Specifically, for correlation times ≤ 15 ps, we used 3 ps segments with 2 ps increments; for longer total correlation times, 15 ps segments with 5 ps increments are employed. A segment is considered valid if its standard deviation is less than 20% of the segment mean and the normalized slope is below 0.01 ps^–1^. If multiple segments satisfied these conditions, the one with the lowest standard deviation is selected; in the case of equal standard deviations, the segment with the smaller slope is prioritized. Structures that did not meet these convergence criteria are excluded or rerun with extended correlation times. This automated workflow enabled efficient and reliable extraction of thermal conductivity values across tens of thousands of COF structures.

### High-Throughput Bulk Modulus Calculations

To evaluate the mechanical stiffness of COFs, we computed the bulk modulus through energy-volume (for 3D COFs) or energy-area (for 2D COFs) analysis using a multistep workflow. All simulations are performed using LAMMPS,^118^ where energy minimizations and volume perturbations are applied to probe mechanical response. For 3D COFs, each structure is first subjected to energy minimization using a two-stage protocol combining the conjugate gradient (CG)^129^ and fast inertial relaxation engine (FIRE)^130^ algorithms to ensure convergence of potential energy landscape. Cell relaxation is performed with an anisotropic stress condition and energy tolerance thresholds set to 10^–10^ kcal mol^–1^. Following minimization, we performed an NPT-based volume perturbation sequence to generate an energy-volume (*E*-*V*) curve. The system is initially equilibrated at 1 K using the Nosé-Hoover barostat^131^ with an initial pressure in the range of −0.03 to −0.1 GPa, depending on the density of the COFs. After equilibration, we gradually increased the pressure (ranging from 0.1 to 1 GPa) depending on the density of the COFs to induce compression. The energy and corresponding volume are recorded for 2 ns to establish the energy-volume (*E*-*V*) curve. A stopping criterion is applied based on a 10% change in volume to avoid excessive distortion of the framework.

For 2D COFs, the bulk modulus can be redefined according to the change in energy as the relative change of area instead of volume. After energy minimization, the structures are equilibrated at 1 K, and pressure is applied only in the in-plane directions, while keeping the cross-plane dimension fixed. A gradual increase in lateral pressure is applied to induce areal compression, and the resulting energy-area (*E*-*A*) data are collected for postprocessing.

For both 2D and 3D COFs, the raw deformation data are postprocessed using an adaptive fitting algorithm designed to automatically identify the most physically meaningful region of the curve for bulk modulus extraction. The adaptive algorithm begins by checking whether the full data set produces a high-quality fit to the Murnaghan equation of state (EOS) (i.e., *R* ^2^ ≥ 0.9995).^132,133^ If this criterion is not met, the algorithm iteratively scans through data segments using a sliding window approach starting with segments of at least 10 points and expanding dynamically. Each candidate range is evaluated using a composite score that combines the *R* ^2^ of a polynomial fit with the curvature magnitude, and the range with the highest score is selected for EOS fitting. Once the optimal range is identified, we fit the selected region to three widely used EOS models: Murnaghan EOS,^132,133^ Birch–Murnaghan EOS,^134^ and Vinet EOS.^135^ For each model, a nonlinear least-squares minimization is performed to extract the bulk modulus. We compute the *R* ^2^ goodness-of-fit metric for each model, and only accept fits where *R* ^2^ ≥ 0.9. The final reported bulk modulus is the average of the valid values obtained from the three EOS fits. Structures that fail to yield a valid fit under all models are excluded from the data set. The entire process is automated to enable robust and scalable evaluation of mechanical properties across tens of thousands of 2D and 3D COFs.

### Dynamical Structure Factor Calculations

The vibrational spectra of representative 2D and 3D COFs are evaluated from MD trajectories using the DYNASOR package.^136^ In this approach, the longitudinal and transverse current correlation functions are computed from atomic velocities and positions, fully incorporating for anharmonic effects. These are expressed as,^136^

3 $$C_{L}\left(q,\omega \right)=\int_{-\infty }^{\infty }\frac{1}{N}\left\langle j_{L}\left(q,t\right).j_{L}\left(-q,0\right)\right\rangle e^{-i\omega t}dt$$

4 $$C_{T}\left(q,\omega \right)=\int_{-\infty }^{\infty }\frac{1}{N}\left\langle j_{T}\left(q,t\right).j_{T}\left(-q,0\right)\right\rangle e^{-i\omega t}dt$$

Here, N is the number of atoms, **q** is the wave vector, and *j*_*L*_(**q**,*t*) and *j*_*T*_(**q**,*t*) are the longitudinal and transverse current densities, given by

5 $$j_{L}\left(q,t\right)=\overset{N}{\underset{i}{\sum }}\left(v_{i}\left(t\right)\cdot \mathrm{q̂}\right)\mathrm{q̂}e^{iq\cdot r_{i}(t)}$$

6 $$j_{T}\left(q,t\right)=\overset{N}{\underset{i}{\sum }}\left[v_{i}\left(t\right)-\left(v_{i}\left(t\right)\cdot \mathrm{q̂}\right)\mathrm{q̂}\right]e^{iq\cdot r_{i}(t)}$$

Here, **q̂** is the unit vector along **q**, and *v*_*i*_(*t*) and **r**_**i**_(*t*) correspond to the velocity and position of atom *i* at time *t*, respectively.

For spectral calculations, larger computational domain are constructed from the unit cells to ensure sufficient **q**-point resolution. Initially, the supercell is equilibrated under the same conditions as the EMD simulations, followed by NVE production runs of 1.5 ns, during which velocities and positions are sampled at regular intervals for subsequent spectral analysis.

### Damped Harmonic Oscillators—Fitting

The current correlation functions, *C*_*L*_(**q**,ω) and *C*_*T*_(**q**,ω) are analyzed by fitting to a damped harmonic oscillator (DHO) model to extract phonon frequencies and lifetimes.^136–138^ The DHO equation of motion is

7 $$ẍ\left(t\right)+\Gamma ẋ\left(t\right)+\omega_{0}^{2}x\left(t\right)=0$$

Here, ω_0_ is the natural frequency and Γ is the damping constant. Fourier transforming this equation yields the power spectral density expressed as

8 $$x\left(\omega \right)=A\frac{2\Gamma \omega_{0}^{2}}{{\left(\omega^{2}-\omega_{0}^{2}\right)}^{2}+{\left(\Gamma \omega \right)}^{2}}$$

This yields three fitting parameters *A*, Γ, and ω_0_ for each **q**. For current correlation functions, the DHO form can be extended to,

9 $$C_{L,T}\left(q,\omega \right)=A\frac{2\Gamma \omega_{0}^{2}\omega^{2}}{{\left(\omega^{2}-\omega_{0}^{2}\right)}^{2}+{\left(\Gamma \omega \right)}^{2}}$$

From these fits, the phonon lifetime (τ) is obtained as τ = 2/Γ, and the group velocity (*v*_*L, T*_) from the slope of the dispersion relation ω_*L, T*_(**q**). The corresponding mean free path (λ_*L,T*_) is then computed as λ_*L,T*_ = *v*_*L,T*_ × τ.

## Supplementary Material



## Data Availability

The data supporting the present work are available from the corresponding authors upon reasonable request.

## Notes

Additional methodological details, including spectral heat flux calculations, inverse participation ratio; additional results and details of the physical attributes for the top- and worst-performing COFs (PDF)
