# Supplementary material for: Guiding the Design of Multifunctional Covalent Organic Frameworks: High-Throughput Screening of Thermal and Mechanical Properties
Source: Chem Mater. 2025 Oct 21;37(21):8978–95. doi: 10.1021/acs.chemmater.5c02310 (PMC12613323; doi:10.1021/acs.chemmater.5c02310)
Supplement: Supplementary file 1 [file cm5c02310_si_001.pdf]

Supporting Information:

Guiding the Design of Multifunctional Covalent Organic Frameworks: High-Throughput Screening of Thermal and Mechanical Properties

Sandip Thakur[1*] and Ashutosh Giri[1*]

[1]Department of Mechanical, Industrial, and Systems Engineering, University of Rhode Island, Kingston, 02881, RI, USA.

*Corresponding author(s). E-mail(s): sandip_thakur@uri.edu; ashgiri@uri.edu;

Contents

I. Supplementary Methods **S2**

A. Spectral Heat Flux Calculation S2

B. Inverse Participation Ratio (IPR) S2

II. 3D COFs **S3**

Distribution of unique building blocks of 3D COFs selected for our high-throughput calculations S3

A. **Supplementary Figures** S3

Maximum directional thermal conductivity vs density of 3D COFs S3

Vibrational density of states comparison of 3D COFs S4

Spectral heat flux comparison of 3D COFs (regions 3 and 4) S4

Comparison of inverse participation ratio (IPR) of 3D COFs S4

Relationships between bulk modulus and average thermal conductivity, color-mapped by GSA S4

Schematic of low-anisotropy 3D COFs from region 3 . . . . . S4

Schematic of high-anisotropy 3D COFs from region 4 . . . . . S4

Top and worst 3D COF topologies for thermal transport . . . S4

Top and worst 3D COF topologies for bulk modulus . . . S4

Top and worst 3D COF topologies for bulk modulus and thermal conductivity . . . S4

Schematics of high bulk modulus ($>$ 50 GPa) 3D COF structures . . . S10

Schematics of high thermal conductivity 3D COF structures with $\kappa_{\text{avg}} \geq$ 10 W m^{-1} K^{-1} . . . S13

B. **Supplementary Tables** . . . S15

Properties of 56 3D COFs with high bulk modulus ($\gtrsim$ 30 GPa) . . . S15

Properties of 136 3D COFs with high thermal conductivities ($\gtrsim$5 W m^{-1} K^{-1}) . . . S17

Properties of 46 3D COFs with bulk modulus greater than 30 GPa and $\kappa_{\text{avg}} >$ 1 W m^{-1} K^{-1} . . . S21

III. 2D COFs **S23**

Distribution of unique building blocks of 2D COFs selected for our high-throughput calculations . . . S23

A. **Supplementary Figures** . . . S23

Comparison of inverse participation ratio (IPR) of 2D COFs . . . S23

Top and worst 2D COF topologies for thermal transport . . . S23

Top and worst 2D COF topologies for bulk modulus . . . S23

Schematic illustrations of topologies with misaligned pores and distinct sizes . . . S26

Schematic illustrations of **kgm** topology for 2D COFs with aligned and misaligned linkers . . . S26

Schematics of 2D COFs with high bulk modulus ($>$ 20 N m^{-1}) and $\kappa_{\text{in-plane,avg}} >$ 1 W m^{-1} K^{-1} . . . S26

Schematics of high bulk modulus ($\gtrsim$ 50 N m^{-1}) 2D COF structures . . . S27

Schematics of high $\kappa_{\text{in-plane,avg}}$ ($\gtrsim$ 3 W m^{-1} K^{-1}) 2D COF structures . . . S28

B. **Supplementary Tables** . . . S29

Properties of 131 2D COFs with high bulk modulus ($\gtrsim$ 30 N m^{-1}) . . . S29

Properties of 78 2D COFs with high average in-plane thermal conductivity ($\gtrsim$ 2 W m^{-1} K^{-1}) . . . S33

Properties of 66 2D COFs with bulk modulus greater than 20 N m^{-1} and $\kappa_{\text{in-plane,avg}} \gtrsim$ 1 W m^{-1} K^{-1} S36

I. Supplementary Methods

A. Spectral Heat Flux Calculation

To determine the contribution of different vibrational frequencies to the overall heat flux, the spectral heat flux of COFs is calculated as,[S1, S2]

$$Q = \int_0^\infty \frac{d\omega}{2\pi} q(\omega) \tag{1}$$

where ω represents the angular frequency, and $q(\omega)$ is the spectral heat current, defined by

$$q_{i\to j}(\omega) \propto \langle \vec{F}_{i,j} \cdot (\vec{v}_i + \vec{v}_j) \rangle \tag{2}$$

Here, $q_{i\to j}(\omega)$ captures the correlation between the interatomic force $\vec{F}_{i,j}$ and the velocities of atoms i and j ($\vec{v}_i$ and $\vec{v}_j$). Atomic forces and velocities are sampled at intervals of 5 fs over a total simulation time of 1 ns to compute the spectral heat flux.

B. Inverse Participation Ratio (IPR)

Harmonic lattice dynamics calculations are performed to evaluate the inverse participation ratio (IPR) of phonon modes in our systems using the General Utility Lattice Program (GULP).[S3] The IPR is defined as:[S4, S5]

$$\mathrm{IPR} = \frac{\sum_i^N \left(\sum_{\alpha=1}^3 u_{i\alpha}^2 \right)^2}{\left(\sum_{i=1}^N \left(\sum_{\alpha=1}^3 u_{i\alpha}^2 \right) \right)^2} \tag{3}$$

where N is the total number of atoms and $u_{i\alpha}$ is the eigenvector component of atom i along direction α. A mode is considered localized if its eigenvector is distributed over less than 20% of the total atoms, providing a practical distinction between localized and delocalized phonon modes.[S4, S5]

II. 3D COFs

Table S1: Distribution of unique building blocks of 3D COFs selected for our high-throughput calculations.

Numbers of unique	ReDDCOFFEE database	Mercado database
Topology	839	234
Linkers	273	651
Bond type	11	5

A. Supplementary Figures

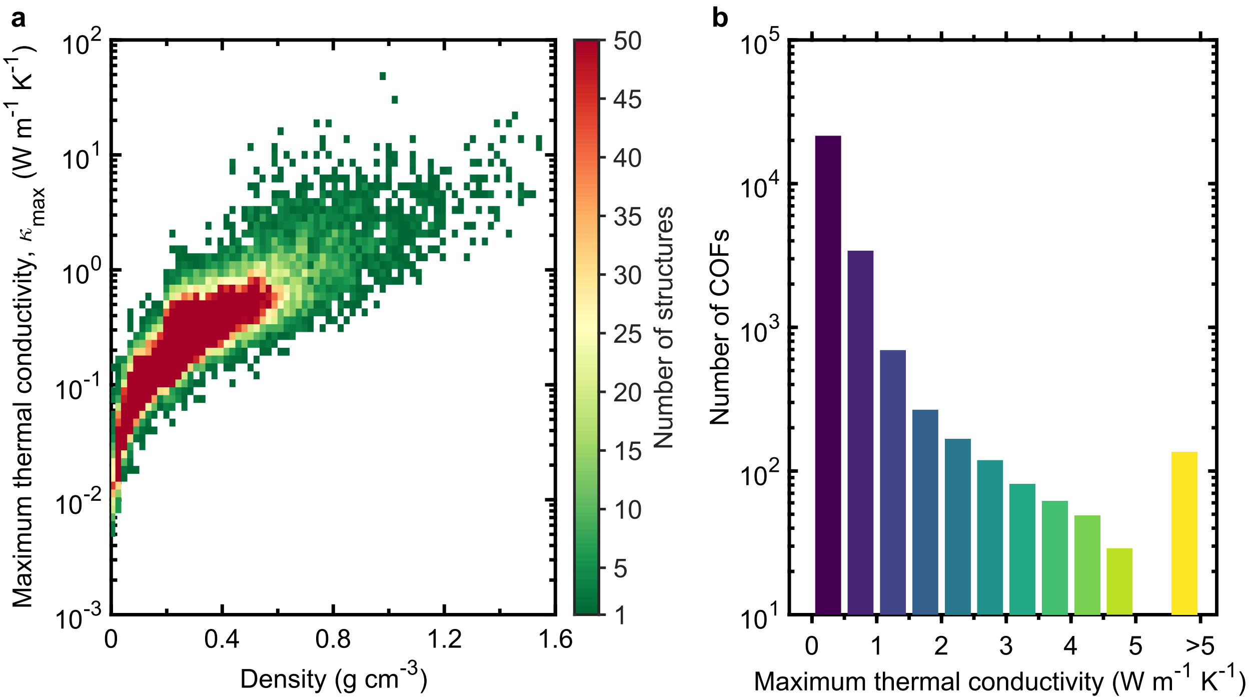

Fig. S1: (a) Maximum directional thermal conductivity, κ_{max}, of 3D COFs as a function of density. (b) The distribution of κ_{max} for the total 26,700 3D COFs from the combined Mercado and ReDDCOFFEE databases (bin size: 0.5 W m^{-1} K^{-1}). A total of 136 structures exhibit κ_{max} exceeding 5 W m^{-1} K^{-1}.

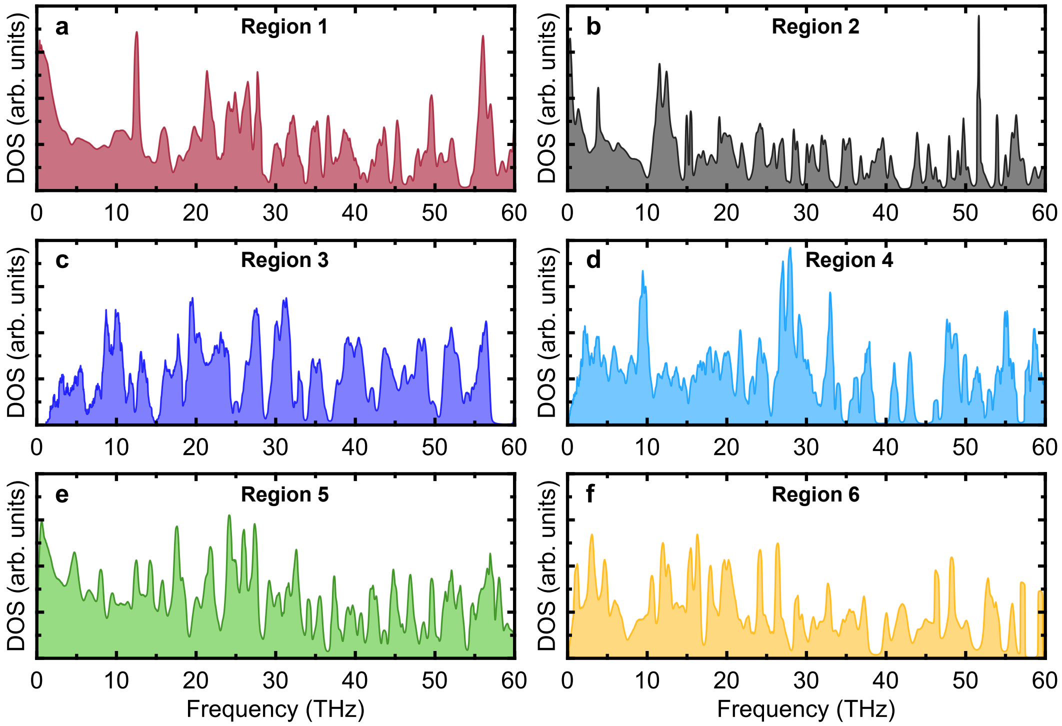

Fig. S2: Comparison of the vibrational density of states for 3D COFs from the different regions highlighted in Figs. 3 of the main text.

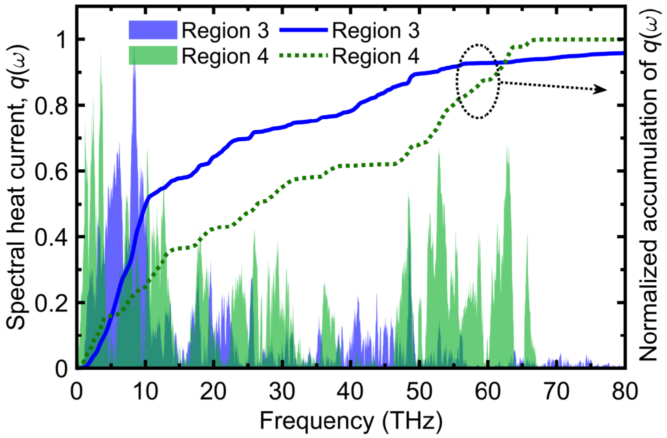

Fig. S3: Comparison of spectral heat flux, $q(\omega)$, for 3D COFs from regions 3 and 4 highlighted in Fig. 3 of the main text. COFs in region 4 exhibit a substantially larger contribution from high-frequency optical vibrations compared to those in region 3.

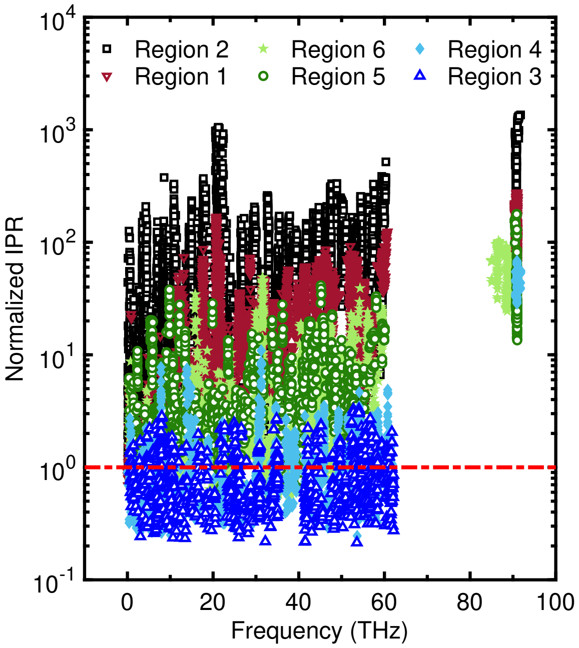

Fig. S4: Comparison of the inverse participation ratio (IPR) of 3D COFs from the regions highlighted in Fig. 3 of the main text. Structures in regions 1 and 2, which exhibit low average thermal conductivity ($\kappa_{\rm avg}$), show higher IPR values across a broad frequency range, indicating strong phonon localization and suppressed vibrational transport. In contrast, COFs from regions 3 and 4 exhibit lower IPR values, suggesting more delocalized phonon modes that facilitate heat transfer, resulting in higher $\kappa_{\rm avg}$. The red dashed line indicates the threshold for localized modes, defined as eigenvectors distributed over less than 20% of the total atoms.[S4, S5].

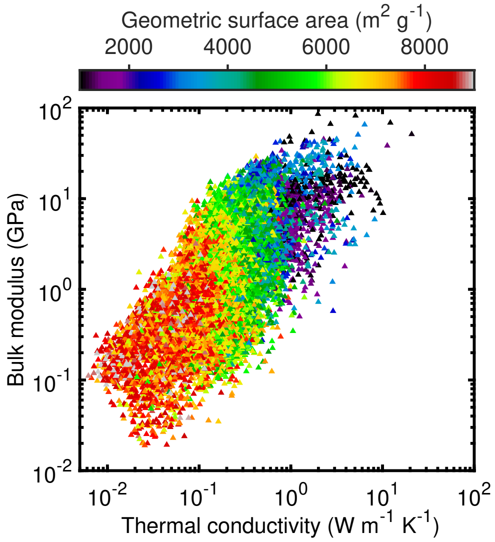

Fig. S5: Bulk modulus as a function of average thermal conductivity values of 3D COFs, color-mapped by geometric surface area (GSA).

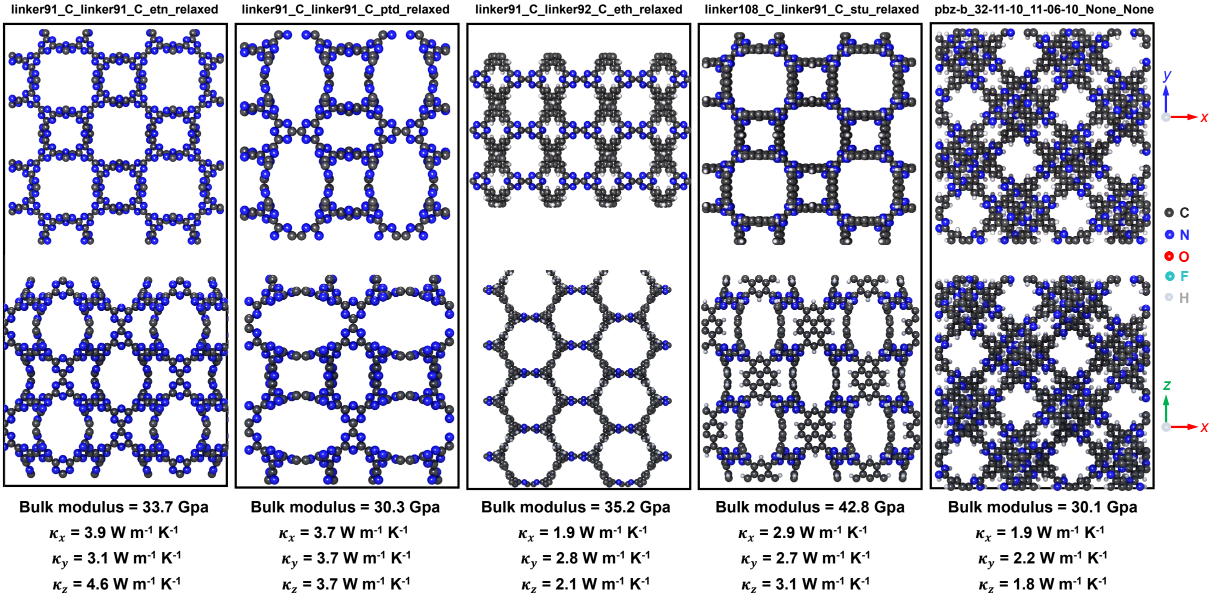

Fig. S6: Schematic illustrations of representative 3D COF structures from region 3 in Fig. 3 of the main text, exhibiting low thermal conductivity anisotropy (anisotropy ratio < 3).

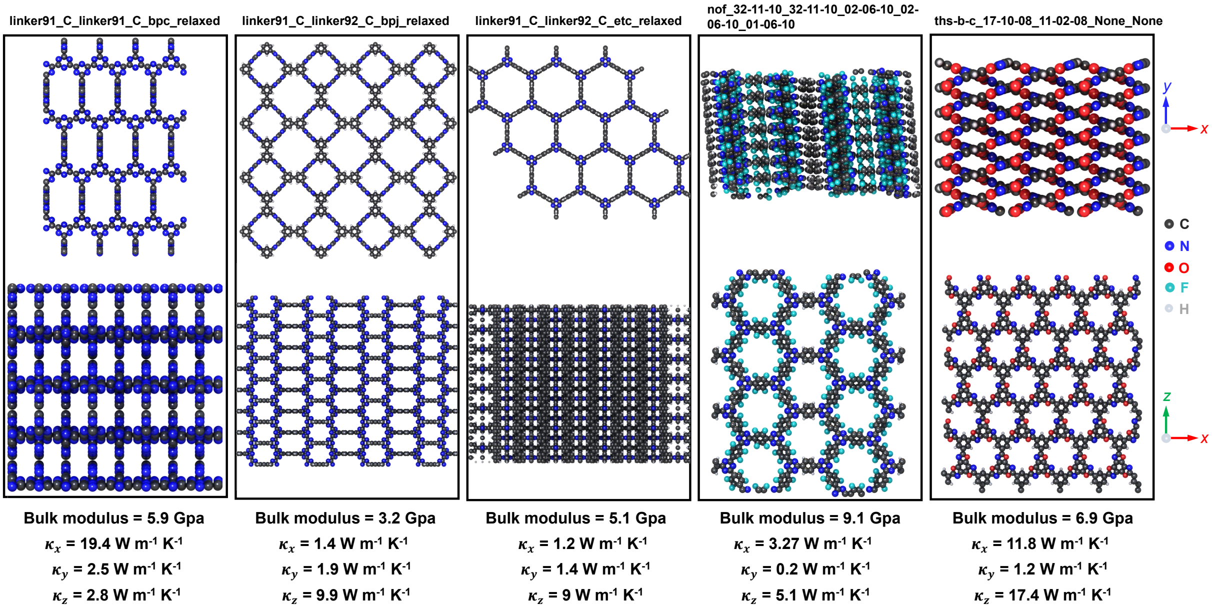

Fig. S7: Schematic illustrations of representative 3D COF structures from region 4 in Fig. 3 of the main text, exhibiting high thermal conductivity anisotropy (anisotropy ratio > 5).

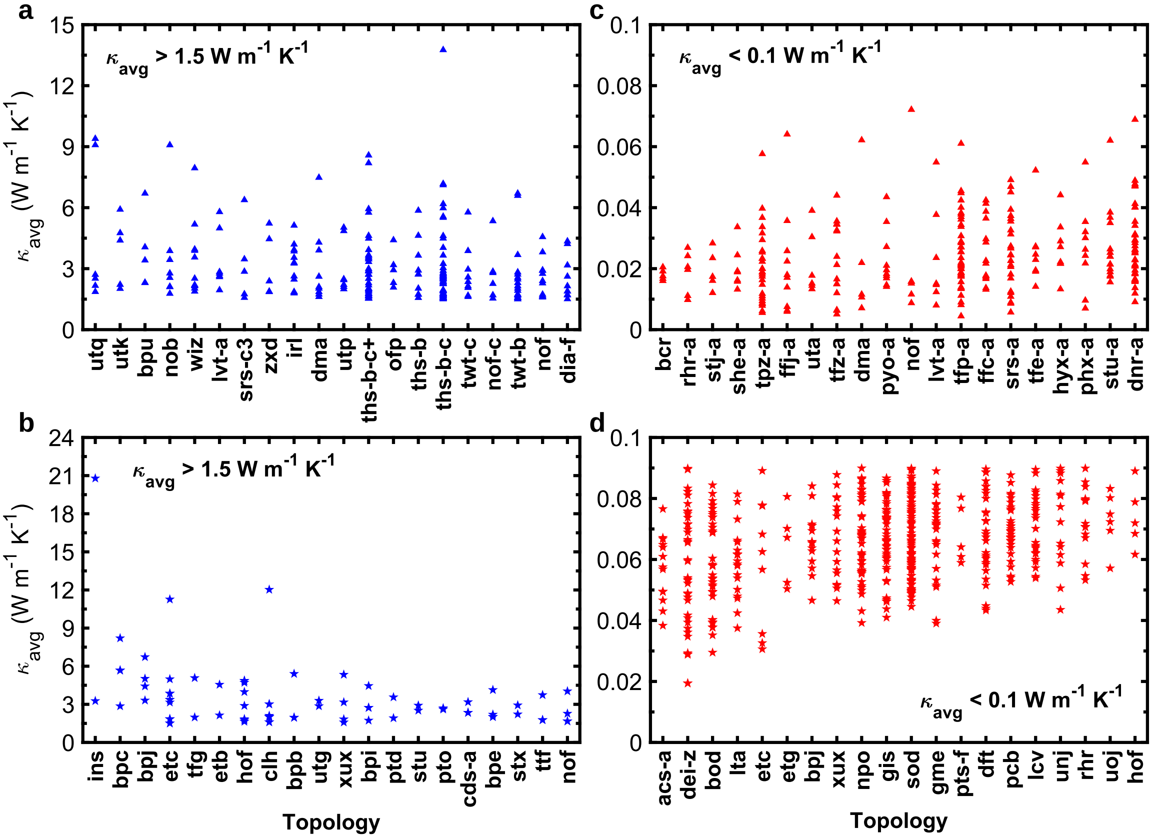

Fig. S8: Distribution of the average thermal conductivity ($\kappa_{\rm avg}$) of 3D COFs for the top 20 topologies with $\kappa_{\rm avg}$>1.5 W m^{-1} K^{-1} from the (a) ReDDCOFFEE and (b) Mercado databases. Distribution of ($\kappa_{\rm avg}$) of 3D COFs for the worst 20 topologies with $\kappa_{\rm avg}$<0.1 W m^{-1} K^{-1} from the (c) ReDDCOFFEE and (d) Mercado databases. Note, topologies with the number of structures less than 2 and satisfying the conditions are eliminated.

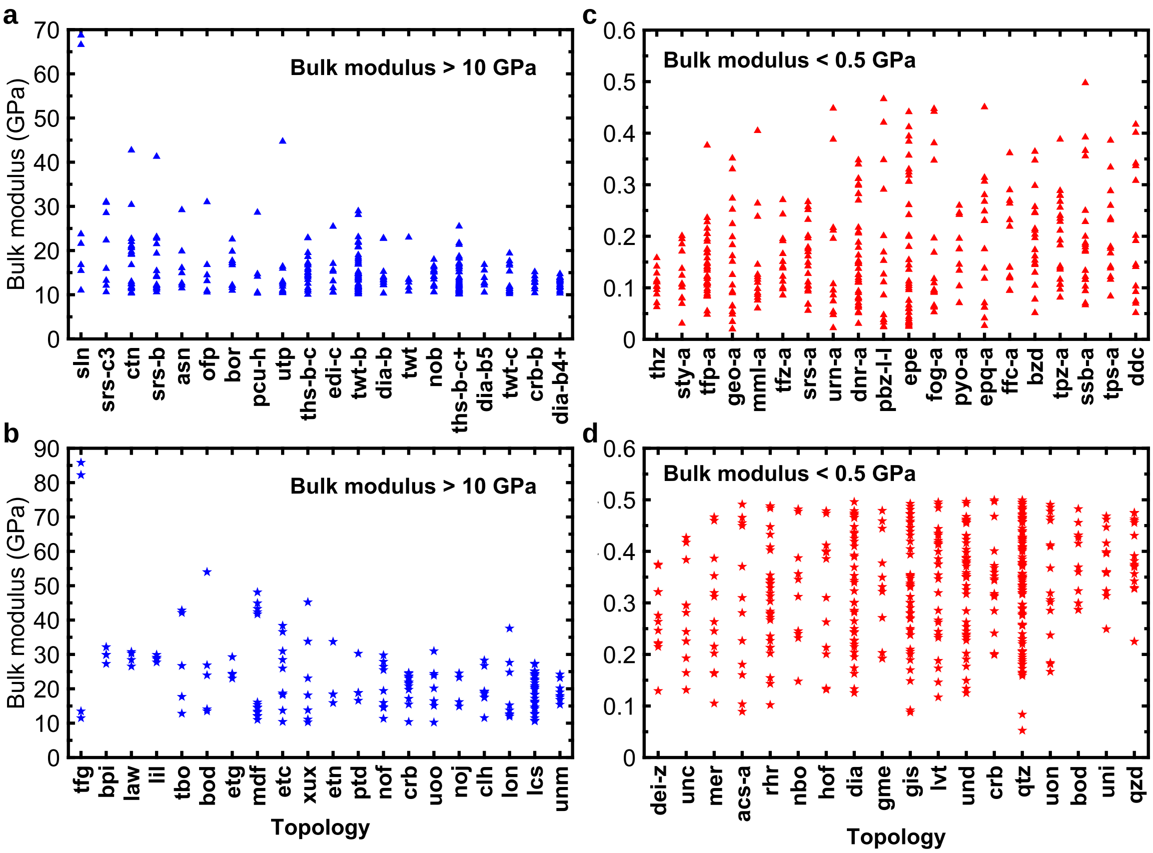

Fig. S9: Distribution of the bulk modulus (BM) of 3D COFs for the top 20 topologies with BM>10 GPa from the (a) ReDDCOFFEE and (b) Mercado databases. Distribution of the bulk modulus of 3D COFs for the worst 20 topologies with BM<0.5 GPa from the (c) ReDDCOFFEE and (d) Mercado databases. Note, topologies with the number of structures less than 2 and satisfying the conditions are eliminated.

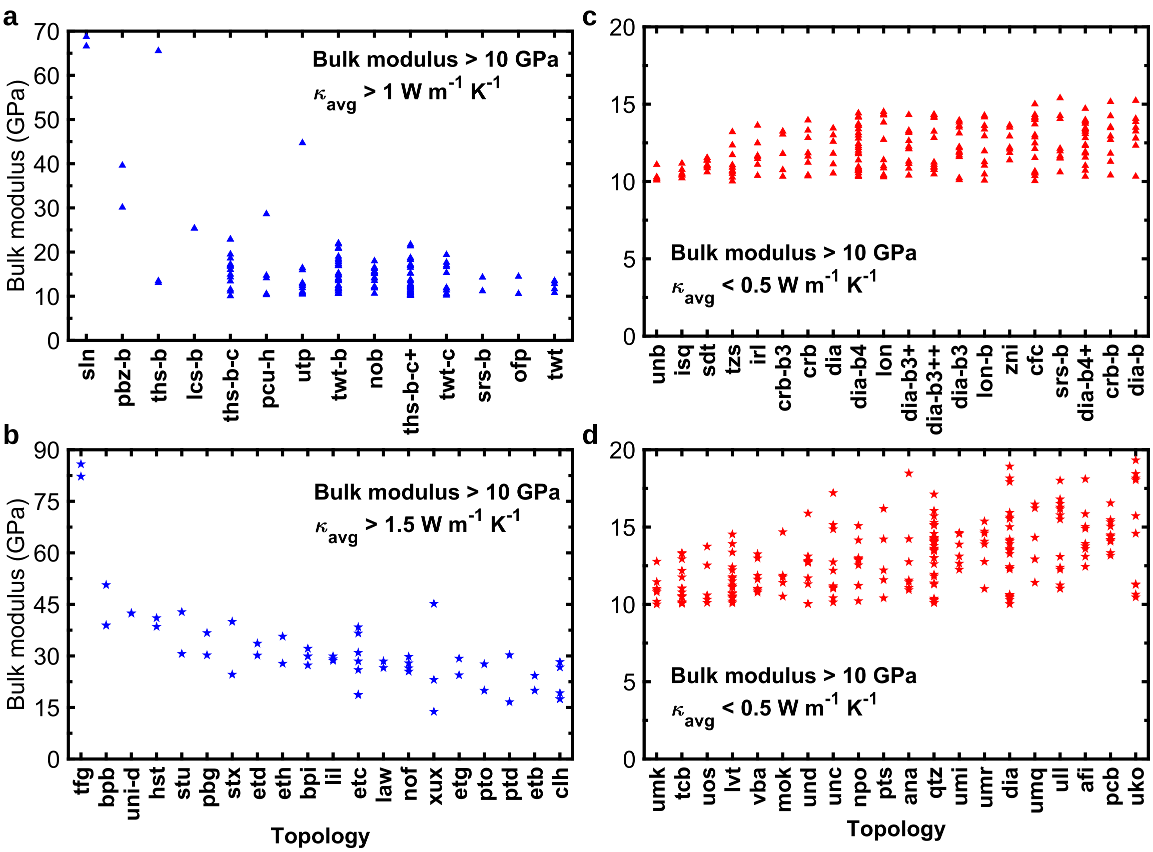

Fig. S10: Distribution of the bulk modulus (BM) of 3D COFs for the top topologies with BM>10 GPa and κ_{avg}>1.5 W m^{-1} K^{-1} from the (a) ReDDCOFFEE and (b) Mercado databases. Distribution of the bulk modulus of 3D COFs for the worst topologies with BM>10 GPa and κ_{avg}<0.5 W m^{-1} K^{-1} from the (c) ReDDCOFFEE and (d) Mercado databases. Note, topologies with the number of structures less than 2 and satisfying the conditions are eliminated.

Schematics of high bulk modulus ($\gtrsim$ 50 GPa) 3D COF structures

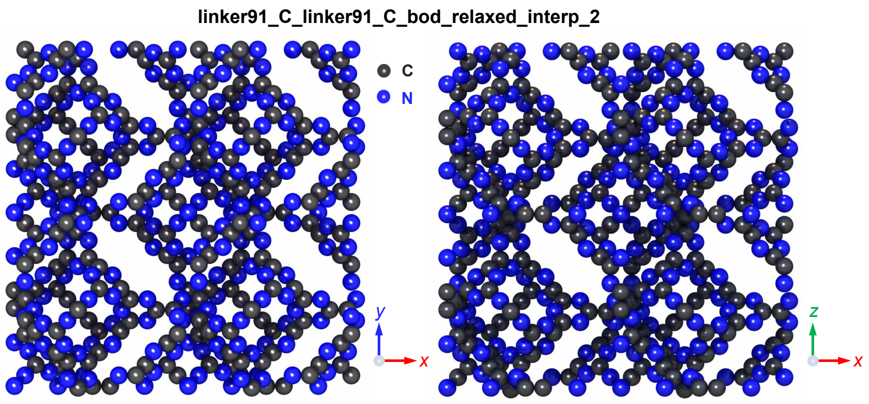

Fig. S11: Schematic illustration of a 3D COF structure exhibiting a high bulk modulus of 54 GPa.

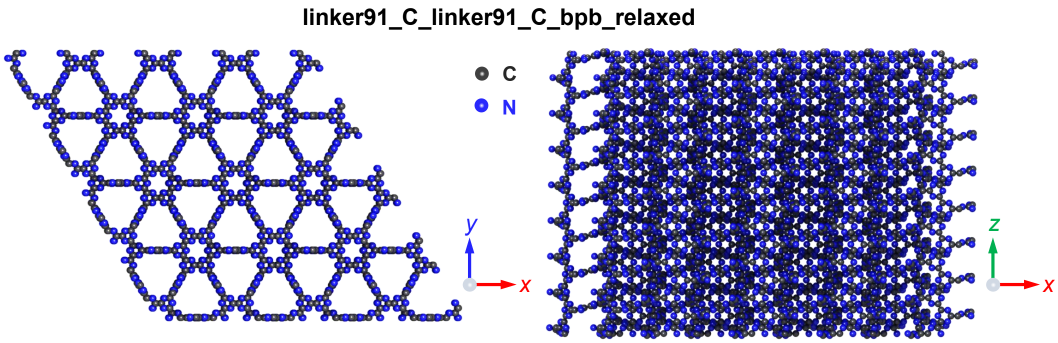

Fig. S12: Schematic illustration of a 3D COF structure exhibiting a high bulk modulus of 50.7 GPa.

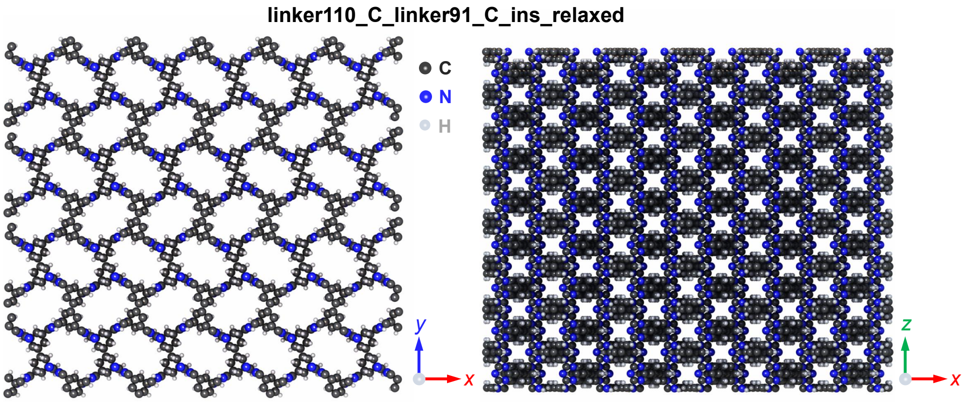

Fig. S13: Schematic illustration of a 3D COF structure exhibiting a high bulk modulus of 51.4 GPa.

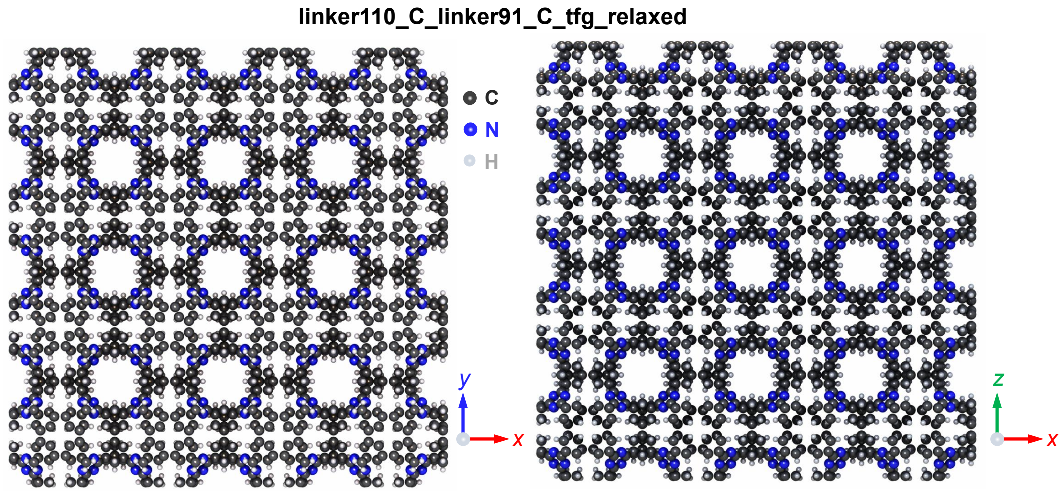

Fig. S14: Schematic illustration of a 3D COF structure exhibiting a high bulk modulus of 82.2 GPa.

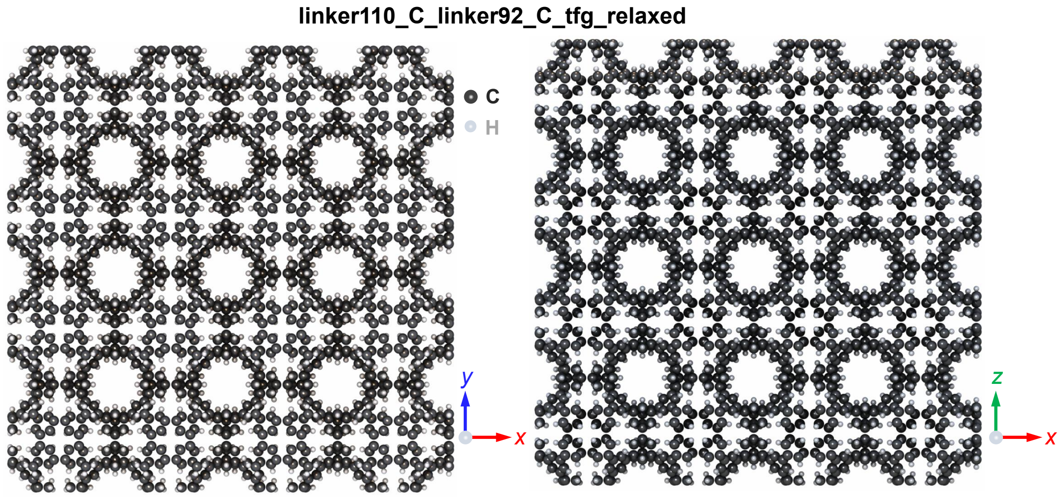

Fig. S15: Schematic illustration of a 3D COF structure exhibiting a high bulk modulus of 85.7 GPa.

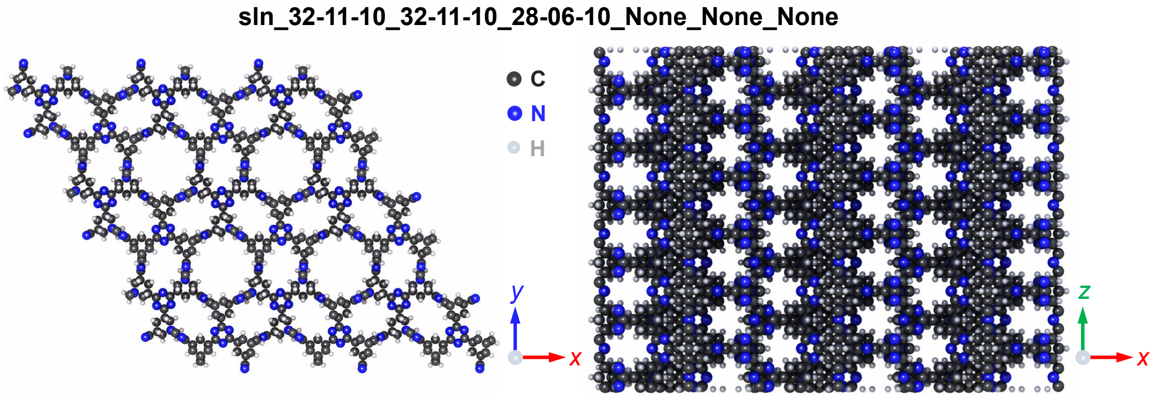

Fig. S16: Schematic illustration of a 3D COF structure exhibiting a high bulk modulus of 68.7 GPa.

sln_33-11-11_33-11-11_28-07-11_None_None_None

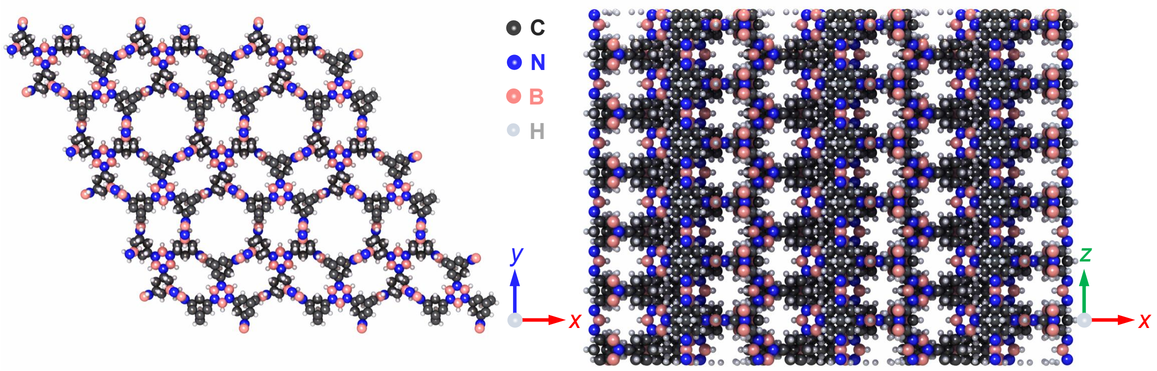

Fig. S17: Schematic illustration of a 3D COF structure exhibiting a high bulk modulus of 66.6 GPa.

ths-b_11-06-10_32-11-10_None_None

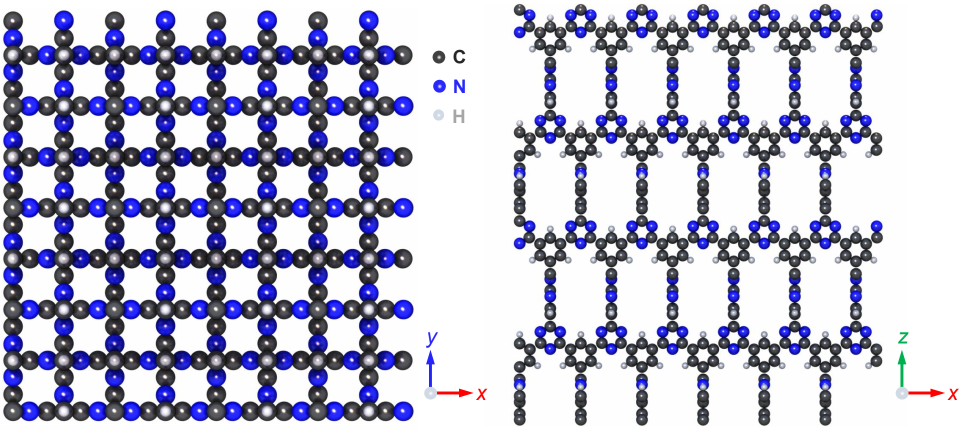

Fig. S18: Schematic illustration of a 3D COF structure exhibiting a high bulk modulus of 65.5 GPa.

Schematics of high thermal conductivity 3D COF structures ($\kappa_{avg} \geq 10$ W m^{-1} K^{-1})

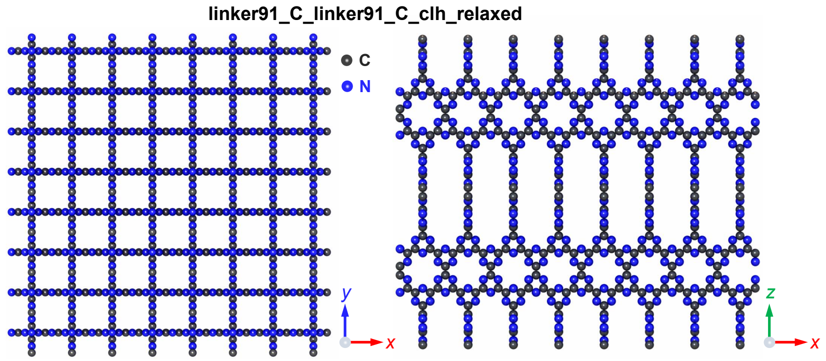

Fig. S19: Schematic illustration of the high thermal conductivity 3D COF structure with x-, y-, and z-directional thermal conductivities of 18.6 W m^{-1} K^{-1}, 12.7 W m^{-1} K^{-1}, and 4.8 W m^{-1} K^{-1}, respectively.

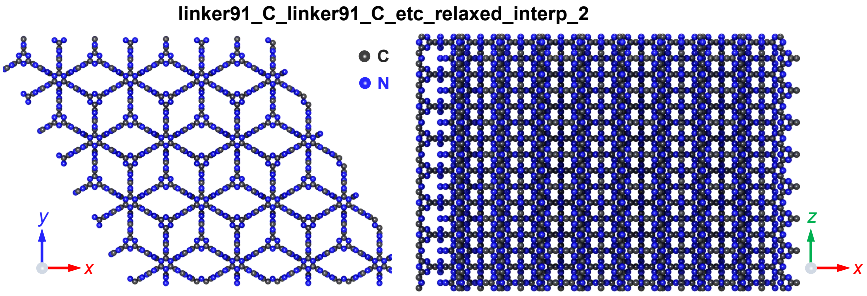

Fig. S20: Schematic illustration of the high thermal conductivity 3D COF structure with x-, y-, and z-directional thermal conductivities of 2.6 W m^{-1} K^{-1}, 3.3 W m^{-1} K^{-1}, and 28.4 W m^{-1} K^{-1}, respectively.

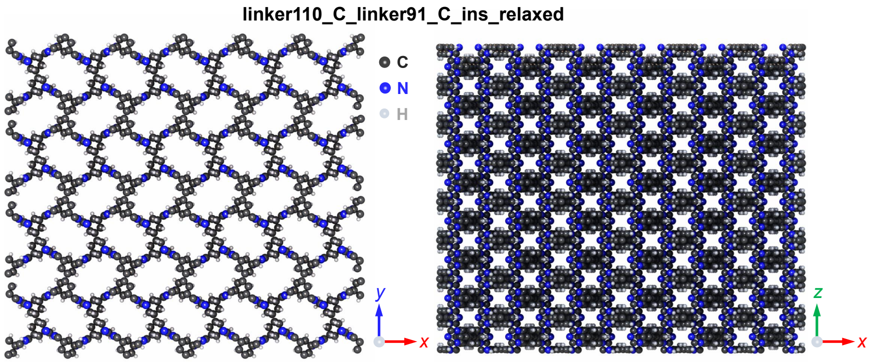

Fig. S21: Schematic illustration of the high thermal conductivity 3D COF structure with x-, y-, and z-directional thermal conductivities of 9.8 W m^{-1} K^{-1}, 5.5 W m^{-1} K^{-1}, and 47 W m^{-1} K^{-1}, respectively.

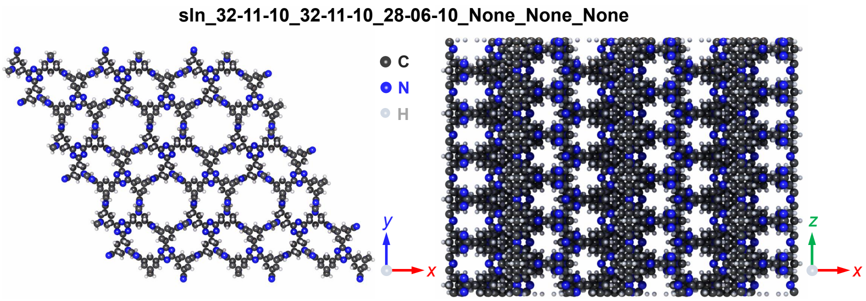

Fig. S22: Schematic illustration of the high thermal conductivity 3D COF structure with κ_{avg} of 12.1 W m^{-1} K^{-1}.

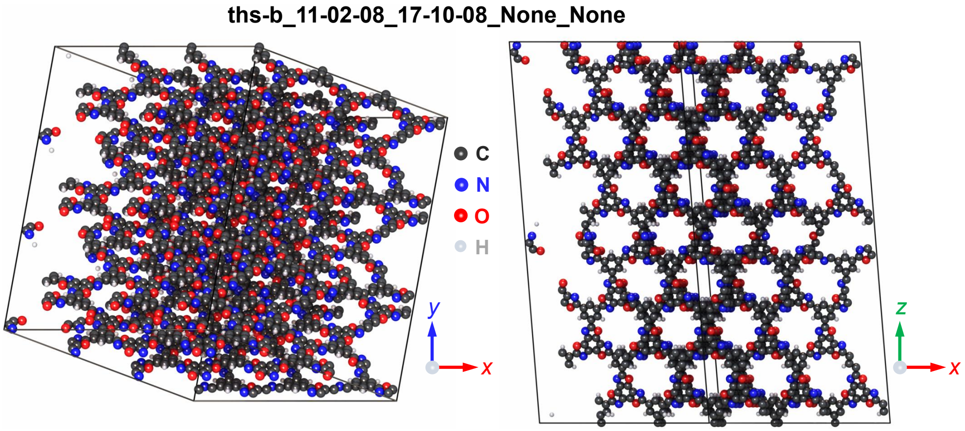

Fig. S23: Schematic illustration of the high thermal conductivity 3D COF structure with x-, y-, and z-directional thermal conductivities of 15.9 W m^{-1} K^{-1}, 5.4 W m^{-1} K^{-1}, and 17.9 W m^{-1} K^{-1}, respectively.

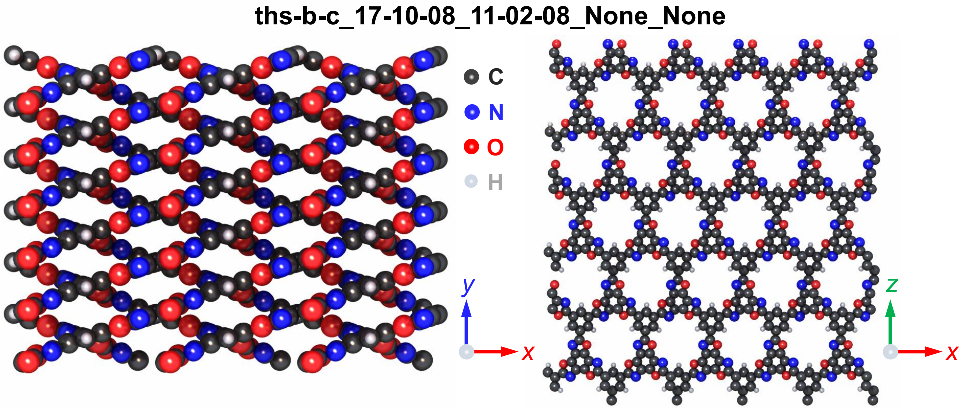

Fig. S24: Schematic illustration of the high thermal conductivity 3D COF structure with x-, y-, and z-directional thermal conductivities of 19 W m^{-1} K^{-1}, 5.1 W m^{-1} K^{-1}, and 17.2 W m^{-1} K^{-1}, respectively.

B. Supplementary Tables

Table S2: Properties of 56 COFs exhibiting high bulk modulus ($\gtrsim$ 30 GPa).

Name	Density (g cm^{-3})	LPD (nm)	Void fraction	GSA (m^2 g^{-1})	Bulk modulus (GPa)	Topology
srs-b_33-11-11_12-07-11_None	0.752	0.594	0.101	2694.024	41.27	srs-b
srs-c3_32-11-10_01-06-10_01-06-10	0.564	0.944	0.230	3860.935	30.85	srs-c3
srs-c3_32-11-10_02-06-10_01-06-10	0.772	1.019	0.189	2300.099	31.04	srs-c3
ctn_17-09-07_28-03-07_None	0.723	0.739	0.221	2884.391	42.70	ctn
ctn_17-10-08_28-02-08_None	0.624	0.735	0.258	3680.269	30.38	ctn
pbz-b_11-06-10_32-11-10_None_None	0.535	1.368	0.348	3551.477	39.60	pbz-b
pbz-b_32-11-10_11-06-10_None_None	0.535	1.368	0.350	3536.993	30.11	pbz-b
ths-b_11-06-10_32-11-10_None_None	0.755	0.591	0.071	3210.657	65.53	ths-b
utp_32-11-10_02-06-10_01-06-10_02-06-10	1.396	0.627	0.062	556.352	44.70	utp
sln_32-11-10_32-11-10_28-06-10_None_None_None	0.988	0.733	0.047	503.838	68.72	sln
sln_33-11-11_33-11-11_28-07-11_None_None_None	0.903	0.772	0.055	546.620	66.59	sln
linker108_C_linker92_C_stu_relaxed	0.624	1.293	0.803	2956.260	30.63	stu
linker91_C_linker92_C_uni-d_relaxed	0.646	0.657	0.815	3745.220	42.39	uni-d
linker110_C_linker87_C_mdf_relaxed	0.965	0.604	0.249	588.795	43.29	mdf
linker110_C_linker81_C_mdf_relaxed	1.005	0.550	0.253	571.765	41.69	mdf
linker110_C_linker81_C_mok_relaxed	0.749	0.716	0.553	2258.410	39.31	mok
linker110_C_linker87_C_mok_relaxed	0.712	0.763	0.557	2250.130	36.66	mok
linker91_C_linker92_C_eth_relaxed	0.563	0.954	0.814	3797.970	35.66	eth
linker92_C_linker91_C_bpb_relaxed	0.695	0.724	0.823	3348.200	38.91	bpb
linker91_C_linker91_C_xaa_relaxed	0.593	1.499	0.870	3619.510	39.07	xaa
linker91_C_linker93_C_etc_relaxed_interp_2	0.591	1.022	0.742	3548.170	30.95	etc
linker92_C_linker92_C_etd_relaxed	0.512	1.075	0.804	3804.800	33.62	etd
linker91_C_linker92_C_law_relaxed	0.690	1.196	0.827	3221.630	30.29	law
linker92_C_linker92_C_pbg_relaxed	0.503	0.994	0.846	3722.190	36.69	pbg
linker91_C_linker91_C_etd_relaxed	0.586	1.090	0.871	3548.200	30.15	etd
linker91_C_linker91_C_pbg_relaxed	0.604	0.999	0.864	3399.630	30.23	pbg
linker91_C_linker91_C_etn_relaxed	0.639	1.289	0.860	3270.740	33.69	etn
linker91_C_linker92_C_bpb_relaxed	0.695	0.724	0.823	3348.200	38.91	bpb
linker110_C_linker91_C_tfg_relaxed	0.914	1.106	0.235	600.345	82.22	tfg
linker110_C_linker2_C_mdf_relaxed	1.066	0.481	0.090	145.668	42.33	mdf
linker92_C_linker92_C_bpi_relaxed	0.705	0.552	0.744	3012.700	32.15	bpi
linker104_C_linker92_C_hst_relaxed	0.728	0.650	0.693	2899.160	41.03	hst
linker91_C_linker91_C_etc_relaxed_interp_2	1.046	0.617	0.619	1414.670	38.38	etc
linker110_C_linker87_C_dia_relaxed_interp_2	0.793	0.629	0.412	1481.340	45.14	dia
linker110_C_linker91_C_ins_relaxed	0.976	0.521	0.453	1102.380	51.37	ins

Continuation of Table S2:

Name	Density ($g\ cm^{-3}$)	LPD (nm)	Void fraction	GSA ($m^2\ g^{-1}$)	Bulk modulus (GPa)	Topology
linker91_C_linker91_C_bod_relaxed_interp_2	1.014	0.559	0.528	1024.660	53.99	bod
linker110_C_linker92_C_tfg_relaxed	0.859	1.128	0.261	710.102	85.81	tfg
linker107_C_linker107_C_lon_relaxed	0.784	0.545	0.574	2029.450	37.57	lon
linker111_C_linker91_C_xux_relaxed_interp_2	0.748	0.903	0.533	1760.120	33.77	xux
linker110_C_linker76_C_mdf_relaxed	1.001	0.601	0.255	625.222	48.10	mdf
linker91_C_linker91_C_bpb_relaxed	0.749	0.722	0.845	3038.510	50.66	bpb
linker91_C_linker91_C_twt_relaxed	1.037	0.398	0.643	922.622	32.13	twt
linker91_C_linker91_C_etc_relaxed	0.523	1.465	0.883	3436.650	36.57	etc
linker108_C_linker91_C_tbo_relaxed	0.644	1.544	0.782	2533.160	42.83	tbo
linker108_C_linker91_C_stu_relaxed	0.679	1.275	0.824	2684.890	42.79	stu
linker91_C_linker91_C_ete_relaxed	0.679	0.727	0.856	3517.040	40.95	ete
linker110_C_linker2_C_uoo_relaxed	0.657	0.715	0.593	2859.770	30.96	uoo
linker108_C_linker91_C_stx_relaxed	0.671	1.217	0.815	2783.170	39.97	stx
linker110_C_linker92_C_xux_relaxed	0.666	0.811	0.708	3189.270	45.20	xux
linker91_C_linker91_C_ptd_relaxed	0.699	1.187	0.657	2307.800	30.25	ptd
linker108_C_linker91_C_ucp_relaxed	0.582	1.741	0.846	2916.450	39.56	ucp
linker92_C_linker91_C_law_relaxed	0.690	1.196	0.826	3221.630	30.77	law
linker101_C_linker92_C_hst_relaxed	0.658	0.666	0.718	3521.410	38.51	hst
linker91_C_linker91_C_nta_relaxed	0.656	1.004	0.862	3561.160	43.89	nta
linker92_C_linker91_C_uni-d_relaxed	0.646	0.657	0.813	3745.220	42.42	uni-d
linker108_C_linker92_C_tbo_relaxed	0.615	1.514	0.744	2812.990	42.11	tbo

Table S3: Properties of 136 COFs exhibiting high thermal conductivities ($\gtrsim$ 5 W m^{-1} K^{-1}).

**Name**	**Density** (g cm^{-3})	**LPD** (nm)	**Void fraction**	**GSA** (m^2 g^{-1})	κ_{avg} (W m^{-1} K^{-1})	κ_{max} (W m^{-1} K^{-1})	**Topology**
ths-b-c+_17-08-01_17-08-01_01-01-01_01-01-01	0.777	1.392	0.284	1706.391	4.76	6.60	ths-b-c+
ths-b-c+_20-08-01_12-01-01_None_None	0.848	1.335	0.248	1359.116	3.70	9.04	ths-b-c+
ths-b-c+_17-08-01_14-01-01_None_None	0.865	1.174	0.221	1410.003	3.47	6.72	ths-b-c+
ths-b-c+_14-01-01_17-08-01_None_None	0.865	1.174	0.224	1395.544	5.94	14.81	ths-b-c+
ths-b-c+_12-01-01_18-08-01_None_None	1.169	0.879	0.112	916.829	3.49	7.27	ths-b-c+
ths-b-c+_18-08-01_12-01-01_None_None	1.169	0.856	0.111	903.565	3.04	5.06	ths-b-c+
twt-b_11-01-01_17-08-01_None_None	1.278	0.570	0.033	597.175	5.23	8.06	twt-b
ths-b_17-08-01_12-01-01_None_None	1.534	0.449	0.004	191.267	3.60	5.23	ths-b
twt-c_31-11-02_01-01-02_01-01-02	0.933	0.858	0.123	1103.720	3.40	5.36	twt-c
bor_31-11-02_28-01-02_None	0.952	0.692	0.041	982.313	5.18	5.18	bor
pcu-h_31-11-02_01-01-02_01-01-02	0.997	0.692	0.072	1048.755	3.44	5.70	pcu-h
clh_31-11-02_31-11-02_01-01-02_01-01-02_01-01-02	1.008	0.670	0.060	1025.882	3.34	5.54	clh
utp_31-11-02_01-01-02_01-01-02_02-01-02	1.191	0.599	0.060	809.502	2.52	5.53	utp
ths-b-c+_31-11-02_31-11-02_02-01-02_01-01-02	1.211	0.597	0.027	639.725	4.56	9.21	ths-b-c+
ths-z_31-11-02_02-01-02_01-01-02	1.401	0.543	0.019	494.345	3.62	5.94	ths-z
clh_31-11-02_31-11-02_02-01-02_02-01-02_01-01-02	1.468	0.538	0.028	493.698	5.77	5.77	clh
ths_31-11-02_02-01-02_02-01-02	1.507	0.617	0.034	526.865	5.98	5.98	ths
ths-b_31-11-02_31-11-02_02-01-02_02-01-02	1.508	0.617	0.051	508.825	4.46	6.82	ths-b
utp_31-11-02_02-01-02_02-01-02_02-01-02	1.509	0.592	0.046	502.807	3.96	6.76	utp
ths-b-c+_31-11-02_31-11-02_02-01-02_02-01-02	1.523	0.598	0.016	416.720	9.39	22.00	ths-b-c+
ths-b_18-09-07_11-03-07_None_None	1.164	0.812	0.112	828.551	2.98	5.08	ths-b
ths-b-c+_17-10-08_14-02-08_None_None	0.887	1.133	0.215	1377.816	4.41	9.13	ths-b-c+
ths-b-c+_11-02-08_11-02-08_08-10-08_08-10-08	0.887	1.298	0.212	1335.784	2.96	5.00	ths-b-c+
ths-b-c_12-02-08_17-10-08_None_None	1.574	0.448	0.001	145.800	3.34	7.88	ths-b-c
ths-b-c+_17-10-08_18-10-08_01-02-08_01-02-08	0.674	1.899	0.408	1662.539	2.70	5.13	ths-b-c+
ths-b_17-10-08_18-10-08_01-02-08_01-02-08	0.686	1.691	0.385	1718.205	4.24	5.07	ths-b
ths-b_18-10-08_17-10-08_01-02-08_01-02-08	0.688	1.684	0.378	1719.113	4.04	6.02	ths-b
ths-b-c_17-10-08_17-10-08_04-02-08_04-02-08	0.700	1.711	0.362	1731.784	3.93	5.02	ths-b-c
nof_17-10-08_17-10-08_01-02-08_01-02-08_01-02-08	0.800	1.265	0.283	1644.286	4.61	6.82	nof
dma_17-10-08_17-10-08_01-02-08_01-02-08_01-02-08_01-02-08_01-02-08	0.800	1.280	0.276	1645.331	4.03	6.24	dma
ths_17-10-08_01-02-08_01-02-08	0.801	1.276	0.276	1629.180	4.68	5.95	ths
ths-b_17-10-08_17-10-08_01-02-08_01-02-08	0.802	1.280	0.287	1638.039	4.63	5.75	ths-b

Continuation of Table S3:

Name	Density (g cm^{-3})	LPD (nm)	Void fraction	GSA (m^2 g^{-1})	κ_{avg} (W m^{-1} K^{-1})	κ_{max} (W m^{-1} K^{-1})	Topology
nof_11-02-08_11-02-08_06-10-08_06-10-08_06-10-08	0.810	1.246	0.273	1660.407	3.18	5.20	nof
clh_11-02-08_11-02-08_06-10-08_06-10-08_06-10-08	0.816	1.189	0.260	1660.336	3.29	5.26	clh
ths-b-c+_11-02-08_11-02-08_07-10-08_06-10-08	0.850	1.267	0.226	1475.922	3.00	5.37	ths-b-c+
ths-b-c_17-10-08_14-02-08_None_None	0.875	1.100	0.217	1387.903	4.49	6.80	ths-b-c
ths-b-c_14-02-08_17-10-08_None_None	0.875	1.099	0.213	1413.857	3.55	5.69	ths-b-c
ths-b_17-10-08_17-10-08_02-02-08_01-02-08	0.895	1.232	0.254	1417.552	3.90	5.90	ths-b
ths-b-c+_17-10-08_17-10-08_02-02-08_01-02-08	0.914	1.200	0.236	1450.800	5.12	6.35	ths-b-c+
ths-b-c_22-10-08_11-02-08_None_None	0.960	0.976	0.123	1089.809	3.92	5.39	ths-b-c
ths-b-c_11-02-08_22-10-08_None_None	0.966	0.913	0.131	1003.287	3.30	5.10	ths-b-c
ths-b_17-10-08_17-10-08_01-02-08_02-02-08	1.016	0.993	0.197	1331.580	5.86	5.86	ths-b
ths-b-c_18-10-08_11-02-08_None_None	1.072	0.828	0.132	1023.738	4.48	7.63	ths-b-c
twt-b_18-10-08_11-02-08_None_None	1.072	0.834	0.138	1021.525	3.57	5.03	twt-b
ths-b-c_11-02-08_18-10-08_None_None	1.088	0.819	0.134	1023.278	3.65	5.82	ths-b-c
ths-b_18-10-08_11-02-08_None_None	1.089	0.790	0.129	1026.252	6.59	10.50	ths-b
ths-b_11-02-08_18-10-08_None_None	1.089	0.791	0.126	994.373	5.51	7.97	ths-b
ths-b-c+_18-10-08_11-02-08_None_None	1.090	0.875	0.121	993.269	4.76	8.32	ths-b-c+
ths-b-c+_17-10-08_17-10-08_02-02-08_02-02-08	1.102	1.126	0.168	1124.583	7.48	12.77	ths-b-c+
ths-b-c_17-10-08_17-10-08_02-02-08_02-02-08	1.106	0.948	0.190	1125.768	4.20	5.19	ths-b-c
ths-b_12-02-08_21-10-08_None_None	1.145	0.850	0.103	805.216	3.65	6.05	ths-b
ths-b-c_12-02-08_18-10-08_None_None	1.167	0.833	0.135	911.321	3.86	6.78	ths-b-c
ths-b_18-10-08_12-02-08_None_None	1.196	0.832	0.120	840.539	4.62	7.14	ths-b
twt-b_17-10-08_11-02-08_None_None	1.359	0.550	0.023	504.710	7.17	10.83	twt-b
twt-b_11-02-08_17-10-08_None_None	1.359	0.550	0.023	500.126	7.11	10.44	twt-b
ths-b-c_17-10-08_11-02-08_None_None	1.377	0.522	0.020	493.292	13.76	18.98	ths-b-c
ths-b-c_11-02-08_17-10-08_None_None	1.377	0.522	0.021	482.763	9.08	17.43	ths-b-c
ths-b_11-02-08_17-10-08_None_None	1.384	0.532	0.024	451.229	13.06	17.87	ths-b
ths-b_17-10-08_11-02-08_None_None	1.384	0.532	0.015	424.189	16.17	17.89	ths-b
ths-b_12-02-08_17-10-08_None_None	1.567	0.495	0.004	173.348	8.19	13.72	ths-b
ths-b-c_17-10-08_12-02-08_None_None	1.574	0.448	0.002	135.349	6.70	12.34	ths-b-c
ths-c_32-11-10_01-06-10_01-06-10	1.008	0.835	0.108	987.879	3.63	7.17	ths-c
nob_32-11-10_32-11-10_02-06-10_02-06-10_02-06-10_01-06-10	1.315	0.803	0.092	691.623	3.67	6.26	nob
ths-b_11-06-10_32-11-10_None_None	0.755	0.591	0.069	3211.935	7.14	14.07	ths-b
ths-b_32-11-10_11-06-10_None_None	0.755	0.591	0.071	3210.657	6.38	11.93	ths-b

Continuation of Table S3:

Name	Density ($g\ cm^{-3}$)	LPD (nm)	Void fraction	GSA ($m^2\ g^{-1}$)	κ_{avg} ($W\ m^{-1}\ K^{-1}$)	κ_{max} ($W\ m^{-1}\ K^{-1}$)	Topology
nob_32-11-10_32-11-10_04-06-10_04-06-10_03-06-10_04-06-10	0.843	1.195	0.219	1383.477	2.66	5.24	nob
nob_32-11-10_32-11-10_04-06-10_04-06-10_04-06-10_04-06-10	0.890	1.172	0.198	1227.641	3.41	5.83	nob
twt-b_14-06-10_32-11-10_None_None	0.961	0.888	0.137	1107.700	3.36	5.88	twt-b
twt-b_32-11-10_14-06-10_None_None	0.961	0.887	0.138	1090.493	5.34	8.16	twt-b
sln_32-11-10_32-11-10_28-06-10_None_None_None	0.988	0.733	0.047	503.838	12.17	12.17	sln
ths-b-c_32-11-10_32-11-10_01-06-10_01-06-10	1.008	0.836	0.123	995.934	5.56	8.55	ths-b-c
utp_32-11-10_01-06-10_01-06-10_01-06-10	1.013	0.795	0.107	1009.092	5.02	9.75	utp
pcu-h_32-11-10_01-06-10_01-06-10	1.016	0.797	0.099	995.474	4.86	8.35	pcu-h
nof_32-11-10_32-11-10_01-06-10_01-06-10_01-06-10	1.018	0.791	0.103	1004.194	6.89	9.89	nof
nob_32-11-10_32-11-10_01-06-10_01-06-10_01-06-10_01-06-10	1.019	0.786	0.102	996.770	6.70	12.59	nob
ths-b_32-11-10_32-11-10_01-06-10_01-06-10	1.020	0.788	0.106	976.754	5.76	7.77	ths-b
twt-c_32-11-10_01-06-10_01-06-10	1.020	0.825	0.110	957.771	6.18	8.96	twt-c
ths-b-c+_32-11-10_32-11-10_01-06-10_01-06-10	1.031	0.813	0.095	963.603	6.39	10.68	ths-b-c+
nob_32-11-10_32-11-10_02-06-10_01-06-10_01-06-10_01-06-10	1.089	0.761	0.087	888.253	3.89	5.62	nob
nof_32-11-10_32-11-10_02-06-10_01-06-10_01-06-10	1.124	0.782	0.078	843.399	4.06	5.75	nof
nob_32-11-10_32-11-10_01-06-10_01-06-10_01-06-10_02-06-10	1.159	0.682	0.079	806.170	3.95	5.59	nob
ths-c_32-11-10_02-06-10_01-06-10	1.174	0.742	0.091	785.511	4.08	6.20	ths-c
bpi_32-11-10_32-11-10_02-06-10_01-06-10_01-06-10_02-06-10	1.225	0.642	0.062	727.677	4.35	7.49	bpi
utp_32-11-10_01-06-10_01-06-10_02-06-10	1.228	0.626	0.074	713.341	4.39	7.43	utp
nob_32-11-10_32-11-10_02-06-10_01-06-10_01-06-10_02-06-10	1.230	0.624	0.062	719.781	3.74	5.76	nob
nob_32-11-10_32-11-10_01-06-10_02-06-10_02-06-10_01-06-10	1.276	0.800	0.094	715.810	4.99	12.32	nob
nof_32-11-10_32-11-10_02-06-10_02-06-10_01-06-10	1.304	0.814	0.097	725.288	2.84	5.10	nof
twt_32-11-10_02-06-10_02-06-10	1.322	0.840	0.108	730.663	4.22	6.27	twt

Continuation of Table S3:

Name	Density (g cm^{-3})	LPD (nm)	Void fraction	GSA (m^2 g^{-1})	κ_{avg} (W m^{-1} K^{-1})	κ_{max} (W m^{-1} K^{-1})	Topology
twt-b_32-11-10_32-11-10_02-06-10_02-06-10	1.325	0.838	0.112	721.779	5.79	8.99	twt-b
dma_32-11-10_32-11-10_02-06-10_02-06-10_02-06-10_02-06-10_02-06-10	1.325	0.861	0.111	719.670	7.95	13.62	dma
nob_32-11-10_32-11-10_01-06-10_02-06-10_02-06-10_02-06-10	1.326	0.837	0.099	709.727	3.47	6.61	nob
nob_32-11-10_32-11-10_02-06-10_02-06-10_02-06-10_02-06-10	1.339	0.845	0.102	709.176	5.56	8.93	nob
nob_32-11-10_32-11-10_02-06-10_01-06-10_02-06-10_02-06-10	1.342	0.630	0.057	608.630	3.88	5.18	nob
ths-c_32-11-10_01-06-10_02-06-10	1.395	0.678	0.062	564.067	4.29	7.91	ths-c
ths-b-c_32-11-10_32-11-10_02-06-10_01-06-10	1.396	0.677	0.062	553.391	5.79	11.19	ths-b-c
twt-c_32-11-10_02-06-10_02-06-10	1.420	0.777	0.083	597.455	5.91	8.85	twt-c
ths-b-c+_32-11-10_32-11-10_01-06-10_02-06-10	1.433	0.545	0.020	451.003	8.58	14.52	ths-b-c+
ths-c_32-11-10_02-06-10_02-06-10	1.455	0.730	0.070	544.888	9.08	14.59	ths-c
linker92_C_linker92_C_etc_relaxed	0.440	1.497	0.844	3774.040	3.15	7.34	etc
linker91_C_linker92_C_etc_relaxed	0.454	1.520	0.865	3641.560	3.85	8.95	etc
linker91_C_linker91_C_noj_relaxed	0.598	1.213	0.856	2973.870	4.08	8.53	noj
linker91_C_linker93_C_etc_relaxed_interp_2	0.591	1.022	0.742	3548.170	3.37	6.14	etc
linker92_C_linker92_C_bpj_relaxed	0.543	1.142	0.812	3650.720	3.31	5.17	bpj
linker99_C_linker91_C_hof_relaxed	0.612	1.033	0.837	3537.270	4.85	10.15	hof
linker91_C_linker91_C_ttf_relaxed	0.556	1.476	0.874	3579.630	3.73	5.96	ttf
linker110_C_linker91_C_tfg_relaxed	0.914	1.106	0.235	600.345	5.08	5.61	tfg
linker110_C_linker92_C_ins_relaxed	0.914	0.517	0.495	1245.240	3.27	5.34	ins
linker110_C_linker91_C_mjb_relaxed	0.734	1.232	0.593	1796.450	7.56	18.22	mjb
linker91_C_linker91_C_utc_relaxed	0.721	0.890	0.793	2809.790	3.82	5.46	utc
linker91_C_linker91_C_etc_relaxed_interp_2	1.046	0.617	0.619	1414.670	11.42	28.43	etc
linker91_C_linker91_C_bpc_relaxed	0.768	0.674	0.839	3315.940	8.21	19.39	bpc
linker110_C_linker91_C_ins_relaxed	0.976	0.521	0.453	1102.380	20.79	47.01	ins
linker91_C_linker92_C_bpc_relaxed	0.775	0.607	0.698	2307.330	5.67	10.77	bpc
linker91_C_linker91_C_nbo-a_relaxed_interp_2	0.784	1.177	0.717	1939.050	4.46	5.60	nbo-a
linker91_C_linker91_C_bpm_relaxed	0.778	0.682	0.826	3213.070	4.58	5.98	bpm
linker91_C_linker91_C_clh_relaxed	0.810	0.599	0.837	3496.370	12.03	18.56	clh
linker91_C_linker91_C_bpb_relaxed	0.749	0.722	0.845	3038.510	5.40	10.80	bpb
linker91_C_linker91_C_bpe_relaxed	0.919	0.569	0.679	1325.770	4.14	5.13	bpe
linker105_C_linker91_C_hof_relaxed	0.654	1.002	0.805	3213.750	4.86	10.33	hof
linker110_C_linker108_C_xai_relaxed	0.524	1.641	0.780	2666.150	4.12	7.10	xai
linker91_C_linker91_C_cds-a_relaxed	0.520	0.973	0.891	4530.110	3.18	5.15	cds-a

Continuation of Table S3:

Name	Density ($g\ cm^{-3}$)	LPD (nm)	Void fraction	GSA ($m^2\ g^{-1}$)	κ_{avg} ($W\ m^{-1}\ K^{-1}$)	κ_{max} ($W\ m^{-1}\ K^{-1}$)	Topology
linker91_C_linker91_C_etb_relaxed	0.616	1.053	0.868	3541.610	4.55	6.76	etb
linker93_C_linker91_C_etc_relaxed_interp_2	0.591	1.022	0.741	3548.170	3.37	6.14	etc
linker91_C_linker91_C_bpj_relaxed	0.632	1.152	0.863	3408.350	6.72	15.35	bpj
linker91_C_linker91_C_etc_relaxed	0.523	1.465	0.883	3436.650	4.98	12.40	etc
linker100_C_linker91_C_hof_relaxed	0.664	1.004	0.800	2655.190	3.98	8.46	hof
linker91_C_linker92_C_bpj_relaxed	0.599	1.184	0.842	3558.260	4.42	9.93	bpj
linker111_C_linker91_C_hof_relaxed	0.623	1.176	0.753	2555.360	2.89	7.93	hof
linker92_C_linker91_C_bpj_relaxed	0.599	1.184	0.843	3558.260	5.02	11.40	bpj
linker91_C_linker91_C_nnd_relaxed	0.616	1.118	0.856	3202.570	4.55	6.26	nnd
linker91_C_linker91_C_bpi_relaxed	0.822	0.567	0.807	2713.650	4.45	6.00	bpi
linker91_C_linker91_C_nof_relaxed_interp_2	1.129	0.565	0.390	719.726	4.03	5.10	nof
linker110_C_linker91_C_xux_relaxed	0.736	0.776	0.741	3034.020	5.34	7.00	xux
linker104_C_linker91_C_hof_relaxed	0.711	0.929	0.748	2208.670	4.71	10.47	hof
linker92_C_linker91_C_bpc_relaxed	0.775	0.607	0.698	2307.330	5.67	10.77	bpc
linker92_C_linker91_C_etc_relaxed	0.454	1.520	0.865	3641.560	3.85	8.95	etc

Table S4: Properties of 46 COFs exhibiting both high bulk modulus (> 30 GPa) and relatively high average thermal conductivity ($\kappa_{avg} > 1\ W\ m^{-1}\ K^{-1}$).

Name	Density ($g\ cm^{-3}$)	LPD (nm)	Void fraction	GSA ($m^2\ g^{-1}$)	Bulk modulus (GPa)	κ_{avg} ($W\ m^{-1}\ K^{-1}$)	Topology
pbz-b_11-06-10_32-11-10_None_None	0.535	1.368	0.348	3551.477	39.60	1.59	pbz-b
pbz-b_32-11-10_11-06-10_None_None	0.535	1.368	0.350	3536.993	30.11	1.86	pbz-b
ths-b_11-06-10_32-11-10_None_None	0.755	0.591	0.071	3210.657	65.53	6.38	ths-b
utp_32-11-10_02-06-10_01-06-10_02-06-10	1.396	0.627	0.062	556.352	44.70	2.65	utp
sln_32-11-10_32-11-10_28-06-10_None_None_None	0.988	0.733	0.047	503.838	68.72	12.17	sln
sln_33-11-11_33-11-11_28-07-11_None_None_None	0.903	0.772	0.055	546.620	66.59	2.70	sln
linker108_C_linker92_C_stu_relaxed	0.624	1.293	0.803	2956.260	30.63	2.51	stu
linker91_C_linker92_C_uni-d_relaxed	0.646	0.657	0.815	3745.220	42.39	2.24	uni-d
linker110_C_linker87_C_mdf_relaxed	0.965	0.604	0.249	588.795	43.29	1.42	mdf
linker110_C_linker81_C_mok_relaxed	0.749	0.716	0.553	2258.410	39.31	1.36	mok
linker110_C_linker87_C_mok_relaxed	0.712	0.763	0.557	2250.130	36.66	1.35	mok
linker91_C_linker92_C_eth_relaxed	0.563	0.954	0.814	3797.970	35.66	2.24	eth
linker92_C_linker91_C_bpb_relaxed	0.695	0.724	0.823	3348.200	38.91	1.96	bpb

Continuation of Table S4:

Name	Density (g cm^{-3})	LPD (nm)	Void fraction	GSA (m^2 g^{-1})	Bulk modulus (GPa)	κ_{avg} (W m^{-1} K^{-1})	Topology
linker91_C_linker91_C_xaa_relaxed	0.593	1.499	0.870	3619.510	39.07	1.48	xaa
linker91_C_linker93_C_etc_relaxed_interp_2	0.591	1.022	0.742	3548.170	30.95	3.37	etc
linker92_C_linker92_C_etd_relaxed	0.512	1.075	0.804	3804.800	33.62	2.22	etd
linker91_C_linker92_C_law_relaxed	0.690	1.196	0.827	3221.630	30.29	1.19	law
linker92_C_linker92_C_pbg_relaxed	0.503	0.994	0.846	3722.190	36.69	1.91	pbg
linker91_C_linker91_C_etd_relaxed	0.586	1.090	0.871	3548.200	30.15	1.99	etd
linker91_C_linker91_C_pbg_relaxed	0.604	0.999	0.864	3399.630	30.23	2.68	pbg
linker91_C_linker91_C_etn_relaxed	0.639	1.289	0.860	3270.740	33.69	3.84	etn
linker91_C_linker92_C_bpb_relaxed	0.695	0.724	0.823	3348.200	38.91	1.96	bpb
linker110_C_linker91_C_tfg_relaxed	0.914	1.106	0.235	600.345	82.22	5.08	tfg
linker92_C_linker92_C_bpi_relaxed	0.705	0.552	0.744	3012.700	32.15	1.73	bpi
linker104_C_linker92_C_hst_relaxed	0.728	0.650	0.693	2899.160	41.03	1.62	hst
linker91_C_linker91_C_etc_relaxed_interp_2	1.046	0.617	0.619	1414.670	38.38	11.26	etc
linker110_C_linker87_C_dia_relaxed_interp_2	0.793	0.629	0.412	1481.340	45.14	2.28	dia
linker110_C_linker91_C_ins_relaxed	0.976	0.521	0.453	1102.380	51.37	20.78	ins
linker91_C_linker91_C_bod_relaxed_interp_2	1.014	0.559	0.528	1024.660	53.99	1.69	bod
linker110_C_linker92_C_tfg_relaxed	0.859	1.128	0.261	710.102	85.81	1.98	tfg
linker107_C_linker107_C_lon_relaxed	0.784	0.545	0.574	2029.450	37.57	1.46	lon
linker91_C_linker91_C_bpb_relaxed	0.749	0.722	0.845	3038.510	50.66	5.40	bpb
linker91_C_linker91_C_twt_relaxed	1.037	0.398	0.643	922.622	32.13	2.53	twt
linker91_C_linker91_C_etc_relaxed	0.523	1.465	0.883	3436.650	36.57	4.98	etc
linker108_C_linker91_C_tbo_relaxed	0.644	1.544	0.782	2533.160	42.83	1.49	tbo
linker108_C_linker91_C_stu_relaxed	0.679	1.275	0.824	2684.890	42.79	2.91	stu
linker91_C_linker91_C_ete_relaxed	0.679	0.727	0.856	3517.040	40.95	2.37	ete
linker108_C_linker91_C_stx_relaxed	0.671	1.217	0.815	2783.170	39.97	2.93	stx
linker110_C_linker92_C_xux_relaxed	0.666	0.811	0.708	3189.270	45.20	3.16	xux
linker91_C_linker91_C_ptd_relaxed	0.699	1.187	0.657	2307.800	30.25	3.56	ptd
linker108_C_linker91_C_ucp_relaxed	0.582	1.741	0.846	2916.450	39.56	1.62	ucp
linker92_C_linker91_C_law_relaxed	0.690	1.196	0.826	3221.630	30.77	1.17	law
linker101_C_linker92_C_hst_relaxed	0.658	0.666	0.718	3521.410	38.51	1.58	hst
linker91_C_linker91_C_nta_relaxed	0.656	1.004	0.862	3561.160	43.89	3.10	nta
linker92_C_linker91_C_uni-d_relaxed	0.646	0.657	0.813	3745.220	42.42	2.24	uni-d
linker108_C_linker92_C_tbo_relaxed	0.615	1.514	0.744	2812.990	42.11	1.80	tbo

III. 2D COFs

Table S5: Distribution of unique building blocks of 2D COFs selected for our high-throughput calculations.

Numbers of unique	ReDDCOFFEE database	Mercado database
Topology	79	25
Linkers	198	500
Bond type	11	5

A. Supplementary Figures

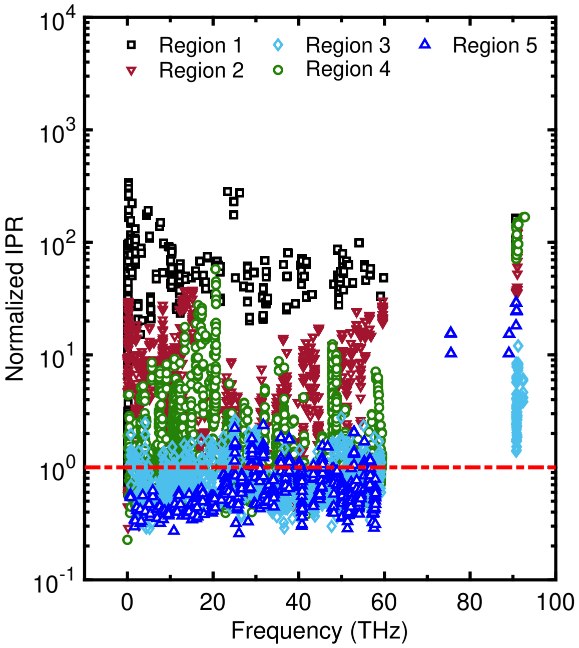

Fig. S25: Comparison of inverse participation ratio (IPR) of 2D COFs corresponding to the regions highlighted in Fig. 9 of the main text. Notably, COFs from region 5, which exhibit the highest $\kappa_{\mathrm{in-plane,avg}}$ among the selected regions, show an absence of phonon localization up to ~22 THz. In contrast, regions with lower $\kappa_{\mathrm{in-plane,avg}}$ show higher IPR values, indicating more localized vibrational modes that contribute to reduced thermal transport. The red dashed line indicates the threshold for localized modes, defined as eigenvectors distributed over less than 20% of the total atoms.[S4, S5].

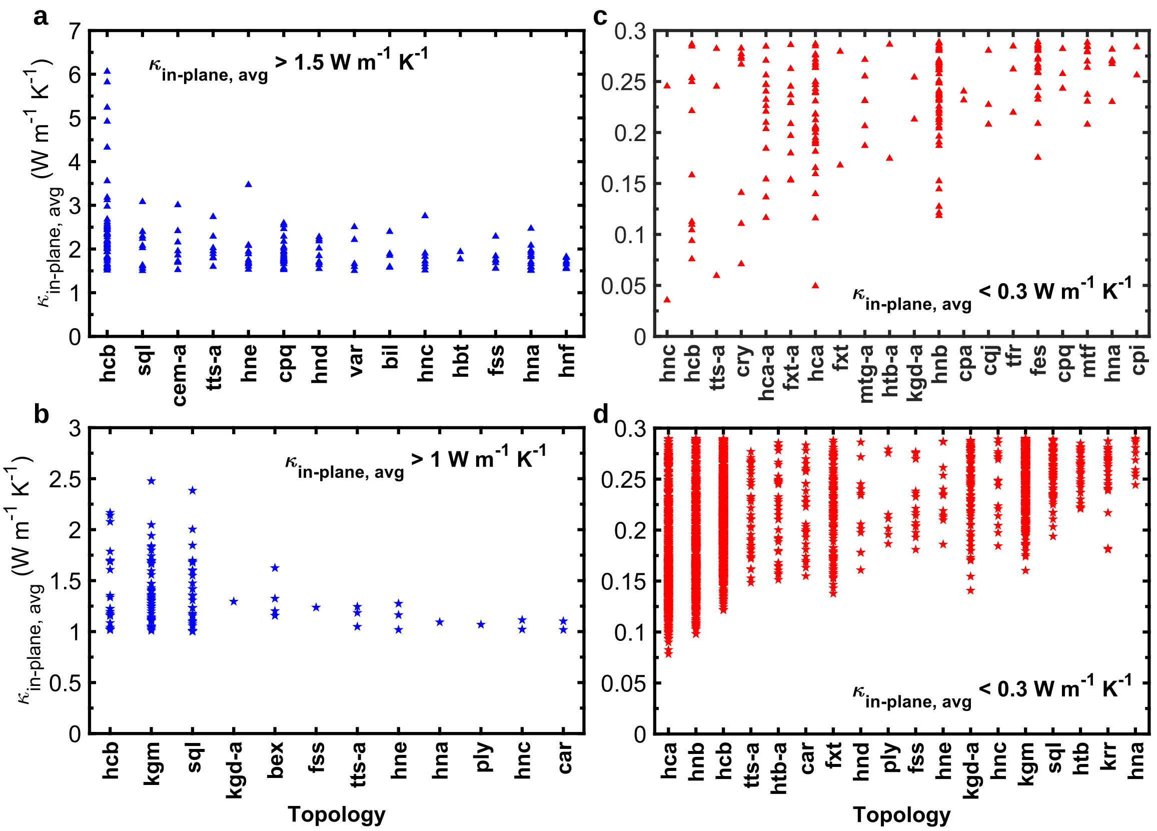

Fig. S26: Distribution of the average in-plane thermal conductivity ($\kappa_{\text{in-plane,avg}}$) of 2D COFs for the top topologies from the (a) ReDDCOFFEE and (b) Mercado databases. Distribution of ($\kappa_{\text{in-plane,avg}}$) of 2D COFs for the worst topologies with $\kappa_{\text{in-plane,avg}}$<0.3 W m^{-1} K^{-1} from the (c) ReDDCOFFEE and (d) Mercado databases.

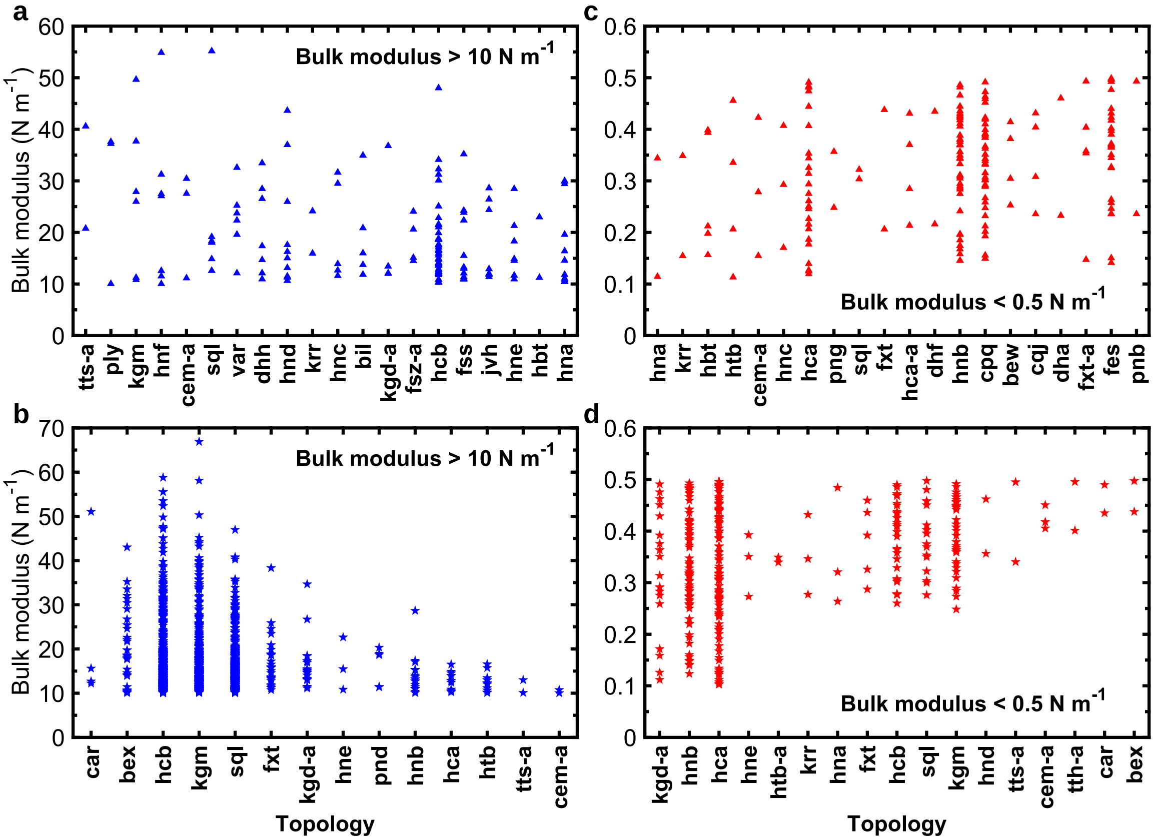

Fig. S27: Distribution of the bulk modulus (BM) of 2D COFs for the top topologies with BM>10 N m^{-1} from the (a) ReDDCOFFEE and (b) Mercado databases. Distribution of the bulk modulus of 2D COFs for the worst topologies with BM<0.5 N m^{-1} from the (c) ReDDCOFFEE and (d) Mercado databases. Note, topologies with the number of structures less than 2 and satisfying the conditions are eliminated.

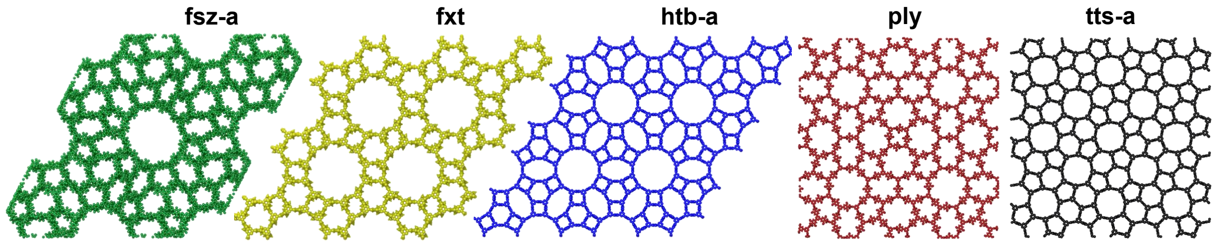

Fig. S28: Schematic illustrations of topologies where pores are predominantly misaligned and exhibit three distinct size ranges. Such structural features can disrupt phonon pathways and reduce thermal conductivity in 2D COFs.

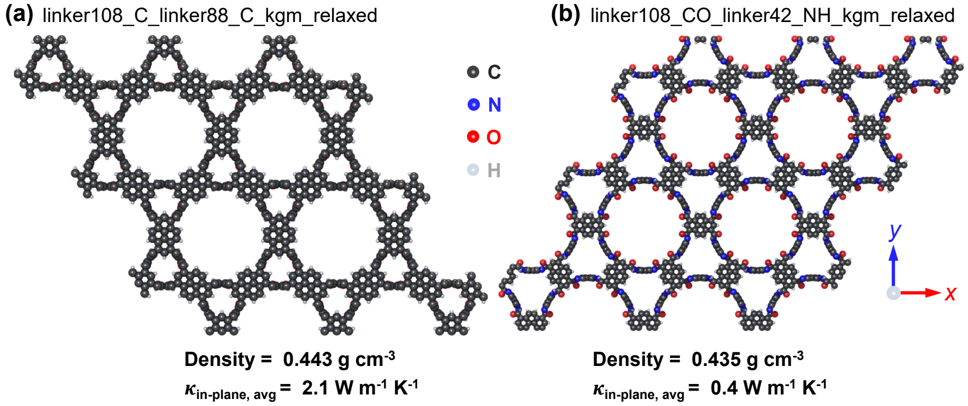

Fig. S29: Schematic illustrations of **kgm** topology for 2D COFs with similar densities but contrasting in-plane thermal conductivities. (a) High-$\kappa_{\mathrm{in-plane,avg}}$ structure (2.1 W m^{-1} K^{-1}) with flat linkers aligned along the heat transport direction, enabling efficient heat conduction. (b) Low-$\kappa_{\mathrm{in-plane,avg}}$ structure (0.4 W m^{-1} K^{-1}) with linkers misaligned relative to the xy-plane, disrupting phonon transport and reducing thermal conductivity.

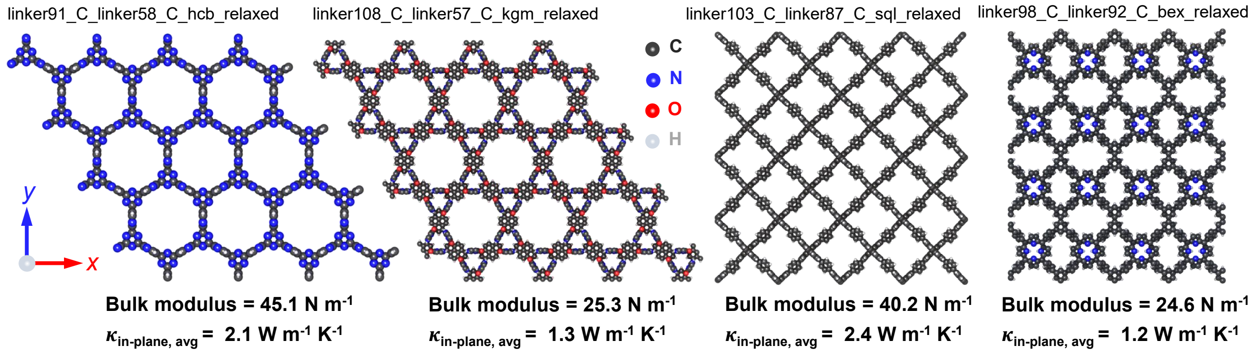

Fig. S30: Schematic illustrations of representative 2D COF structures exhibiting both high bulk modulus ($>$ 20 N m^{-1}) and high average in-plane thermal conductivity ($\kappa_{\mathrm{in-plane,avg}} > 1$ W m^{-1} K^{-1}).

Schematics of high bulk modulus ($\gtrsim 50$ N m^{-1}) 2D COF structures

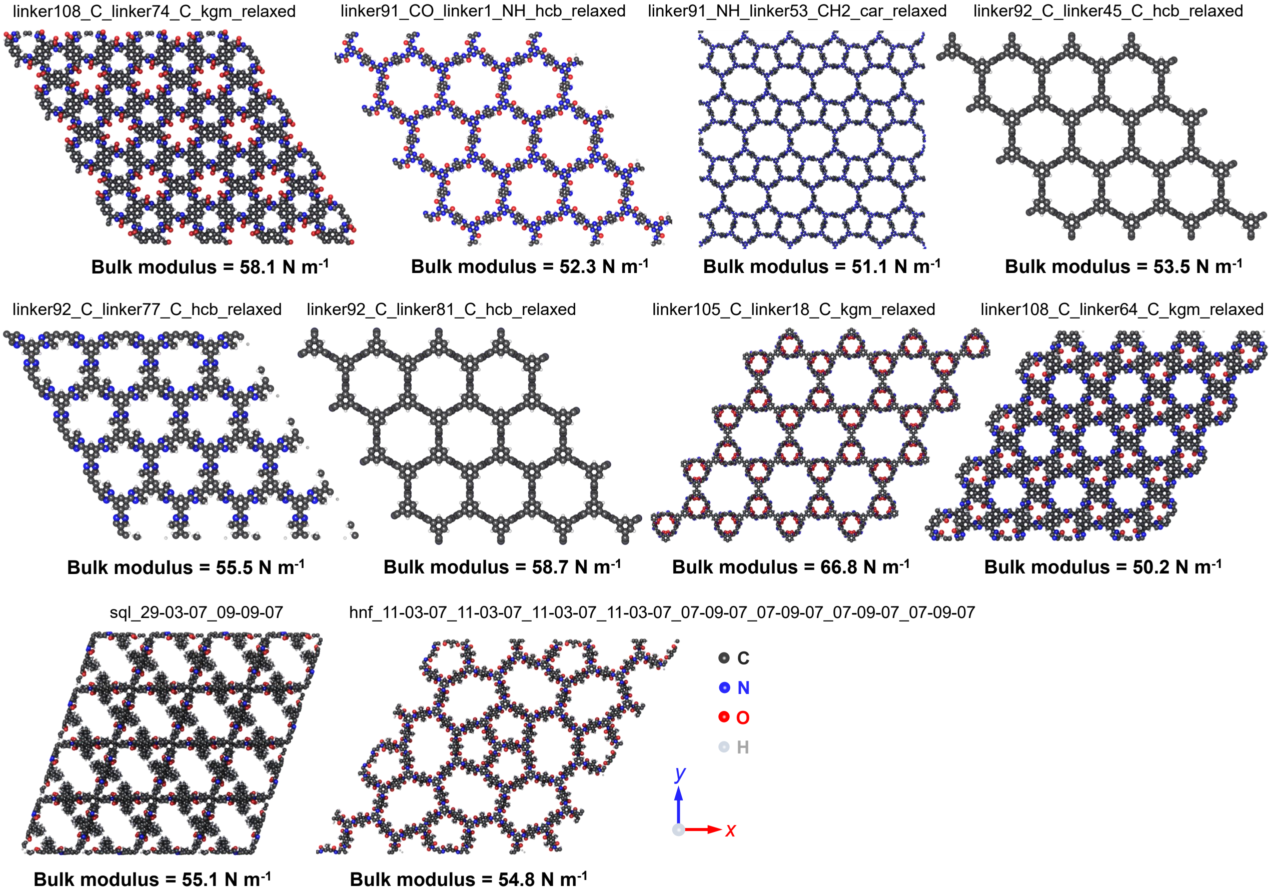

Fig. S31: Schematic illustrations of 2D COF structures with bulk modulus exceeding 50 N m^{-1}.

Schematics of high average in-plane thermal conductivity ($\gtrsim 3$ W m^{-1} K^{-1}) 2D COF structures

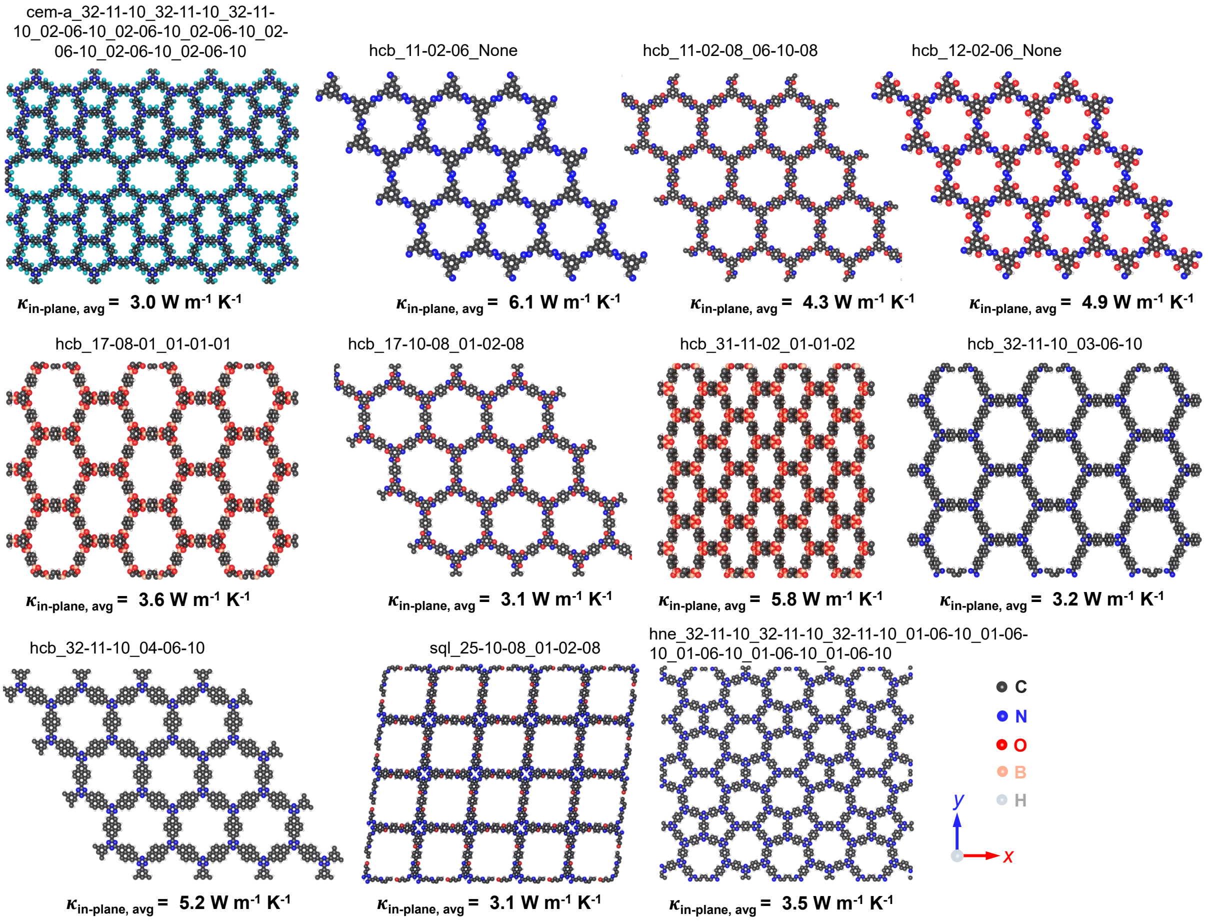

Fig. S32: Schematic illustrations of 2D COF structures with average in-plane thermal conductivity, $\kappa_{\mathrm{in-plane,avg}}$, exceeding 3 W m^{-1} K^{-1}.

B. Supplementary Tables

Table S6: Properties of 131 2D COFs exhibiting high bulk modulus ($\gtrsim$ 30 N m^{-1}).

**Name**	**Density** (g cm^{-3})	**LPD** (nm)	**Void fraction**	**GSA** (m^2 g^{-1})	**Bulk modulus** (N m^{-1})	**Topology**
hcb_17-09-07_04-03-07	0.727	1.667	0.362	1511.745	30.11	hcb
cpi_20-08-01_25-08-01_02-01-01_02-01-01_02-01-01	0.799	1.018	0.192	2057.565	32.94	cpi
dhh_12-03-07_12-03-07_12-03-07_12-03-07_07-09-07_07-09-07_07-09-07_07-09-07_07-09-07_07-09-07	0.802	1.913	0.300	1446.897	33.45	dhh
fss_12-03-07_12-03-07_12-03-07_12-03-07_06-09-07_06-09-07_06-09-07_06-09-07_06-09-07	0.760	1.883	0.349	1501.809	35.19	fss
bil_12-03-07_12-03-07_12-03-07_12-03-07_06-09-07_06-09-07_06-09-07_06-09-07_06-09-07_06-09-07	0.700	3.037	0.388	1526.801	34.94	bil
cem-a_18-09-07_18-09-07_18-09-07_03-03-07_03-03-07_03-03-07_03-03-07_03-03-07_03-03-07	0.780	1.074	0.222	1888.992	30.43	cem-a
cpq_11-03-07_12-03-07_08-09-07_08-09-07_08-09-07_08-09-07	0.739	2.198	0.385	1411.743	32.68	cpq
hcb_11-03-07_07-09-07	0.852	1.302	0.259	1416.713	32.28	hcb
hcb_12-03-07_08-09-07	0.790	1.701	0.352	1411.772	31.25	hcb
hcb_12-03-07_10-09-07	0.547	2.435	0.499	1836.620	48.00	hcb
hnc_12-03-07_12-03-07_12-03-07_12-03-07_06-09-07_06-09-07_06-09-07_06-09-07_06-09-07_06-09-07_06-09-07	0.757	1.871	0.355	1500.558	31.64	hnc
hnf_12-03-07_12-03-07_12-03-07_12-03-07_06-09-07_06-09-07_06-09-07_06-09-07	0.772	1.613	0.335	1514.923	31.26	hnf
kgd-a_12-03-07_12-03-07_06-09-07_06-09-07_06-09-07	0.675	1.877	0.392	1605.330	36.78	kgd-a
kgm_29-03-07_08-09-07	0.639	2.683	0.378	1682.676	49.64	kgm
ply_12-03-07_12-03-07_12-03-07_12-03-07_12-03-07_06-09-07_06-09-07_06-09-07_06-09-07_06-09-07_06-09-07_06-09-07_06-09-07	0.746	2.338	0.338	1521.305	37.20	ply
ply_12-03-07_12-03-07_12-03-07_12-03-07_12-03-07_07-09-07_07-09-07_07-09-07_07-09-07_07-09-07_07-09-07_07-09-07_07-09-07	0.822	2.027	0.266	1432.233	37.58	ply
var_18-09-07_18-09-07_18-09-07_01-03-07_01-03-07_01-03-07_01-03-07_01-03-07	0.803	1.255	0.262	1662.622	32.57	var

Continuation of Table S6:

Name	Density (g cm^{-3})	LPD (nm)	Void fraction	GSA (m^2 g^{-1})	Bulk modulus (N m^{-1})	Topology
hnd_12-03-07_12-03-07_12-03-07_12-03-07_12-03-07_12-03-07_06-09-07_06-09-07_06-09-07_06-09-07_06-09-07_06-09-07_06-09-07_06-09-07_06-09-07_06-09-07	0.763	1.939	0.339	1490.406	43.62	hnd
hnd_12-03-07_12-03-07_12-03-07_12-03-07_12-03-07_12-03-07_07-09-07_07-09-07_07-09-07_07-09-07_07-09-07_07-09-07_07-09-07_07-09-07_07-09-07_07-09-07	0.831	1.656	0.267	1436.025	36.98	hnd
hnf_11-03-07_11-03-07_11-03-07_11-03-07_07-09-07_07-09-07_07-09-07_07-09-07	0.835	1.470	0.252	1406.582	54.86	hnf
kgm_29-02-08_08-10-08	0.594	2.573	0.383	1922.780	37.68	kgm
sql_29-03-07_09-09-07	0.865	0.928	0.138	1004.867	55.17	sql
tts-a_12-03-07_12-03-07_12-03-07_07-09-07_07-09-07_07-09-07_07-09-07_07-09-07	0.816	2.055	0.283	1443.615	40.59	tts-a
hcb_11-03-07_08-09-07	0.814	1.643	0.325	1384.346	34.11	hcb
linker101_N_linker74_CH_kgm_relaxed	0.338	2.165	0.840	5953.640	31.72	kgm
linker103_C_linker23_C_sql_relaxed	0.464	1.017	0.780	5672.730	32.71	sql
linker103_N_linker60_CH_sql_relaxed	0.363	1.393	0.815	5507.930	30.17	sql
linker105_CH_linker96_N_bex_relaxed	0.281	1.516	0.889	7419.280	32.08	bex
linker103_C_linker14_C_sql_relaxed	0.428	1.111	0.764	5339.150	33.89	sql
linker103_C_linker22_C_sql_relaxed	0.411	1.186	0.803	5664.810	31.54	sql
linker103_C_linker40_C_sql_relaxed	0.414	1.405	0.839	4484.300	31.65	sql
linker108_CH_linker2_N_kgm_relaxed	0.303	1.866	0.888	7036.870	31.71	kgm
linker108_CH_linker78_N_kgm_relaxed	0.325	1.866	0.864	6637.930	33.37	kgm
linker108_CO_linker47_NH_kgm_relaxed	0.466	1.945	0.685	3060.070	30.79	kgm
linker108_CO_linker49_NH_kgm_relaxed	0.418	1.940	0.843	4865.330	34.47	kgm
linker108_CO_linker80_NH_sql_relaxed	0.471	1.103	0.748	4618.560	31.46	sql
linker91_CO_linker60_NH_hcb_relaxed	0.300	1.482	0.861	7514.730	31.56	hcb
linker91_CO_linker73_NH_hcb_relaxed	0.347	1.422	0.804	6450.670	32.73	hcb
linker108_CO_linker65_NH_kgm_relaxed	0.404	1.915	0.853	5224.020	34.00	kgm
linker91_C_linker71_C_hcb_relaxed	0.476	0.835	0.803	5379.540	31.53	hcb
linker92_C_linker22_C_kgd-a_relaxed	0.268	1.970	0.869	6551.870	34.66	kgd-a
linker93_C_linker81_C_hcb_relaxed	0.230	2.222	0.894	7914.330	31.72	hcb
linker92_C_linker68_C_hcb_relaxed	0.452	0.991	0.840	5641.720	33.16	hcb
linker93_C_linker85_C_hcb_relaxed	0.265	2.031	0.859	6534.770	32.86	hcb
linker93_C_linker57_C_hcb_relaxed	0.284	1.993	0.860	6352.120	33.85	hcb

Continuation of Table S6:

Name	Density ($g\ cm^{-3}$)	LPD (nm)	Void fraction	GSA ($m^2\ g^{-1}$)	Bulk modulus ($N\ m^{-1}$)	Topology
linker99_CO_linker39_NH_sql_relaxed	0.302	1.577	0.894	7285.290	30.88	sql
linker99_C_linker96_C_bex_relaxed	0.334	1.191	0.878	6744.900	30.44	bex
linker108_CH_linker96_N_bex_relaxed	0.285	1.514	0.895	7241.230	31.40	bex
linker108_C_linker96_C_bex_relaxed	0.357	1.166	0.871	6456.380	33.53	bex
linker108_C_linker16_C_kgm_relaxed	0.360	1.880	0.830	6367.500	30.69	kgm
linker108_C_linker38_C_kgm_relaxed	0.404	1.921	0.832	4899.590	33.82	kgm
linker108_C_linker39_C_kgm_relaxed	0.365	1.975	0.866	5998.540	30.41	kgm
linker91_CO_linker2_NH_hcb_relaxed	0.279	1.651	0.891	7511.310	30.77	hcb
linker91_CO_linker41_NH_hcb_relaxed	0.309	1.589	0.892	7135.610	32.44	hcb
linker91_CO_linker8_NH_hcb_relaxed	0.370	1.393	0.857	6565.180	34.02	hcb
linker91_C_linker25_C_hcb_relaxed	0.351	1.669	0.838	6172.240	34.91	hcb
linker91_C_linker26_C_hcb_relaxed	0.457	1.471	0.803	4709.430	34.54	hcb
linker91_C_linker33_C_hcb_relaxed	0.314	1.906	0.840	5210.140	33.71	hcb
linker91_N_linker12_CH_hcb_relaxed	0.282	1.652	0.883	7571.620	30.65	hcb
linker99_C_linker21_C_kgm_relaxed	0.324	2.036	0.774	5868.880	33.69	kgm
linker91_CO_linker71_NH_hcb_relaxed	0.319	1.360	0.871	7178.530	30.66	hcb
linker91_C_linker35_C_hcb_relaxed	0.281	1.957	0.888	6510.320	33.99	hcb
linker91_C_linker60_C_hcb_relaxed	0.445	1.144	0.759	4923.660	31.57	hcb
linker92_C_linker1_C_hcb_relaxed	0.381	1.287	0.786	6005.870	30.35	hcb
linker92_C_linker35_C_hcb_relaxed	0.272	1.979	0.881	6643.810	34.67	hcb
linker92_C_linker62_C_hcb_relaxed	0.420	1.249	0.792	5314.170	30.70	hcb
linker92_C_linker89_C_hcb_relaxed	0.263	1.747	0.883	7894.130	32.91	hcb
linker91_C_linker10_C_hcb_relaxed	0.597	1.178	0.761	3287.760	30.08	hcb
linker91_C_linker12_C_hcb_relaxed	0.529	1.159	0.718	3442.110	31.25	hcb
linker101_C_linker57_C_kgm_relaxed	0.505	1.702	0.727	3196.000	34.92	kgm
linker104_C_linker80_C_kgm_relaxed	0.554	1.588	0.624	2896.200	32.06	kgm
linker91_C_linker74_C_hcb_relaxed	0.515	0.753	0.756	5897.490	33.29	hcb
linker92_C_linker42_C_hcb_relaxed	0.531	0.817	0.838	5082.870	31.08	hcb
linker92_C_linker6_C_hcb_relaxed	0.556	0.869	0.776	4337.210	32.93	hcb
linker104_C_linker12_C_kgm_relaxed	0.529	1.691	0.724	3332.820	34.48	kgm
linker91_C_linker57_C_hcb_relaxed	0.538	1.212	0.778	3451.370	31.15	hcb
linker91_C_linker46_C_hcb_relaxed	0.514	0.720	0.808	4269.360	30.85	hcb
linker91_C_linker50_C_hcb_relaxed	0.556	0.769	0.738	4550.260	32.42	hcb
linker94_C_linker1_C_hcb_relaxed	0.188	2.618	0.909	8048.370	47.33	hcb
linker103_CH_linker51_N_sql_relaxed	0.439	1.218	0.767	4548.930	35.78	sql
linker103_C_linker37_C_sql_relaxed	0.467	1.075	0.795	5217.640	35.23	sql
linker105_C_linker18_C_kgm_relaxed	0.414	2.272	0.781	3990.950	66.89	kgm
linker108_CO_linker62_NH_kgm_relaxed	0.369	1.926	0.859	5697.320	30.59	kgm

Continuation of Table S6:

Name	Density ($g\ cm^{-3}$)	LPD (nm)	Void fraction	GSA ($m^2\ g^{-1}$)	Bulk modulus ($N\ m^{-1}$)	Topology
linker108_CO_linker69_NH_kgm_relaxed	0.392	1.848	0.829	5587.130	33.95	kgm
linker108_CO_linker82_NH_kgm_relaxed	0.354	1.928	0.855	6210.910	43.88	kgm
linker108_C_linker15_C_kgm_relaxed	0.307	2.325	0.861	6633.050	37.39	kgm
linker91_NH_linker53_CH2_car_relaxed	0.346	2.131	0.775	4956.190	51.06	car
linker92_C_linker11_C_fxt_relaxed	0.407	2.706	0.761	3643.830	38.33	fxt
linker99_CH_linker95_N_bex_relaxed	0.206	1.870	0.909	9001.060	35.24	bex
linker108_CH_linker91_N_bex_relaxed	0.421	1.059	0.876	5331.790	43.02	bex
linker103_C_linker87_C_sql_relaxed	0.484	0.929	0.769	5086.080	40.25	sql
linker108_CH_linker72_N_kgm_relaxed	0.352	1.947	0.870	5845.860	35.82	kgm
linker91_CO_linker1_NH_hcb_relaxed	0.279	1.636	0.887	7659.580	52.34	hcb
linker92_C_linker85_C_hcb_relaxed	0.467	1.155	0.755	4708.100	39.78	hcb
linker103_C_linker76_C_sql_relaxed	0.492	0.968	0.791	4902.880	46.97	sql
linker91_C_linker20_C_hcb_relaxed	0.277	1.618	0.848	7443.710	31.40	hcb
linker92_C_linker66_C_hcb_relaxed	0.410	0.976	0.813	6701.370	37.13	hcb
linker92_C_linker77_C_hcb_relaxed	0.371	1.058	0.843	6933.300	55.53	hcb
linker92_C_linker81_C_hcb_relaxed	0.352	1.312	0.844	7381.620	58.78	hcb
linker91_C_linker58_C_hcb_relaxed	0.368	1.282	0.863	7125.840	45.10	hcb
linker92_C_linker39_C_hcb_relaxed	0.275	1.992	0.890	6961.490	35.25	hcb
linker92_C_linker41_C_hcb_relaxed	0.446	1.236	0.789	4899.540	43.76	hcb
linker92_C_linker45_C_hcb_relaxed	0.475	1.221	0.748	4074.450	53.50	hcb
linker92_C_linker58_C_hcb_relaxed	0.351	1.302	0.843	7419.040	47.14	hcb
linker92_C_linker59_C_hcb_relaxed	0.390	1.243	0.790	5942.750	35.63	hcb
linker92_C_linker82_C_hcb_relaxed	0.394	1.242	0.797	5974.380	47.69	hcb
linker92_C_linker84_C_hcb_relaxed	0.394	1.277	0.810	6094.550	37.25	hcb
linker92_C_linker88_C_hcb_relaxed	0.427	1.277	0.771	5069.260	42.71	hcb
linker108_C_linker81_C_kgm_relaxed	0.443	1.248	0.870	5198.800	38.73	kgm
linker108_C_linker87_C_kgm_relaxed	0.447	1.307	0.802	5648.110	43.22	kgm
linker103_C_linker45_C_sql_relaxed	0.582	0.941	0.716	3035.080	40.70	sql
linker108_C_linker8_C_kgm_relaxed	0.567	1.560	0.693	2888.950	36.79	kgm
linker91_C_linker70_C_hcb_relaxed	0.522	0.787	0.718	5159.210	49.80	hcb
linker92_C_linker10_C_hcb_relaxed	0.573	1.193	0.739	3444.360	38.50	hcb
linker92_C_linker72_C_hcb_relaxed	0.567	0.825	0.779	4880.950	36.17	hcb
linker103_C_linker62_C_sql_relaxed	0.545	0.866	0.749	4422.560	40.77	sql
linker108_C_linker64_C_kgm_relaxed	0.518	1.242	0.808	4712.240	50.29	kgm
linker108_C_linker74_C_kgm_relaxed	0.592	1.008	0.726	3925.330	58.11	kgm
linker108_C_linker71_C_kgm_relaxed	0.555	1.003	0.775	4646.950	45.22	kgm
linker108_C_linker72_C_kgm_relaxed	0.572	1.625	0.729	3119.650	35.60	kgm
linker91_C_linker8_C_hcb_relaxed	0.574	0.850	0.798	4481.430	36.47	hcb

Continuation of Table S6:

Name	Density (g cm^{-3})	LPD (nm)	Void fraction	GSA (m^2 g^{-1})	Bulk modulus (N m^{-1})	Topology
linker108_C_linker13_C_kgm_relaxed	0.585	1.147	0.590	2876.800	40.62	kgm
linker108_C_linker45_C_kgm_relaxed	0.576	1.220	0.731	3555.920	38.33	kgm
linker108_C_linker60_C_kgm_relaxed	0.529	1.158	0.703	4307.640	44.24	kgm
linker108_C_linker83_C_kgm_relaxed	0.576	1.095	0.783	3695.690	39.68	kgm
linker108_C_linker9_C_kgm_relaxed	0.512	1.174	0.732	4329.490	36.67	kgm
linker103_C_linker65_C_sql_relaxed	0.597	0.735	0.725	4065.040	35.64	sql
linker91_C_linker65_C_hcb_relaxed	0.508	1.200	0.768	3758.410	41.83	hcb
linker91_C_linker53_C_hcb_relaxed	0.627	0.756	0.637	3900.280	38.80	hcb
linker108_C_linker52_C_kgm_relaxed	0.642	1.052	0.681	2918.520	39.05	kgm
linker108_C_linker49_C_kgm_relaxed	0.617	1.216	0.719	3102.920	39.97	kgm

Table S7: Properties of 78 2D COFs exhibiting high average in-plane thermal conductivity ($\gtrsim$ 2 W m^{-1} K^{-1}).

Name	Density (g cm^{-3})	LPD (nm)	Void fraction	GSA (m^2 g^{-1})	$\kappa_{\mathrm{in-plane,avg}}$ (W m^{-1} K^{-1})	Topology
hcb_30-02-08_10-10-08	0.464	2.304	0.547	2044.060	2.20	hcb
hnd_21-08-01_21-08-01_21-08-01_21-08-01_21-08-01_21-08-01_03-01-01_03-01-01_03-01-01_03-01-01_03-01-01_03-01-01_03-01-01_03-01-01_03-01-01_03-01-01	0.449	4.375	0.554	1719.318	2.22	hnd
jvh_13-01-01_13-01-01_08-08-01_08-08-01_08-08-01_08-08-01_08-08-01	0.416	3.720	0.586	2084.921	2.02	jvh
hcb_22-08-01_01-01-01	0.475	3.545	0.543	1706.564	2.00	hcb
cem-a_11-02-06_11-02-06_11-02-06_None_None_None_None_None_None	0.794	0.993	0.150	1643.038	2.16	cem-a
cem-a_32-11-10_32-11-10_32-11-10_01-06-10_01-06-10_01-06-10_01-06-10_01-06-10_01-06-10	0.987	1.048	0.116	1022.795	2.41	cem-a
cem-a_32-11-10_32-11-10_32-11-10_02-06-10_02-06-10_02-06-10_02-06-10_02-06-10_02-06-10	1.232	1.059	0.121	850.702	3.01	cem-a
cpq_32-11-10_32-11-10_01-06-10_02-06-10_01-06-10_01-06-10	1.165	0.994	0.122	751.927	2.05	cpq
cpq_32-11-10_32-11-10_01-06-10_02-06-10_01-06-10_02-06-10	1.153	1.104	0.148	773.274	2.46	cpq

Continuation of Table S7:

Name	Density (g cm^{-3})	LPD (nm)	Void fraction	GSA (m^2 g^{-1})	$\kappa_{\mathrm{in-plane,avg}}$ (W m^{-1} K^{-1})	Topology
cpq_32-11-10_32-11-10_01-06-10_02-06-10_02-06-10_02-06-10	1.141	1.185	0.158	833.280	2.29	cpq
cpq_32-11-10_32-11-10_02-06-10_01-06-10_01-06-10_01-06-10	1.042	0.915	0.123	915.768	2.59	cpq
cpq_32-11-10_32-11-10_02-06-10_02-06-10_01-06-10_01-06-10	1.158	0.935	0.130	806.342	2.01	cpq
cpq_32-11-10_32-11-10_02-06-10_02-06-10_02-06-10_02-06-10	1.135	1.087	0.167	869.766	2.55	cpq
cpq_32-11-10_32-11-10_03-06-10_03-06-10_04-06-10_03-06-10	0.684	1.758	0.339	1581.539	2.17	cpq
fss_17-10-08_17-10-08_17-10-08_17-10-08_01-02-08_01-02-08_01-02-08_01-02-08_01-02-08	0.779	1.660	0.295	1627.330	2.29	fss
hcb_11-01-01_07-08-01	0.896	0.874	0.156	1359.950	2.36	hcb
hcb_11-01-01_08-08-01	0.870	1.227	0.222	1413.385	2.34	hcb
hcb_11-02-08_07-10-08	0.942	0.874	0.130	1152.183	2.03	hcb
hcb_12-01-01_07-08-01	0.997	0.866	0.154	1139.251	2.10	hcb
hcb_12-01-01_08-08-01	0.956	1.141	0.210	1216.761	2.37	hcb
hcb_17-09-07_04-03-07	0.727	1.667	0.362	1511.745	2.43	hcb
hcb_18-09-07_01-03-07	0.665	2.081	0.419	1535.585	2.46	hcb
hcb_30-02-08_06-10-08	0.548	1.786	0.476	1983.871	2.54	hcb
hcb_30-02-08_08-10-08	0.565	2.069	0.470	1882.057	2.32	hcb
hcb_30-03-07_08-09-07	0.602	1.984	0.465	1735.249	2.10	hcb
hcb_33-11-11_04-07-11	0.746	1.381	0.274	1581.968	2.00	hcb
hna_12-02-06_11-02-06_None_None_None	0.836	1.003	0.189	1385.893	2.08	hna
hnc_32-11-10_32-11-10_32-11-10_32-11-10_01-06-10_01-06-10_01-06-10_01-06-10_01-06-10_01-06-10_01-06-10	0.981	0.934	0.125	1030.783	2.76	hnc
hnd_17-10-08_17-10-08_17-10-08_17-10-08_17-10-08_17-10-08_04-02-08_04-02-08_04-02-08_04-02-08_04-02-08_04-02-08_04-02-08_04-02-08_04-02-08_04-02-08	0.690	2.191	0.363	1739.083	2.18	hnd
hnd_31-11-02_31-11-02_31-11-02_31-11-02_31-11-02_31-11-02_01-01-02_01-01-02_01-01-02_01-01-02_01-01-02_01-01-02_01-01-02_01-01-02_01-01-02_01-01-02	1.000	0.771	0.064	994.421	2.28	hnd

Continuation of Table S7:

Name	Density (g cm^{-3})	LPD (nm)	Void fraction	GSA (m^2 g^{-1})	$\kappa_{in-plane,avg}$ (W m^{-1} K^{-1})	Topology
hnd_32-11-10_32-11-10_32-11-10_32-11-10_32-11-10_32-11-10_04-06-10_04-06-10_04-06-10_04-06-10_04-06-10_04-06-10_04-06-10_04-06-10_04-06-10_04-06-10	0.848	1.668	0.219	1276.296	2.02	hnd
hne_11-02-06_11-02-06_12-02-06_None_None_None_None_None	0.881	1.123	0.164	1355.943	2.09	hne
hne_32-11-10_32-11-10_32-11-10_03-06-10_03-06-10_03-06-10_03-06-10_03-06-10	0.691	1.779	0.293	1701.431	2.08	hne
sql_25-09-07_04-03-07	0.753	1.710	0.350	1408.006	2.08	sql
sql_25-10-08_01-02-08	0.777	1.498	0.323	1457.585	3.08	sql
sql_25-10-08_03-02-08	0.650	1.929	0.404	1640.881	2.29	sql
sql_25-10-08_04-02-08	0.722	1.667	0.360	1539.370	2.24	sql
sql_29-02-08_06-10-08	0.639	1.274	0.296	2304.389	2.03	sql
sql_29-02-08_10-10-08	0.551	1.589	0.390	2437.817	2.40	sql
tts-a_11-02-06_11-02-06_12-02-06_None_None_None_None_None	0.804	1.306	0.188	1558.448	2.29	tts-a
tts-a_17-10-08_17-10-08_17-10-08_02-02-08_02-02-08_02-02-08_02-02-08_02-02-08	1.072	1.740	0.195	1099.422	2.02	tts-a
tts-a_32-11-10_32-11-10_32-11-10_01-06-10_01-06-10_01-06-10_01-06-10_01-06-10	0.967	1.289	0.137	1029.473	2.74	tts-a
var_31-11-02_31-11-02_31-11-02_04-01-02_04-01-02_04-01-02_04-01-02_04-01-02	0.787	2.426	0.277	1167.201	2.21	var
var_32-11-10_32-11-10_32-11-10_02-06-10_02-06-10_02-06-10_02-06-10_02-06-10	1.054	1.587	0.212	922.250	2.51	var
cpq_11-02-06_11-02-06_None_None_None_None	0.702	1.281	0.242	1766.672	2.55	cpq
dhh_11-02-06_11-02-06_11-02-06_11-02-06_None_None_None_None_None_None	0.709	1.374	0.233	1796.472	2.06	dhh
bil_31-11-02_31-11-02_31-11-02_31-11-02_01-01-02_01-01-02_01-01-02_01-01-02_01-01-02_01-01-02	0.925	1.296	0.152	725.261	2.40	bil
hcb_11-01-01_10-08-01	0.558	2.315	0.462	1943.702	2.49	hcb
hcb_11-02-06_02-02-06	0.542	2.248	0.476	2043.783	2.06	hcb
hcb_11-02-06_None	0.738	1.032	0.205	1779.298	6.06	hcb
hcb_11-02-08_06-10-08	0.774	1.413	0.308	1665.774	4.33	hcb
hcb_11-02-08_08-10-08	0.852	1.429	0.247	1463.240	2.02	hcb
hcb_11-02-08_10-10-08	0.581	2.264	0.452	1861.223	2.58	hcb

Continuation of Table S7:

Name	Density (g cm^{-3})	LPD (nm)	Void fraction	GSA (m^2 g^{-1})	$\kappa_{\mathrm{in-plane,avg}}$ (W m^{-1} K^{-1})	Topology
hcb_11-03-07_06-09-07	0.766	1.587	0.340	1522.193	2.10	hcb
hcb_11-03-07_08-09-07	0.814	1.643	0.325	1384.346	2.13	hcb
hcb_12-02-06_None	0.949	0.982	0.180	1317.425	4.92	hcb
hcb_17-08-01_01-01-01	0.780	1.229	0.286	1713.770	3.56	hcb
hcb_17-08-01_04-01-01	0.644	2.046	0.407	1793.195	2.68	hcb
hcb_17-10-08_01-02-08	0.759	1.512	0.318	1649.063	3.12	hcb
hcb_17-10-08_04-02-08	0.671	1.987	0.396	1729.384	2.97	hcb
hcb_18-08-01_01-01-01	0.552	2.411	0.481	1801.369	2.51	hcb
hcb_18-08-01_02-01-01	0.705	1.943	0.430	1569.679	2.17	hcb
hcb_18-08-01_04-01-01	0.503	2.892	0.530	1821.709	2.21	hcb
hcb_18-10-08_01-02-08	0.574	2.351	0.473	1765.651	2.62	hcb
hcb_18-10-08_04-02-08	0.519	2.832	0.519	1780.876	2.28	hcb
hcb_22-08-01_02-01-01	0.553	2.969	0.508	1434.276	2.17	hcb
hcb_31-11-02_01-01-02	1.048	0.511	0.035	810.151	5.82	hcb
hcb_32-11-10_03-06-10	0.705	1.340	0.303	1685.740	3.18	hcb
hcb_32-11-10_04-06-10	0.863	1.360	0.220	1304.145	5.24	hcb
hna_11-02-06_11-02-06_None_None_None	0.711	1.120	0.234	1770.397	2.47	hna
hne_32-11-10_32-11-10_32-11-10_01-06-10_01-06-10_ 01-06-10_01-06-10_01-06-10	0.966	1.078	0.138	1029.769	3.47	hne
linker103_C_linker81_C_sql_relaxed	0.488	1.009	0.798	4896.870	2.00	sql
linker108_C_linker88_C_kgm_relaxed	0.443	1.710	0.783	4515.920	2.05	kgm
linker103_C_linker87_C_sql_relaxed	0.484	0.929	0.769	5086.080	2.38	sql
linker91_C_linker58_C_hcb_relaxed	0.368	1.282	0.863	7125.840	2.14	hcb
linker91_C_linker87_C_hcb_relaxed	0.358	1.292	0.840	7248.690	2.17	hcb
linker91_C_linker12_C_hcb_relaxed	0.529	1.159	0.718	3442.110	2.08	hcb
linker108_C_linker83_C_kgm_relaxed	0.576	1.095	0.783	3695.690	2.48	kgm

Table S8: Properties of 66 2D COFs exhibiting both high bulk modulus ($\geq$ 20 N m^{-1}) and relatively high average in-plane thermal conductivity ($\gtrsim$ 1 W m^{-1} K^{-1}).

Name	Density (g cm^{-3})	LPD (nm)	Void fraction	GSA (m^2 g^{-1})	Bulk modulus (N m^{-1})	$\kappa_{\mathrm{in-plane,avg}}$ (W m^{-1} K^{-1})	Topology
hcb_21-10-08_03-02-08	0.468	3.654	0.563	1657.805	25.08	1.72	hcb
hcb_17-09-07_04-03-07	0.727	1.667	0.362	1511.745	30.11	2.43	hcb
bex_25-09-07_21-09-07_04-03-07_04-03-07	0.598	3.423	0.475	1406.006	24.25	1.21	bex

Continuation of Table S8:

Name	Density (g cm^{-3})	LPD (nm)	Void fraction	GSA (m^2 g^{-1})	Bulk modulus (N m^{-1})	$\kappa_{\mathrm{in-plane,avg}}$ (W m^{-1} K^{-1})	Topology
bil_12-03-07_12-03-07_12-03-07_12-03-07_06-09-07_06-09-07_06-09-07_06-09-07_06-09-07_06-09-07	0.700	3.037	0.388	1526.801	34.94	1.12	bil
cem-a_12-03-07_12-03-07_12-03-07_07-09-07_07-09-07_07-09-07_07-09-07_07-09-07_07-09-07	0.830	1.664	0.256	1457.909	27.54	1.20	cem-a
cpq_11-03-07_12-03-07_07-09-07_07-09-07_07-09-07_07-09-07	0.784	1.650	0.303	1435.904	29.39	1.06	cpq
fxt_12-03-07_08-09-07_08-09-07_08-09-07	0.620	4.396	0.475	1457.956	20.41	1.13	fxt
hcb_11-03-07_07-09-07	0.852	1.302	0.259	1416.713	32.28	1.83	hcb
hcb_12-03-07_08-09-07	0.790	1.701	0.352	1411.772	31.25	1.83	hcb
hcb_12-03-07_10-09-07	0.547	2.435	0.499	1836.620	48.00	1.06	hcb
hcb_21-09-07_02-03-07	0.653	2.470	0.460	1378.222	21.02	1.02	hcb
hcb_30-03-07_08-09-07	0.602	1.984	0.465	1735.249	21.60	2.10	hcb
hnc_12-03-07_12-03-07_12-03-07_12-03-07_06-09-07_06-09-07_06-09-07_06-09-07_06-09-07_06-09-07_06-09-07	0.757	1.871	0.355	1500.558	31.64	1.13	hnc
hne_17-09-07_17-09-07_17-09-07_01-03-07_01-03-07_01-03-07_01-03-07_01-03-07	1.144	0.667	0.037	731.201	21.29	1.94	hne
hnf_12-03-07_12-03-07_12-03-07_12-03-07_06-09-07_06-09-07_06-09-07_06-09-07	0.772	1.613	0.335	1514.923	31.26	1.06	hnf
hnf_12-03-07_12-03-07_12-03-07_12-03-07_08-09-07_08-09-07_08-09-07_08-09-07	0.781	1.874	0.339	1425.043	27.47	1.09	hnf
hnc_12-03-07_12-03-07_12-03-07_12-03-07_07-09-07_07-09-07_07-09-07_07-09-07_07-09-07_07-09-07_07-09-07	0.828	1.590	0.282	1433.773	29.49	1.23	hnc
var_18-09-07_18-09-07_18-09-07_01-03-07_01-03-07_01-03-07_01-03-07_01-03-07	0.803	1.255	0.262	1662.622	32.57	1.01	var

Continuation of Table S8:

Name	Density ($g\ cm^{-3}$)	LPD (nm)	Void fraction	GSA ($m^2\ g^{-1}$)	Bulk modulus ($N\ m^{-1}$)	$\kappa_{in-plane,avg}$ ($W\ m^{-1}\ K^{-1}$)	Topology
hnd_12-03-07_12-03-07_12-03-07_12-03-07_12-03-07_12-03-07_06-09-07_06-09-07_06-09-07_06-09-07_06-09-07_06-09-07_06-09-07_06-09-07_06-09-07_06-09-07	0.763	1.939	0.339	1490.406	43.62	1.19	hnd
hnd_12-03-07_12-03-07_12-03-07_12-03-07_12-03-07_12-03-07_07-09-07_07-09-07_07-09-07_07-09-07_07-09-07_07-09-07_07-09-07_07-09-07_07-09-07_07-09-07	0.831	1.656	0.267	1436.025	36.98	1.45	hnd
sql_29-03-07_09-09-07	0.865	0.928	0.138	1004.867	55.17	1.40	sql
tts-a_12-03-07_12-03-07_12-03-07_07-09-07_07-09-07_07-09-07_07-09-07_07-09-07	0.816	2.055	0.283	1443.615	40.59	1.36	tts-a
var_17-09-07_17-09-07_17-09-07_01-03-07_01-03-07_01-03-07_01-03-07_01-03-07	1.041	0.892	0.111	1070.214	23.74	1.59	var
fes_11-03-07_08-09-07_08-09-07	0.710	2.428	0.394	1394.141	20.84	1.00	fes
hcb_11-03-07_08-09-07	0.814	1.643	0.325	1384.346	34.11	2.13	hcb
hcb_18-09-07_02-03-07	0.585	2.372	0.455	1768.714	22.82	1.44	hcb
linker103_C_linker15_C_sql_relaxed	0.377	1.197	0.812	6208.380	27.50	1.07	sql
linker103_C_linker16_C_sql_relaxed	0.394	1.120	0.799	6189.280	25.68	1.31	sql
linker103_C_linker19_C_sql_relaxed	0.346	1.320	0.838	6409.870	24.70	1.55	sql
linker103_C_linker20_C_sql_relaxed	0.391	1.103	0.787	6207.770	24.05	1.15	sql
linker103_C_linker35_C_sql_relaxed	0.383	1.399	0.847	5169.310	20.57	1.60	sql
linker103_C_linker38_C_sql_relaxed	0.431	1.217	0.820	5536.980	25.46	1.09	sql
linker103_C_linker39_C_sql_relaxed	0.396	1.301	0.843	5684.960	29.32	1.01	sql
linker103_C_linker81_C_sql_relaxed	0.488	1.009	0.798	4896.870	26.88	2.00	sql
linker103_C_linker40_C_sql_relaxed	0.414	1.405	0.839	4484.300	31.65	1.23	sql
linker103_C_linker89_C_sql_relaxed	0.383	1.288	0.823	5409.150	26.80	1.35	sql
linker98_C_linker92_C_bex_relaxed	0.431	1.064	0.830	5983.670	24.61	1.16	bex
linker108_C_linker96_C_bex_relaxed	0.357	1.166	0.871	6456.380	33.53	1.20	bex
linker108_C_linker38_C_kgm_relaxed	0.404	1.921	0.832	4899.590	33.82	1.01	kgm
linker108_C_linker39_C_kgm_relaxed	0.365	1.975	0.866	5998.540	30.41	1.13	kgm
linker103_C_linker30_C_sql_relaxed	0.363	1.344	0.838	6062.040	24.77	1.68	sql
linker91_C_linker12_C_hcb_relaxed	0.529	1.159	0.718	3442.110	31.25	2.08	hcb
linker108_C_linker57_C_kgm_relaxed	0.518	1.729	0.746	3008.110	25.35	1.31	kgm
linker92_C_linker42_C_hcb_relaxed	0.531	0.817	0.838	5082.870	31.08	1.34	hcb

Continuation of Table S8:

Name	Density (g cm^{-3})	LPD (nm)	Void fraction	GSA (m^2 g^{-1})	Bulk modulus (N m^{-1})	$\kappa_{\mathrm{in-plane,avg}}$ (W m^{-1} K^{-1})	Topology
linker103_C_linker87_C_sql_relaxed	0.484	0.929	0.769	5086.080	40.25	2.38	sql
linker103_C_linker76_C_sql_relaxed	0.492	0.968	0.791	4902.880	46.97	1.68	sql
linker92_C_linker66_C_hcb_relaxed	0.410	0.976	0.813	6701.370	37.13	1.18	hcb
linker91_C_linker58_C_hcb_relaxed	0.368	1.282	0.863	7125.840	45.10	2.14	hcb
linker108_C_linker81_C_kgm_relaxed	0.443	1.248	0.870	5198.800	38.73	1.83	kgm
linker108_C_linker87_C_kgm_relaxed	0.447	1.307	0.802	5648.110	43.22	1.69	kgm
linker103_C_linker45_C_sql_relaxed	0.582	0.941	0.716	3035.080	40.70	1.85	sql
linker108_C_linker8_C_kgm_relaxed	0.567	1.560	0.693	2888.950	36.79	1.35	kgm
linker91_C_linker70_C_hcb_relaxed	0.522	0.787	0.718	5159.210	49.80	1.69	hcb
linker103_C_linker62_C_sql_relaxed	0.545	0.866	0.749	4422.560	40.77	1.06	sql
linker108_C_linker64_C_kgm_relaxed	0.518	1.242	0.808	4712.240	50.29	1.67	kgm
linker108_C_linker74_C_kgm_relaxed	0.592	1.008	0.726	3925.330	58.11	1.40	kgm
linker108_C_linker71_C_kgm_relaxed	0.555	1.003	0.775	4646.950	45.22	1.25	kgm
linker108_C_linker72_C_kgm_relaxed	0.572	1.625	0.729	3119.650	35.60	1.47	kgm
linker108_C_linker13_C_kgm_relaxed	0.585	1.147	0.590	2876.800	40.62	1.70	kgm
linker108_C_linker45_C_kgm_relaxed	0.576	1.220	0.731	3555.920	38.33	1.70	kgm
linker108_C_linker60_C_kgm_relaxed	0.529	1.158	0.703	4307.640	44.24	1.94	kgm
linker108_C_linker83_C_kgm_relaxed	0.576	1.095	0.783	3695.690	39.68	2.48	kgm
linker108_C_linker9_C_kgm_relaxed	0.512	1.174	0.732	4329.490	36.67	1.34	kgm
linker103_C_linker65_C_sql_relaxed	0.597	0.735	0.725	4065.040	35.64	1.60	sql
linker108_C_linker52_C_kgm_relaxed	0.642	1.052	0.681	2918.520	39.05	1.74	kgm
linker108_C_linker49_C_kgm_relaxed	0.617	1.216	0.719	3102.920	39.97	1.69	kgm

References

[S1] Sääskilahti, K., Oksanen, J., Tulkki, J. & Volz, S. Role of anharmonic phonon scattering in the spectrally decomposed thermal conductance at planar interfaces. *Physical Review B* **90** (13), 134312 (2014) .

[S2] Giri, A., Braun, J. L. & Hopkins, P. E. Implications of interfacial bond strength on the spectral contributions to thermal boundary conductance across solid, liquid, and gas interfaces: A molecular dynamics study. *The Journal of Physical Chemistry C* **120** (43), 24847–24856 (2016) .

[S3] Gale, J. D. & Rohl, A. L. The general utility lattice program (GULP). *Molecular Simulation* **29** (5), 291–341 (2003) .

[S4] Lv, W. & Henry, A. Direct calculation of modal contributions to thermal conductivity via green–kubo modal analysis. *New Journal of Physics* **18** (1), 013028 (2016) .

[S5] Lundgren, N. W., Barbalinardo, G. & Donadio, D. Mode localization and suppressed heat transport in amorphous alloys. *Physical Review B* **103** (2), 024204 (2021) .
